# *In vitro* activity of aztreonam-avibactam and comparators against *Enterobacterales* isolated from bloodstream infections: ATLAS/ surveillance, 2021–2022

**DOI:** 10.1128/spectrum.00241-26

**Published:** 2026-06-30

**Authors:** Gian Maria Rossolini, Fupin Hu, Nandini Sethuraman, Shweta Kamat, Naglaa Mohamed, Gregory Stone, Katherine K. Perez

**Affiliations:** 1 Department of Experimental and Clinical Medicine, University of Florence, and Microbiology and Virology Unit, Careggi University Hospital 415681, Florence, Italy; 2 Institute of Antibiotics, Huashan Hospital, Fudan University, Shanghai, China; 3 Key Laboratory of Clinical Pharmacology of Antibiotics, Ministry of Health, Shanghai, China; 4 Joint Laboratory of Hospital & Enterprise for Pathogen Diagnosis of Drug-resistant Bacterial Infections and Innovative Drug R&D, Shanghai, China; 5 Department of Microbiology, Central Reference Laboratory, Apollo Hospitals, Chennai, India; 6 PPIPL, Mumbai, India; 7 Pfizer Inc. 2253 , New York, New York, USA; JMI Laboratories, North Liberty, Iowa, USA

**Keywords:** aztreonam-avibactam, *Enterobacterales*, MBL, NDM, CRE

## Abstract

Carbapenem-resistant *Enterobacterales* (CRE) present a major global health concern due to rapid spread, high mortality, and limited treatment options. Metallo-β-lactamase (MBL)-producing CRE, particularly those harboring New Delhi metallo-β-lactamase (NDM) variants and co-expressing other beta-lactamases, are a significant therapeutic challenge as they exhibit resistance to most β-lactam/β-lactamase inhibitor combinations. This study evaluated the *in vitro* activity of aztreonam-avibactam (ATM-AVI) and comparators against *Enterobacterales* bloodstream isolates (*N* = 10,376) collected between 2021 and 2022 across Africa and the Middle East, Asia-Pacific, Europe, and Latin America, as part of the Antimicrobial Testing Leadership and Surveillance (ATLAS) Global Surveillance Program. Minimum inhibitory concentrations (MICs) were determined by broth microdilution, with susceptibility interpreted using 2025 European Committee on Antimicrobial Susceptibility Testing (EUCAST) criteria, as appropriate. ATM-AVI demonstrated robust activity, inhibiting 99.8% of *Enterobacterales* at a concentration of ≤4 mg/L (EUCAST susceptibility breakpoint) and maintaining activity across resistance phenotypes, including 99.6% of multi-drug resistant (MDR), 98.9% of CRE, and 99.9% of extended-spectrum beta-lactamases (ESBL) screen-positive isolates. Among MBL-positive CRE isolates, 97.0% were susceptible to ATM-AVI at the EUCAST breakpoint (≤4 mg/L), compared with 52.7% susceptible to cefiderocol at its EUCAST breakpoint (≤2 mg/L). ATM-AVI remained robust against MBL-positive isolates co-carrying ESBLs (97.8%) and other carbapenemases, like *Klebsiella pneumoniae* carbapenemase (KPC) (100%) and oxacillinase-48 (OXA-48)/OXA-48-like enzymes (98.2%). Among MBL-positive isolates co-carrying AmpC-type β-lactamases, 78.7% were susceptible to ATM-AVI at the EUCAST breakpoint. ATM-AVI demonstrated potent activity against *Enterobacterales*, including MDR, CRE, and MBL-positive phenotypes, across regions. Its ability to overcome resistance mediated by multiple β-lactamases highlights clinical potential for managing severe infections like bloodstream infections (BSIs), and the need for global surveillance data to drive clinical decision-making and regional stewardship programs.

**IMPORTANCE:**

Carbapenem-resistant *Enterobacterales* (CRE) are a serious global health threat due to their rapid spread and limited treatment options. This study highlights the promising potential of aztreonam-avibactam (ATM-AVI) as a powerful treatment against these resistant bacteria. ATM-AVI was tested on a large number of bacterial samples from various regions worldwide and showed strong effectiveness, even against strains that are resistant to most other treatments. This is particularly important for managing severe infections, such as bloodstream infections, which can be life-threatening. The study underscores the need for ongoing global surveillance to guide treatment decisions and improve health outcomes. Patients suffering from infections caused by resistant bacteria, as well as healthcare providers, could greatly benefit from the findings of this research, as it offers hope for more effective treatment options.

## INTRODUCTION

The rise of carbapenem-resistant *Enterobacterales* (CRE) is a major global health concern that has prompted both the World Health Organization and Centers for Disease Control and Prevention (CDC) to identify CRE as a critical and urgent priority pathogen for novel drug development (1–5). Recently, the incidence of CRE bloodstream infections (BSI) across Europe has increased from 2.5 per 100,000 population in 2019 to 3.97 per 100,000 in 2023 (6). The recent CDC report indicates that in the United States, CRE incidence was 18% higher in 2023 than in 2019 (7). These infections are linked to high mortality rates (30%–75%) and increased healthcare costs, primarily due to delays in effective treatment and limited therapeutic options (6, 8).

Resistance in CRE is most often mediated by carbapenemase enzymes of Ambler class A, B, and D, which are encoded by genes carried on mobile genetic elements (9, 10). Key examples of these enzymes are *Klebsiella pneumoniae* carbapenemase (KPC) from class A, metallo-beta-lactamases (MBL), such as New Delhi (NDM), Verona integron-encoded (VIM), imipenemase (IMP) from class B, and oxacillinase-48 (OXA-48) from class D (10). Among these, MBL-producing CRE pose a significant challenge due to their resistance to newly introduced β-lactam/β-lactamase (BL/BLI) combinations, including ceftazidime-avibactam, meropenem-vaborbactam, and imipenem-relebactam, in clinical settings (11). MBL-positive CRE are associated with poor clinical outcomes, emphasizing the urgent need for effective treatment strategies targeting MBLs (12). These pathogens often co-produce other beta-lactamases, such as extended-spectrum beta-lactamases (ESBL) and AmpC-type enzymes, further complicating therapeutic approaches (13). NDM-type CRE are the most prevalent globally, with a particularly high prevalence of 82.6% in Asia, largely attributed to significant contributions from India and China (14). There have also been numerous reports indicating a rise in the detection of MBL genes and isolates harboring dual carbapenemase genes, including OXA-48 and NDM, across European countries (6). The recent CDC report highlights a significant increase in the age-adjusted incidence of NDM-CRE in the US, with a 461% rise observed across 48% of states between 2019 and 2023 (7). Factors, such as travel, international trade, and healthcare exposure, have facilitated the global spread of NDM-producing CRE, contributing to the changing epidemiology of carbapenemase-producing CRE (CP-CRE) (15).

Aztreonam-avibactam (ATM-AVI) is a novel BL/BLI combination, demonstrating efficacy against MBL-positive CRE by leveraging aztreonam’s stability against MBLs and avibactam’s activity as an inhibitor of serine beta-lactamases (1, 16). ATM-AVI has demonstrated potent *in vitro* activity against MBL-positive *Enterobacterales* (10, 17–19). In a Phase 3 REVISIT trial, ATM-AVI ± metronidazole achieved clinical cure rates comparable to meropenem ± colistin for complicated intra-abdominal infections and hospital-acquired pneumonia (20). Additionally, in the ASSEMBLE Phase 3 trial, ATM-AVI ± metronidazole was effective in treating patients with infections caused by MBL-positive Gram-negative bacteria (21). These findings supported regulatory approval of ATM-AVI by the European Medicines Agency in 2024 (22) and the United States Food and Drug Administration (US FDA) in 2025 (23).

This study evaluates the *in vitro* activity of ATM-AVI and comparators against *Enterobacterales*, including MBL-positive CRE, isolated from BSIs between 2021 and 2022 across Africa and the Middle East, Asia-Pacific, Europe, and Latin America, as part of the Antimicrobial Testing Leadership and Surveillance (ATLAS) Global Surveillance Program (24).

## MATERIALS AND METHODS

### Bacterial isolates

Predetermined counts of clinical *Enterobacterales* isolates (*N* = 10,376) were collected from non-duplicate hospitalized patients at participating centers between 2021 and 2022 across Africa/Middle East (AfME), Asia-Pacific (APAC), Europe, and Latin America, per the ATLAS protocol (24). The standardized protocol specifies target numbers of isolates by genus and/or species but does not impose restrictions on infection source or collection unit/wards. Only bloodstream isolates considered to be causative for infection were included in this analysis. The isolates represented diverse genera of *Enterobacterales*, including *Escherichia* spp., *Citrobacter* spp., *Enterobacter* spp., *Klebsiella* spp., *Proteus* spp., *Serratia* spp., *Morganella* spp., and *Providencia* spp. Each isolate was sent to a central reference laboratory (IHMA, Schaumburg, IL, USA) for confirmation and antimicrobial susceptibility testing; species identification was confirmed by MALDI-TOF mass spectrometry (Bruker Daltonics, Billerica, MA, USA).

### Antimicrobial susceptibility testing

Minimum inhibitory concentrations (MICs) were determined by broth microdilution following CLSI guidelines (25). ATM-AVI and a panel of comparator antimicrobial agents, including aztreonam, cefepime, colistin, meropenem, tigecycline, amikacin, ceftazidime-avibactam, ceftolozane-tazobactam, and piperacillin-tazobactam, were tested against *Enterobacterales* isolates (*N* = 10,376). For the ATLAS program years 2021 and 2022, cefiderocol, ertapenem, minocycline, and tobramycin were not part of the routine testing panel. Hence, the antimicrobial activity of ATM-AVI and comparator agents (amikacin, aztreonam, cefiderocol, colistin, ertapenem, minocycline, tigecycline, and tobramycin) was evaluated separately against *Enterobacterales* that were MBL-positive. Susceptibility was interpreted primarily using 2025 European Committee on Antimicrobial Susceptibility Testing (EUCAST) breakpoints (ATM-AVI, S ≤ 4 mg/L; R > 4 mg/L) (26); FDA and CLSI criteria were applied for tigecycline (S ≤ 2 mg/L; I 4 mg/L; R ≥ 8 mg/L) and minocycline (S ≤ 4 mg/L; I 8 mg/L; R ≥ 16 mg/L).

Isolates were categorized as MDR according to criteria published in 2012 by the joint European and United States Centers for Disease Control, which define an MDR isolate as one that is nonsusceptible to ≥1 agent in ≥3 antimicrobial classes (17). The isolates that qualified for ESBL screening were *Escherichia coli*, *K. pneumoniae*, *Klebsiella oxytoca*, *Klebsiella variicola*, and *Proteus mirabilis* isolates with ceftazidime MIC ≥ 2 mg/L and/or aztreonam MIC ≥ 2 mg/L (24). Confirmatory testing with clavulanate was not performed, and cefotaxime was not included in the testing panel. As these phenotypic criteria may also identify plasmid-mediated AmpC producers or TEM/SHV overexpressors, the isolates were classified as ESBL screen-positive rather than confirmed ESBL-positive for the purpose of this analysis.

*Enterobacterales* isolates with meropenem MIC > 8 mg/L were categorized as CRE according to the 2025 EUCAST breakpoints (26). Isolates with meropenem MIC ≥ 2 mg/L were considered candidates for carbapenemase screening (27) and were routinely subjected to molecular characterization following the ATLAS protocol, except for isolates from the Chinese mainland.

### Molecular analysis

Multiplex PCR and DNA sequencing were used to detect and characterize beta-lactamase genes. Isolates containing one or more of the major MBL genes, like NDM, IMP, and VIM, were considered MBL-positive. The presence of serine carbapenemase genes, namely KPC, OXA-48, and OXA-48-like variants (e.g., OXA-163, OXA-181, OXA-232, and OXA-244 [28]), ESBL genes (SHV, TEM, CTX-M-group—1, 3, 9, 14, 15, 65, 139, 189, 231, LAP, and GES), and acquired AmpC β-lactamases (ACC, ACT, CMY, DHA, CFE) in MBL-positive isolates was also documented. Gene variants, including specific NDM variants, were identified by sequencing and BLAST analysis against reference databases (National Center for Biotechnology Information database [www.ncbi.nlm.nih.gov]). Molecular characterization data for CRE isolates were not available for the Chinese mainland in the surveillance program. For this study, a random cohort of CRE isolates collected during the same period (2021–2022) in the Chinese mainland underwent molecular characterization, and the identified MBL-positive isolates were integrated into the larger MBL-positive cohort to enhance the surveillance scope in the APAC region. This study did not assess non-enzymatic mechanisms of resistance to ATM-AVI, including insertions in Penicillin-Binding Protein 3 (PBP3) or porin and efflux pump modifications.

## RESULTS

### Distribution of *Enterobacterales*

A total of 10,376 *Enterobacterales* isolates from patients with BSIs were collected from 59 countries representing AfME (*n* = 1,034), APAC (*n* = 2,431), Europe (*n* = 5,253), and Latin America (*n* = 1,658) through the ATLAS program in 2021–2022. The majority of *Enterobacterales* isolates were obtained from non-ICU wards, comprising 71.5% of the total, with regional variations ranging from 59.8% to 76.3% (Table S1). In accordance with ATLAS’s pathogen-based sampling framework, *Escherichia coli* (37.0%) and *Klebsiella pneumoniae* (31.9%) were most frequently identified in this BSI cohort. Further species-level pathogen distribution details can be found in Table S2.

**Fig 1:**
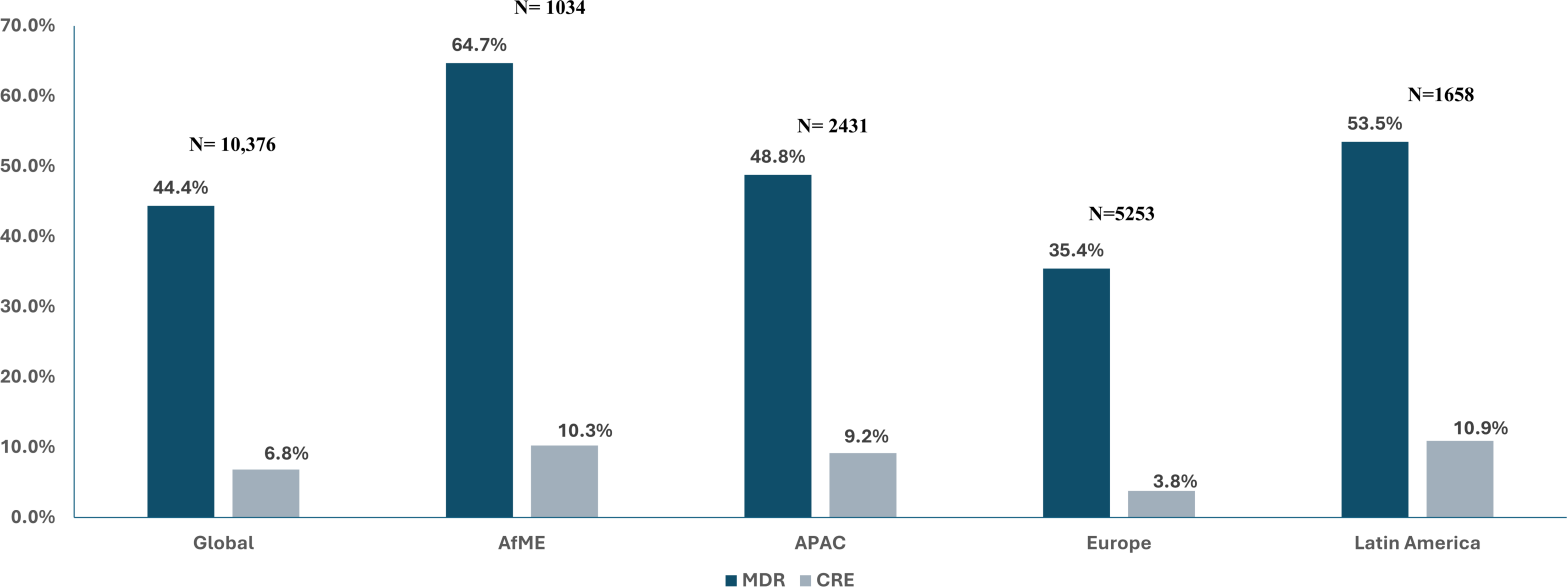
Proportions of MDR and CRE phenotypes among *Enterobacterales* isolates collected from bloodstream infections overall and across different regions from 2021 to 2022. Phenotypes are mutually inclusive. Overall includes isolates from AfME, APAC, Europe, and Latin America. *Enterobacterales* included the following genera: *Escherichia*, *Citrobacter*, *Enterobacter*, *Klebsiella*, *Proteus*, *Providencia*, *Morganella,* and *Serratia*. AfME, Africa and Middle East; APAC, Asia Pacific; CRE, carbapenem-resistant *Enterobacterales*; MDR, multi-drug resistant.

**Fig 2:**
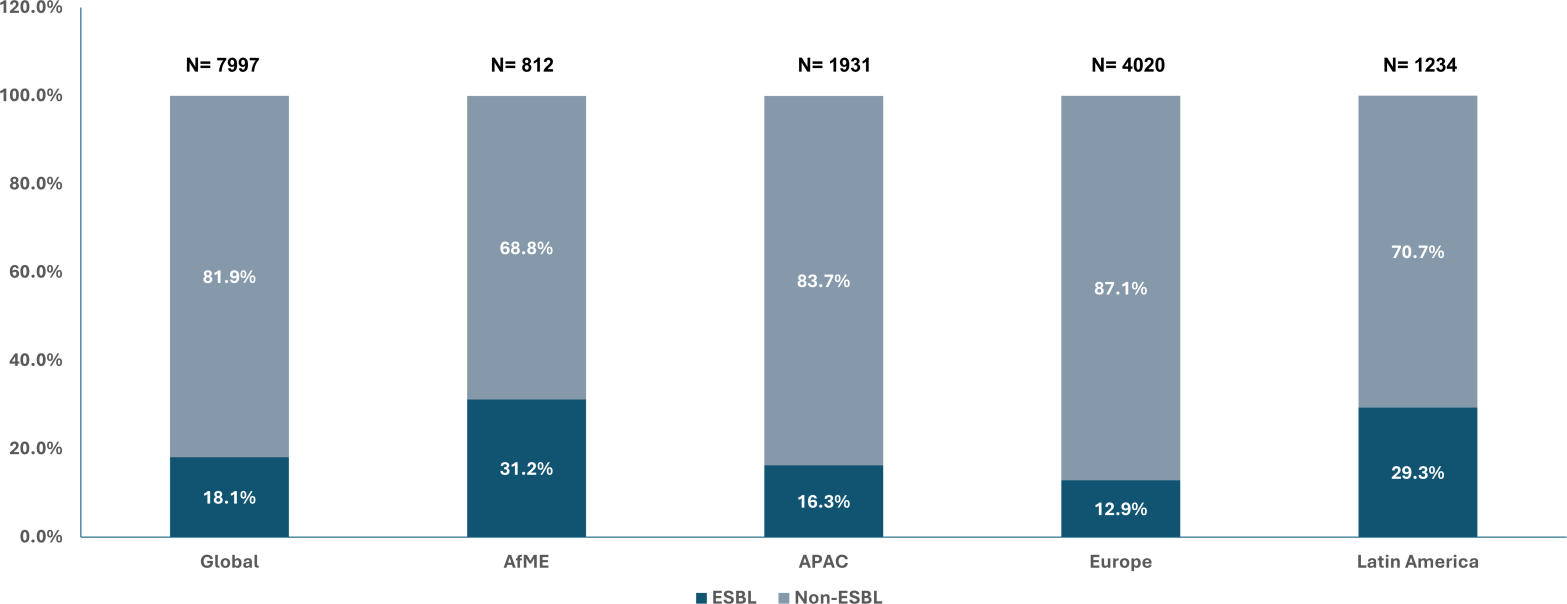
Proportion of ESBL screen-positive *Enterobacterales* collected from bloodstream infections overall and across different regions from 2021 to 2022. Overall includes isolates from AfME, APAC, Europe, and Latin America. AfME, Africa and Middle East; APAC, Asia Pacific; ESBL, extended-spectrum β-lactamase. Includes *Enterobacterales* that qualified for β-lactamase screening: *Escherichia coli*, *Klebsiella oxytoca*, *Klebsiella pneumoniae*, *Klebsiella variicola*, *Proteus mirabilis*.

MDR represented 44.4% of the total isolates. The highest MDR rate was observed in the AfME region at 64.7% (669/1,034), followed by Latin America at 53.5% (887/1,658), APAC at 48.8% (1,186/2,431), and Europe at 35.4% (1,862/5,253) (Fig. 1). The overall proportion of ESBL screen-positive isolates was 18.1% (1,447/7,997), being variable across regions with AfME at 31.2% (253/812), APAC at 16.3% (314/1,931), Europe at 12.9% (518/4,020), and Latin America at 29.3% (362/1,234) (Fig. 2). As cefotaxime was not included in the testing panel, this may underestimate true ESBL prevalence, particularly in regions where CTX-M enzymes predominate.

**Fig 3:**
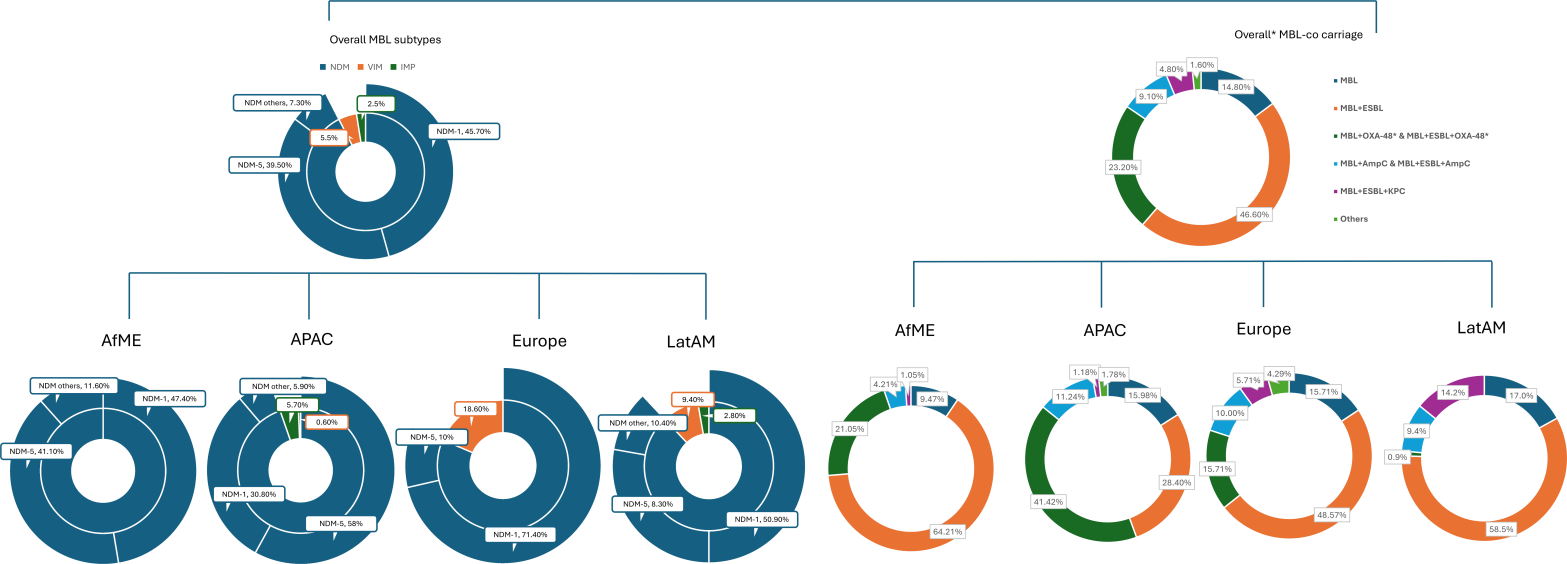
Genotypic distribution of MBL-producing *Enterobacterales* isolated from BSI collected overall and across regions between 2021 and 2022. MBL-positive *Enterobacterales* were identified among isolates with meropenem MIC ≥ 2 mg/L that were considered candidates for carbapenemase screening. *Overall includes isolates from AfME, APAC (including the Chinese mainland), Europe, and Latin America. Global does not include the US and Canada. *Enterobacterales* included the following genera: *Escherichia*, *Citrobacter*, *Enterobacter*, *Klebsiella*, *Proteus*, *Providencia*, *Morganella*, and *Serratia*. OXA-48-like constitutes OXA-163, OXA-181, OXA-232, and OXA-244. Includes *Escherichia coli*, *Klebsiella oxytoca*, *Klebsiella pneumoniae*, *Klebsiella varicella*, and *Proteus mirabilis*. NDM subtype proportions shown as a percentage of total MBL-positive isolates (*n* = 440) for visual context; subtype proportions among NDM-positive isolates only (*n* = 407) are reported in text as NDM-1 (49.4%) and NDM-5 (42.8%). AfME, Africa and Middle East; APAC, Asia Pacific; ESBL, extended-spectrum β-lactamase; IMP, imipenemase metallo-β-lactamase; KPC, *Klebsiella pneumoniae* carbapenemase; MBL, metallo-β-lactamase; NDM, New Delhi metallo-β-lactamase; OXA-48-like, oxacillinase-48-like β-lactamase; VIM, Verona integron-encoded metallo-β-lactamase.

CRE defined by the EUCAST 2025 meropenem resistant breakpoint (MIC > 8 mg/L) represented 6.8% (710/10,376) of the total isolates, with AfME (10.3%, 106/1,034), Latin America (10.9%, 181/1,658), and APAC (9.2%, 223/2,431) reporting higher CRE rates compared to Europe (3.8%, 200/5,253) (Fig. 1). Out of the 710 isolates of CRE, data on CP-CRE were available for 665 isolates in the ATLAS surveillance database, as genotypic characterization data for the 45 CRE isolates from Chinese mainland were not included in the database. Among these, 650 isolates were identified as CP-CRE, with MBL being the most prevalent type of carbapenemase at 52.9% (344/650), and within the MBL category, the NDM subtype was the most prevalent, accounting for 50.8% (330/650) of isolates. The APAC and AfME regions exhibited the highest prevalence of NDM-CRE, at 76.3% (132/173) and 73.8% (76/103), respectively. In contrast, Europe and Latin America showed lower prevalence at 24.1% (48/199) and 42.3% (74/175), respectively. APAC also had the highest proportion of CRE positive for the OXA-48 and OXA-48-like genotypes, at 63.6% (110/173), followed by AfME at 46.6% (48/103) and Europe at 31.2% (62/199), resulting in an overall prevalence of 34.2% (222/650). KPC-CRE were more prevalent in Latin America (61.1%, 107/175) and Europe (50.8%, 101/199) compared to APAC and AfME, which had a combined prevalence of 32.5% (211/650) (Table 1). When using meropenem, MIC of ≥2 mg/L and including isolates obtained from the Chinese mainland (*N* = 16), a total of 440 isolates were identified as MBL-positive. NDM-positive was the most frequently detected genotype, representing 92.5% (407/440) of the isolates, with regional prevalence ranging from 81.4% to 100%, largely exceeding VIM- and IMP-positive isolates. Most NDM-positive isolates comprised of NDM-1 (49.4%, 201/407) and NDM-5 (42.8%, 174/407). NDM-1 was predominantly found in Europe (87.7%, 50/57) and NDM-5 was more common in APAC (61.3%, 98/160) (Fig. 3). Other NDM subtypes constitute 7.9% of the total NDM-positive isolates, with their overall and regional distribution detailed in Table S3. VIM accounted for 5.5% (24/440) overall, with higher prevalence in Europe (18.6%, 13/70) and Latin America (9.4%, 10/106) (Fig. 3).

**TABLE 1:** Genotypic distribution of CRE *Enterobacterales* isolated from BSI collected overall and across regions between 2021 and 2022^*f*^^,^^*g*^

	Overall^*a*^	AfME	APAC	Europe	Latin America
CRE isolates (N)^***b***^	710	106	223	200	181
CP-CRE isolates (N)	650^*d*^	103	173	199	175
MBL [*n* (%) of CP-CRE]	344 (52.9)^*e*^	76 (73.8)	133 (76.9)	53 (26.6)	82 (46.9)^*d*^
NDM [*n* (%)]	330 (50.8)	76 (73.8)	132 (76.3)	48 (24.1)	74 (42.3)
VIM [*n* (%)]	16 (2.5)	0	1 (0.6)	5 (2.5)	10 (5.7)
IMP [*n* (%)]	0	0	0	0	0
OXA-48 and OXA-48-like^*c*^ [*n* (%) of CP-CRE]	222 (34.2)	48 (46.6)	110 (63.6)	62 (31.2)	2 (1.1)
KPC [*n* (%) of CP-CRE]	211 (32.5)	0	3 (1.7)	101 (50.8)	107 (61.1)

^*a*^ Overall includes isolates from AfME, APAC, Europe, and Latin America.

^*b*^ *Enterobacterales* included the following genera: *Escherichia*, *Citrobacter*, *Enterobacter*, *Klebsiella*, *Proteus*, *Providencia*, *Morganella*, and *Serratia.*

^*c*^ OXA-48-like constitutes OXA-163, OXA-181, OXA-232, and OXA-244.

^*d*^ Data on CP-CRE were available for 665 out of 710 CRE isolates in the ATLAS surveillance database, excluding genotypic characterization data for 45 isolates from the Chinese mainland.

^*e*^ Two isolates from Latin America co-carried NDM and VIM genes.

^*f*^ *Enterobacterales* were categorized as CRE according to the 2025 EUCAST meropenem (MIC > 8 mg/L) breakpoints.

^*g*^ AfME, Africa and Middle East; APAC, Asia Pacific; CRE, carbapenem-resistant *Enterobacterales*; IMP, imipenemase metallo-β-lactamase; KPC, *Klebsiella pneumoniae* carbapenemase; NDM, New Delhi metallo-β-lactamase; OXA-48-like, oxacillinase-48-like β-lactamase; VIM, Verona integron-encoded metallo-β-lactamase.

Overall, MBL+ESBL was the most common co-carriage pattern among MBL-positive isolates (46.6%), followed by the combined MBL+OXA-48/OXA-48-like co-carriage category (23.2%) and MBL alone (14.8%). This combined MBL+OXA-48/OXA-48-like category was more prevalent than MBL+ESBL+KPC overall (23.2% vs 4.8%) and across all regions (21.1%–41.4% vs 1.1%−5.7%), except in Latin America, where MBL+ESBL+KPC was more common (14.2% vs 0.9%). MBL+AmpC-associated co-carriage accounted for 9.1% of isolates, whereas other patterns were uncommon (1.6%). (Fig. 3). Regarding species distribution, *K. pneumoniae* and *E. coli* constituted 69.5% (306/440) and 11.1% (49/440) of MBL-positive isolates, respectively. Notably, NDM-5 was more prevalent in *E. coli* at 83.7% (41/49), whereas both NDM-1 and NDM-5 were frequent in *K. pneumoniae* (46.4%, 142/306 and 41.2%, 126/306, respectively) (Table S4).

### Antimicrobial activity of ATM-AVI and other antimicrobials against *Enterobacterales*

Overall, 99.8% (*N* = 10, 376) of isolates were inhibited by ATM-AVI at a concentration of ≤4 mg/L (EUCAST clinical breakpoint for susceptibility), with MIC_50_ and MIC_90_ values of 0.06 and 0.25 mg/L, respectively. Among comparator antimicrobials, meropenem, tigecycline, amikacin, and ceftazidime/avibactam demonstrated high activity, with susceptibility rates >90% (Table 2). ATM-AVI demonstrated potent antimicrobial activity against *E. coli* and *K. pneumoniae*, with susceptibility rates of 99.7% (MIC_50/90_, 0.03/0.06 mg/L) and 99.9% (MIC_50/90_, 0.06/0.25 mg/L), respectively, with only 10 *E. coli* and 3 *K. pneumoniae* isolates exhibiting MICs > 4 mg/L. Among comparator antimicrobials, colistin, meropenem, tigecycline, amikacin, ceftazidime/avibactam, and ceftolozane/tazobactam demonstrated high activity, with susceptibility >90% against *E. coli*, while only colistin, tigecycline, and ceftazidime/avibactam exhibited high activity (>90% susceptibility) against *K. pneumoniae* (Table S5). ATM-AVI showed similar activity across all regions, inhibiting >99% of isolates at a concentration of ≤4 mg/L, while some geographic variability was observed with activities of meropenem, tigecycline, amikacin, and ceftazidime/avibactam (Table S6).

**TABLE 2:** Antimicrobial activity of aztreonam-avibactam and comparator agents against all *Enterobacterales* isolated from BSI between 2021 and 2022^*e*^

Antimicrobial agent	Susceptibility (EUCAST)		MIC (mg/L)		No. of isolates inhibited at respective MIC (mg/L) (% cumulative inhibition)										
	%S	%R	MIC_50_	MIC_90_	≤0.06	0.12	0.25	0.5	1	2	4	8	16	32	>32
Overall^*a*^ (*N* = 10,376)**^*b*^**															
Aztreonam/avibactam	99.8	0.2	0.06	0.25	7,821 (75.4)	1,435 (89.2)	573 (94.7)	309 (97.7)	115 (98.8)	55 (99.3)	50 (99.8)	11 (99.9)	1 (99.9)	1 (99.9)	5 (100)
Aztreonam	66.4	30.9	0.12	128	3,733 (36.0)	2,176 (56.9)	650 (63.2)	189 (65.0)	137 (66.4)	118 (67.5)	165 (69.1)	262 (71.6)	400 (75.5)	652 (81.7)	1,894 (100)
Cefepime	68.9	26.4	0.12	64	0	6,358 (61.3)	348 (64.6)	248 (67.0)	193 (68.9)	190 (70.7)	299 (73.6)	426 (77.7)	403 (81.6)	472 (86.1)	1,439 (100)
Colistin	86.7^*d*^	13.3	0.25	16	0	3,915 (37.7)	4,554 (81.6)	362 (85.1)	120 (86.3)	45 (86.7)	56 (87.2)	61 (87.8)	1,263 (100)		
Meropenem	91.8	6.8	0.06	0.5	8,863 (85.4)	325 (88.5)	122 (89.7)	64 (90.3)	71 (91.0)	77 (91.8)	61 (92.4)	83 (93.2)	128 (94.4)	582 (100)	
Tigecycline^*c*^	97.4^*d*^	0.4	0.5	1	130 (1.2)	1,896 (19.5)	2,877 (47.2)	3,033 (76.5)	1,531 (91.2)	636 (97.4)	234 (99.6)	30 (99.9)	9 (100)		
Amikacin	92.8	7.2	2	8	0	0	24 (0.2)	338 (3.5)	3,069 (33.1)	3,830 (70.1)	1,790 (87.2)	578 (92.8)	212 (94.8)	96 (95.8)	439 (100)
Ceftazidime/avibactam	95.5	4.5	0.12	1	2,663 (25.7)	3,717 (61.5)	2,002 (80.8)	827 (88.8)	424 (92.8)	185 (94.6)	70 (95.3)	16 (95.4)	4 (95.5)	5 (95.5)	463 (100)
Ceftolozane/tazobactam	84.9	15.1	0.5	32	16 (0.2)	592 (5.9)	3,997 (44.4)	2,773 (71.1)	1,067 (81.4)	365 (84.9)	218 (87.0)	169 (88.6)	128 (89.9)	1,051 (100)	
Piperacillin/tazobactam	77	23.0	2	128	0	0	0	653 (6.3)	1,354 (19.3)	3,589 (53.9)	1,773 (71.0)	616 (77.0)	431 (81.1)	275 (83.8)	1,685 (100)

^*a*^ Overall includes isolates from AfME, APAC, Europe, and Latin America.

^*b*^ *Enterobacterales* included the following genera: *Escherichia* (37.2%), *Citrobacter* (2.7%), *Enterobacter* (9.4%), *Klebsiella* (39.9%), *Proteus* (3.8%), *Providencia* (1.1%), *Morganella* (1.8%), and *Serratia* (4.7%).

^*c*^ FDA breakpoints have been used.

^*d*^ Intrinsic resistance to colistin and tigecycline has been noted in *Proteus *spp.*, Providencia *spp*., Morganella morganii*, and *Serratia marcescens *and may contribute to elevated MIC values.

^*e*^ EUCAST, European Committee on Antimicrobial Susceptibility Testing; MIC, minimum inhibitory concentration; MIC_50_, minimum concentration required to inhibit 50% of the organisms; MIC_90_, minimum concentration required to inhibit 90% of the organisms; S, susceptibility; R, resistance.

### Antimicrobial activity of ATM-AVI and other antimicrobials against MDR isolates

ATM-AVI demonstrated consistently high activity against 4,604 MDR isolates, with a susceptibility rate of 99.6% at a concentration of ≤4 mg/L (MIC_50_/MIC_90_, 0.06/0.5 mg/L). Among comparators, only tigecycline inhibited >90% of isolates at a concentration of ≤2 (FDA clinical breakpoint for susceptibility) (Table 3). ATM-AVI inhibited 99.4% of *E. coli* isolates at a concentration of ≤4 mg/L, with MIC_50_ and MIC_90_ values of 0.06 mg/L and 0.12 mg/L, respectively. Tigecycline (99.7%), colistin (99.3%), meropenem (96.9%), and amikacin (93.1%) also exhibited high activity against *E. coli* isolates. ATM-AVI displayed robust activity against *K. pneumoniae* isolates, inhibiting 99.9% at a concentration of <4 mg/L, with MIC_50_ and MIC_90_ values of 0.12 and 0.5 mg/L. Among comparators, tigecycline (95.0%) and colistin (91.2%) showed potent activity (Table S7). Across all regions, ATM-AVI inhibited >99% of MDR isolates at a concentration of ≤4 mg/L, with MIC_50_ of 0.06 mg/L and MIC_90_ ranging between 0.25 and 0.5 mg/L (Table S8).

**TABLE 3:** Antimicrobial activity of aztreonam-avibactam and comparator agents against MDR *Enterobacterales* isolated from BSI between 2021 and 2022^*d*^

Antimicrobial agent	Susceptibility (EUCAST)		MIC (mg/L)		No. of isolates inhibited at respective MIC (mg/L) (% cumulative inhibition)										
	%S	%R	MIC_50_	MIC_90_	≤0.06	0.12	0.25	0.5	1	2	4	8	16	32	>32
MDR EUCAST (*N* = 4,604)**^*a*^**															
Aztreonam/avibactam	99.6	0.6	0.06	0.5	2,685 (58.3)	881 (77.4)	509 (88.5)	301 (95)	111 (97.5)	54 (98.6)	46 (99.6)	10 (99.8)	1 (99.9)	1 (99.9)	5 (100)
Aztreonam	25.4	69.5	32	128	355 (7.7)	440 (17.3)	182 (21.2)	108 (23.6)	84 (25.4)	89 (27.3)	144 (30.4)	258 (36.1)	400 (44.7)	650 (58.9)	1,894 (100)
Cefepime	30.6	59.4	16	64	0	875 (19.0)	177 (22.8)	203 (27.3)	155 (30.6)	172 (34.4)	285 (40.5)	423 (49.7)	403 (58.5)	472 (68.7)	1,439 (100)
Colistin	86.9^*c*^	13	0.25	16	0	1,625 (35.3)	2,102 (80.9)	191 (85.0)	60 (86.4)	25 (86.9)	49 (88.0)	51 (89.0)	501 (100)		
Meropenem	81.4	15.4	0.06	32	3,242 (70.4)	206 (74.9)	95 (76.9)	62 (78.3)	68 (79.8)	77 (81.4)	61 (82.8)	83 (84.6)	128 (87.4)	582 (100)	
Tigecycline^*b*^	96.2^*c*^	0.6	0.5	2	35 (0.8)	647 (14.9)	1,107 (38.9)	1,326 (67.7)	919 (87.6)	395 (96.2)	146 (99.4)	23 (99.9)	6 (100)		
Amikacin	84.3	15.7	2	32	0	0	4 (0.1)	88 (2.0)	822 (19.9)	1,474 (51.9)	1,044 (74.5)	450 (84.3)	193 (88.5)	92 (90.5)	437 (100)

^*a*^ *Enterobacterales* included the following genera: *Escherichia* (37.7%), *Citrobacter* (1.4%), *Enterobacter* (9.4%), *Klebsiella* (43.7%), *Proteus* (2.4%), *Providencia* (1.1%), *Morganella* (1.2%), and *Serratia* (3.2%).

^*b*^ FDA breakpoints have been used.

^*c*^ Intrinsic resistance to colistin and tigecycline has been noted in *Proteus *spp.*, Providencia *spp*., M. morganii*, and *S. marcescens *and may contribute to elevated MIC values.

^*d*^ EUCAST, European Committee on Antimicrobial Susceptibility Testing; MDR, multi-drug resistant; MIC, minimum inhibitory concentration; MIC_50_, minimum concentration required to inhibit 50% of the organisms; MIC_90_, minimum concentration required to inhibit 90% of the organisms; S, susceptibility; R, resistance.

### Antimicrobial activity of ATM-AVI and other antimicrobials against ESBL screen-positive isolates

ATM-AVI exhibited robust activity against 1,447 isolates meeting ESBL screening criteria (which may include ESBL producers, as well as plasmid-mediated AmpC and other β-lactamase producers), inhibiting 99.9% of isolates at a concentration of ≤4 mg/L with MIC_50_ and MIC_90_ values of 0.12 and 0.5 mg/L. Among comparators, tigecycline (96.5%) and colistin (91.4%) displayed strong activity (Table 4). ATM-AVI inhibited 99.8% (MIC_50/90_, 0.06/0.12 mg/L) of *E. coli* isolates and all *K. pneumoniae* isolates (MIC_50_/_90_, 0.12/0.5 mg/L) at a concentration of ≤4 mg/L. Meropenem inhibited 94.3% of *E. coli* isolates but showed a lower activity against *K. pneumoniae*, inhibiting 45.9% of isolates (Table S9). Tigecycline and colistin maintained high potent activity against both *E. coli* and *K. pneumoniae* isolates. Geographically, ATM-AVI displayed excellent activity, with susceptibility rates of 100% in AfME, Europe, and Latin America, and 99.7% in the APAC region at a concentration of ≤4 mg/L (Table S10).

**TABLE 4:** Antimicrobial activity of aztreonam-avibactam and comparator agents against ESBL-screen positive *Enterobacterales* isolated from BSI between 2021 and 2022^*d*^

Antimicrobial agent	Susceptibility (EUCAST)		MIC (mg/L)		No. of isolates inhibited at respective MIC (mg/L) (% cumulative inhibition)										
	%S	%R	MIC_50_	MIC_90_	≤0.06	0.12	0.25	0.5	1	2	4	8	16	32	>32
ESBL Isolates (*N* = 1,447)**^*a*^**															
Aztreonam/avibactam	99.9	0.1	0.12	0.5	722 (49.9)	305 (71.0)	258 (88.8)	117 (96.9)	22 (98.4)	12 (99.2)	10 (99.9)	0 (99.9)	0 (99.9)	0 (99.9)	1 (100)
Aztreonam	0.4	96.6	64	128	0	0	1 (0.1)	0 (0.1)	4 (0.4)	12 (1.2)	32 (3.4)	87 (9.4)	135 (18.7)	258 (36.6)	918 (100)
Cefepime	1.6	90.3	64	64	0	4 (0.3)	2 (0.4)	3 (0.6)	14 (1.6)	29 (3.6)	88 (9.7)	138 (19.2)	177 (31.4)	217 (46.4)	775 (100)
Colistin	91.4^*c*^	8.6	0.25	1	0	478 (33.0)	751 (84.9)	52 (88.5)	25 (90.3)	16 (91.4)	21 (92.8)	32 (95.0)	72 (100)		
Meropenem	62.1	32.2	0.06	32	793 (54.8)	21 (56.3)	12 (57.1)	8 (57.6)	20 (59.0)	44 (62.0)	35 (64.5)	48 (67.8)	80 (73.3)	386 (100)	
Tigecycline^*b*^	96.5^*c*^	0.5	0.5	2	8 (0.6)	193 (13.9)	327 (36.5)	400 (64.1)	337 (87.4)	132 (96.5)	43 (99.5)	6 (99.9)	1 (100)		
Amikacin	72.8	27.2	4	128	0	0	1 (0.1)	22 (1.6)	186 (14.4)	347 (38.4)	334 (61.5)	164 (72.8)	80 (78.4)	45 (81.5)	268 (100)

^*a*^ Includes *Escherichia coli* (32.6%),* Klebsiella oxytoca* (0.8%),* Klebsiella pneumoniae* (65.5%),* Klebsiella variicola* (0.8%),* Proteus mirabilis* (0.3%).

^*b*^ FDA breakpoints have been used.

^*c*^ Intrinsic resistance to colistin and tigecycline has been noted in *Proteus *spp.,* Providencia *spp*.*,* M. morganii*, and *S. marcescens *and may contribute to elevated MIC values.

^*d*^ EUCAST, European Committee on Antimicrobial Susceptibility Testing; ESBL, extended-spectrum β-lactamase; MIC, minimum inhibitory concentration; MIC_50_, minimum concentration required to inhibit 50% of the organisms; MIC_90_, minimum concentration required to inhibit 90% of the organisms; S, susceptibility; R, resistance.

### Antimicrobial activity of ATM-AVI and other antimicrobials against CRE phenotypes

ATM-AVI demonstrated high activity against 710 CRE isolates, inhibiting 98.9% of isolates at a concentration of ≤4 mg/L and achieving MIC_50_ and MIC_90_ of 0.25 and 1 mg/L. Comparator agents exhibited lower activity, with only tigecycline covering >90% of isolates (Table 5). ATM-AVI inhibited 82.2% of CRE *E. coli* isolates at the EUCAST breakpoint of ≤4 mg/L, with MIC_50_ and MIC_90_ values of 2 and 8 mg/L, respectively. ATM-AVI inhibited all CRE *K. pneumoniae* isolates, with MIC_50_ and MIC_90_ values of 0.25 and 0.5 mg/L, respectively (Table S11).

**TABLE 5:** Antimicrobial activity of aztreonam-avibactam and comparator agents against CRE *Enterobacterales* isolated from BSI between 2021 and 2022^*d*^

Antimicrobial agent	Susceptibility (EUCAST)		MIC (mg/L)		No. of isolates inhibited at respective MIC (mg/L) (% cumulative inhibition)										
	%S	%R	MIC_50_	MIC_90_	≤0.06	0.12	0.25	0.5	1	2	4	8	16	32	>32
CRE isolates (*N* = 710)**^*a*^**															
Aztreonam/avibactam	98.9	1.1	0.25	1	62 (8.7)	140 (28.5)	266 (65.9)	158 (88.2)	44 (94.4)	14 (96.3)	18 (98.9)	5 (99.6)	0 (99.6)	0 (99.6)	3 (100)
Aztreonam	5.6	93.4	128	128	7 (1)	5 (1.7)	10 (3.1)	14 (5.1)	4 (5.6)	1 (5.8)	6 (6.6)	4 (7.2)	9 (8.5)	23 (11.7)	627 (100)
Cefepime	0.1	99.3	64	64	0	0	0	0	1 (0.1)	1 (0.3)	3 (0.7)	9 (2.0)	12 (3.7)	47 (10.28)	637 (100)
Colistin	80.1^*c*^	19.9	0.25	16	0	183 (25.8)	315 (70.1)	36 (75.2)	21 (78.2)	14 (80.1)	19 (82.8)	33 (87.5)	89 (100)		
Meropenem	0	100	32	32	0	0	0	0	0	0	0	0	128 (18.0)	582 (100)	
Tigecycline^*b*^	93^*c*^	1.3	1	2	0	15 (2.1)	58 (10.3)	193 (37.5)	257 (73.7)	137 (93.0)	41 (98.7)	7 (99.7)	2 (100)		
Amikacin	43.1	56.9	16	128	0	0	0	4 (0.6)	60 (9.0)	63 (17.9)	100 (32)	79 (43.1)	58 (51.3)	45 (57.6)	301 (100)

^*a*^ *Enterobacterales* included the following genera: *Escherichia* (6.3%), *Citrobacter* (0.6%), *Enterobacter* (3%), *Klebsiella* (86.2%), *Proteus* (0.4%), *Providencia* (1%), *Morganella* (0.1%), and *Serratia* (2.4%).

^*b*^ FDA breakpoints have been used.

^*c*^ Intrinsic resistance to colistin and tigecycline has been noted in *Proteus *spp.*, Providencia *spp*., M. morganii*, and *S. marcescens *and may contribute to elevated MIC values.

^*d*^ CRE, carbapenem-resistant *Enterobacterales*; EUCAST, European Committee on Antimicrobial Susceptibility Testing; MIC, minimum inhibitory concentration; MIC_50_, minimum concentration required to inhibit 50% of the organisms; MIC_90_, minimum concentration required to inhibit 90% of the organisms; S, susceptibility; R, resistance.

### Antimicrobial activity of ATM-AVI and other antimicrobials against MBL-positive *Enterobacterales,* including co-producers

ATM-AVI maintained potent activity against 440 MBL-positive *Enterobacterales*, with MIC_50_ and MIC_90_ values of 0.25 and 1 mg/L, respectively, inhibiting 97.0% of isolates at a concentration of ≤4 mg/L. Among comparators, tigecycline (97.5%) and colistin (80.5%) were active, while cefiderocol showed moderate activity (52.7%) (Table 6). ATM-AVI inhibited 75.5% of MBL-positive *E. coli* isolates at a concentration of ≤4 mg/L (MIC_50/90_, 2/16 mg/L), whereas tigecycline and colistin inhibited all isolates. Only 36.7% of MBL-positive *E. coli* isolates were susceptible to cefiderocol according to EUCAST breakpoints. All MBL-positive *K. pneumoniae* isolates were inhibited by ATM-AVI at the EUCAST breakpoint of ≤4 mg/L (MIC_50/90_, 0.25/0.5 mg/L). Tigecycline, colistin, and cefiderocol inhibited 98%, 79.7%, and 55.9% of isolates, respectively (Table S13). ATM-AVI inhibited 100% of MBL-positive *Enterobacterales* from AfME, Europe, and Latin America, while 92.3% were inhibited from the APAC region (EUCAST breakpoint of ≤4 mg/L) (Table S14).

**TABLE 6:** Antimicrobial activity of aztreonam-avibactam and comparator agents against MBL-positive *Enterobacterales* isolated from BSI between 2021 and 2022^*e*^

Antimicrobial agent	Susceptibility (EUCAST)		No. of isolates inhibited at respective MIC (mg/L) (% cumulative inhibition)										
	%S	%R	≤0.06	0.12	0.25	0.5	1	2	4	8	16	32	>32
MBL-positive (*N* = 440)**^*a*^**													
Aztreonam/avibactam	97	3	78 (17.7)	73 (34.3)	107 (58.6)	112 (84.1)	34 (91.8)	11 (94.3)	12 (97.0)	7 (98.6)	4 (99.5)	0 (99.5)	2 (100)
Aztreonam	10.9	86.4	13 (2.9)	5 (4.1)	15 (7.5)	7 (9.1)	8 (10.9)	6 (12.3)	6 (13.6)	9 (15.7)	6 (17.0)	30 (23.9)	335 (100)
Cefepime	0.2	98.9	0	1 (0.2)	0 (0.2)	0 (0.2)	0 (0.2)	1 (0.4)	3 (1.1)	3 (1.8)	12 (4.5)	19 (8.9)	401 (100)
Cefiderocol	52.7	47.3	0	3 (0.7)	2 (1.1)	17 (5.0)	57 (17.9)	153 (52.7)	154 (87.7)	26 (93.6)	8 (95.4)	3 (96.1)	17 (100)
Colistin	80.5^*d*^	19.5	0	5 (1.1)	241 (55.9)	90 (76.4)	12 (79.1)	6 (80.5)	3 (81.1)	20 (85.7)	63 (>8) (100)		
Ertapenem	1.1	98.9	3 (0.7)	0 (0.7)	1 (0.9)	1 (1.1)	4 (2.0)	9 (4.1)	3 (4.8)	10 (7.0)	409 (>8) (100)		
Tigecycline^*b*^	97.5^*d*^	0.2	2 (0.4)	27 (6.6)	91 (27.3)	109 (52.0)	145 (85.0)	55 (97.5)	10 (99.8)	1 (100)			
Tobramycin	10	90	0	1 (0.2)	12 (2.9)	19 (7.3)	9 (9.3)	3 (10)	13 (12.9)	55 (25.5)	328 (>8) (100)		
Minocycline^*c*^	36.8	39.3	0	0	2 (0.5)	6 (1.8)	37 (10.2)	59 (23.6)	58 (36.8)	105 (60.7)	75 (78.1)	98 (>16) (100)	

^*a*^ *Enterobacterales* included the following genera: *Escherichia* (11.1%), *Citrobacter* (0.9%), *Enterobacter* (9.1%), *Klebsiella* (74.3%), *Proteus* (0.9%), *Providencia* (2.3%), *Morganella* (0.2%), and *Serratia* (1.1%).

^*b*^ FDA breakpoints have been used.

^*c*^ CLSI breakpoints have been used.

^*d*^ Intrinsic resistance to colistin and tigecycline has been noted in *Proteus *spp.*, Providencia *spp*., M. morganii*, and *S. marcescens *and may contribute to elevated MIC values.

^*e*^ EUCAST, European Committee on Antimicrobial Susceptibility Testing; MBL, metallo-β-lactamase; MIC, minimum inhibitory concentration; S, susceptibility; R, resistance.

ATM-AVI inhibited 96.8% of isolates carrying NDM genotype (MIC_50/90_, 0.25/1 mg/L) (Table 7) at a concentration of ≤4 mg/L. Among comparator agents, tigecycline showed potent activity at 97.5%, and colistin was active at 81.3%, while cefiderocol exhibited moderate activity at 51.1% against NDM-carrying isolates. ATM-AVI inhibited 75.0% (MIC_50/90_, 2/16 mg/L) of *E. coli* and 100% (MIC_50/90_, 0.25/0.5 mg/L) of *K. pneumoniae* isolates carrying NDM at a concentration of ≤4 mg/L. Tigecycline and colistin displayed complete activity (100%) to *E. coli* isolates carrying NDM, that showed high resistance to cefiderocol at 64.6%. Tigecycline (98.3%) and colistin (81.4%) maintained high activity, and cefiderocol was active against 55.0% of *K. pneumoniae* isolates carrying NDM (Table S17). ATM-AVI inhibited 100% of NDM-carrying *Enterobacterales* from the AfME, Europe, and Latin America regions at a concentration of ≤4 mg/L, while 91.9% (MIC_50/90_, 0.5/4 mg/L) of isolates were inhibited from the APAC region (Table S18). ATM-AVI also displayed robust activity against NDM-1 (99.0%; MIC_50/90_, 0.25/0.5 mg/L) and NDM-5 (93.7%; MIC_50/90_, 0.5/4 mg/L) positive *Enterobacterales* at an MIC of ≤4 mg/L. ATM-AVI maintained complete activity against NDM-5-positive *K. pneumoniae* (*N* = 126), whereas 73.2% of NDM-5-positive *E. coli* (*N* = 30/41) were inhibited by ATM-AVI at a concentration of ≤4 mg/L. Tigecycline retained high activity for both NDM-1 (96.0%) and NDM-5 (98.8%), whereas cefiderocol showed moderate activity (NDM-1 at 49.8% and NDM-5 at 54.6%) (Tables S15 and S16). ATM-AVI achieved complete activity (100%) against isolates carrying VIM (MIC_50/90_, 0.5/2 mg/L) (Table 8) and IMP (MIC_50/90_, 0.25/0.5 mg/L) (Table 9) genotypes at a concentration of ≤4 mg/L. Colistin (58.3%) and cefiderocol (66.7%) showed moderate activity against VIM-carrying isolates, while tigecycline retained high activity (95.8%). Tigecycline and colistin displayed complete activity (100%) against IMP-carrying isolates, whereas cefiderocol showed moderate activity at 72.7%.

**TABLE 7:** Antimicrobial activity of aztreonam-avibactam and comparators against NDM variants of MBL-positive *Enterobacterales* isolated from BSI, 2021–2022^*e*^

Antimicrobial agent	Susceptibility (EUCAST)		No. of isolates inhibited at respective MIC (mg/L) (% cumulative inhibition)										
	%S	%R	≤0.06	0.12	0.25	0.5	1	2	4	8	16	32	>32
NDM (*N* = 407)**^*a*^**													
Aztreonam/avibactam	96.8	3.2	74 (18.2)	66 (34.4)	103 (59.7)	99 (84.0)	32 (91.9)	8 (93.9)	12 (96.8)	7 (98.5)	4 (99.5)	0 (99.5)	2 (100)
Aztreonam	10.1	87.5	12 (2.9)	3 (3.7)	12 (6.6)	6 (8.1)	8 (10.1)	5 (11.3)	5 (12.5)	6 (14.0)	5 (15.2)	27 (21.9)	318 (100)
Cefepime	0.2	99.5	0	1 (0.2)	0 (0.2)	0 (0.2)	0 (0.2)	1 (0.5)	0 (0.5)	2 (1.0)	6 (2.5)	15 (6.1)	382 (100)
Cefiderocol	51.1	48.9	0	3 (0.7)	2 (1.2)	7 (3.0)	49 (15.0)	147 (51.1)	146 (87.0)	26 (93.4)	7 (95.1)	3 (95.8)	17 (100)
Colistin	81.3^*d*^	18.7	0	5 (1.2)	225 (56.5)	83 (76.9)	12 (79.9)	6 (81.3)	3 (82.1)	20 (87.0)	53 (>8) (100)		
Ertapenem	0.7	99.3	2 (0.5)	0 (0.5)	1 (0.7)	0 (0.7)	1 (1.0)	0 (1.0)	1 (1.2)	7 (3.0)	395 (>8) (100)		
Minocycline^*b*^	34.9	39.8	0	0	2 (0.5)	6 (2.0)	33 (10.1)	53 (23.1)	48 (34.9)	103 (60.2)	74 (78.4)	88 (>16) (100)	
Tigecycline^*c*^	97.5^*d*^	0	2 (0.5)	25 (6.6)	86 (27.8)	103 (53.1)	132 (85.5)	49 (97.5)	10 (100)				
Tobramycin	10.1	89.9	0	1 (0.3)	12 (3.2)	18 (7.6)	7 (9.3)	3 (10.1)	11 (12.8)	50 (25.1)	305 (>8) (100)		

^*a*^ *Enterobacterales* included the following genera: *Escherichia* (11.8%), *Citrobacter* (1%), *Enterobacter* (7.4%), *Klebsiella* (75.4%), *Proteus* (1%), *Providencia* (2.5%), *Morganella* (0.2%), and *Serratia* (0.7%).

^*b*^ CLSI breakpoints have been used.

^*c*^ FDA breakpoints have been used.

^*d*^ Intrinsic resistance to colistin and tigecycline has been noted in *Proteus *spp.*, Providencia *spp*., M. morganii*, and *S. marcescens *and may contribute to elevated MIC values.

^*e*^ EUCAST, European Committee on Antimicrobial Susceptibility Testing; MIC, minimum inhibitory concentration; S, susceptibility; R, resistance.

**TABLE 8:** Antimicrobial activity of aztreonam-avibactam and comparators against VIM variants of MBL-positive *Enterobacterales* isolated from BSI, 2021–2022^*e*^

Antimicrobial agent	Susceptibility (EUCAST)		No. of isolates inhibited at respective MIC (mg/L) (% cumulative inhibition)										
	%S	%R	≤0.06	0.12	0.25	0.5	1	2	4	8	16	32	>32
VIM (*N* = 24)**^*a*^**													
Aztreonam/avibactam	100	0	1 (4.2)	5 (25.0)	2 (33.3)	12 (83.3)	1 (87.5)	3 (100)					
Aztreonam	4.2	91.7	0	0	1 (4.2)	0 (4.2)	0 (4.2)	0 (4.2)	1 (8.3)	3 (20.8)	1 (25.0)	2 (33.3)	16 (100)
Cefepime	0	95.8	0	0	0	0	0	0	1 (4.2)	1 (8.3)	3 (20.8)	0 (20.8)	19 (100)
Cefiderocol	66.7	33.3	0	0	0	5 (20.8)	5 (41.7)	6 (66.7)	5 (87.5)	1 (91.7)	1 (95.8)	1 (100)	
Colistin	58.3^*d*^	41.7	0	10 (41.7)	4 (58.3)	0 (58.3)	0 (58.3)	0 (58.3)	0 (58.3)	0 (58.3)	10 (>8) (100)		
Ertapenem	8.3	91.7	1 (4.2)	0 (4.2)	0 (4.2)	1 (8.3)	0 (4.1)	4 (25.0)	0 (25.0)	2 (33.3)	16 (>8) (100)		
Minocycline^*b*^	66.7	25	0	0	0	0	1 (4.2)	6 (29.2)	9 (66.7)	2 (75.0)	1 (79.2)	5 (>16) (100)	
Tigecycline^*c*^	95.8^*d*^	4.2	0	0	4 (16.7)	3 (29.2)	12 (79.2)	4 (95.8)	0 (95.8)	1 (100)			
Tobramycin	8.3	91.7	0	0	0	0	2 (8.3)	0 (8.3)	2 (16.7)	4 (33.3)	16 (>8) (100)		

^*a*^ *Enterobacterales* included the following genera: *Escherichia* (0%), *Citrobacter* (0%), *Enterobacter* (25%), *Klebsiella* (66.7%), *Proteus* (0%), *Providencia* (0%), *Morganella* (0%), and *Serratia* (8.3%).

^*b*^ CLSI breakpoints have been used.

^*c*^ FDA breakpoints have been used.

^*d*^ Intrinsic resistance to colistin and tigecycline has been noted in *Proteus *spp.*, Providencia *spp*., M. morganii*, and *S. marcescens *and may contribute to elevated MIC values.

^*e*^ EUCAST, European Committee on Antimicrobial Susceptibility Testing; MIC, minimum inhibitory concentration; S, susceptibility; R, resistance.

**TABLE 9:** Antimicrobial activity of aztreonam-avibactam and comparators against IMP variants of MBL-positive *Enterobacterales* isolated from BSI, 2021–2022^*e*^

Antimicrobial agent	Susceptibility (EUCAST)		No. of isolates inhibited at respective MIC (mg/L) (% cumulative inhibition)										
	%S	%R	≤0.06	0.12	0.25	0.5	1	2	4	8	16	32	>32
IMP (*N* = 11)**^*a*^**													
Aztreonam/avibactam	100	0	3 (27.2)	2 (45.4)	2 (63.6)	3 (90.9)	1 (100)						
Aztreonam	54.5	36.4	1 (9.1)	2 (27.2)	2 (45.4)	1 (54.5)	0 (54.5)	1 (63.6)	0 (63.6)	0 (63.6)	0 (63.6)	1 (72.7)	3 (100)
Cefepime	0	81.8	0	0	0	0	0	0	2 (18.1)	0 (18.1)	3 (45.4)	4 (81.8)	2 (100)
Cefiderocol	72.7	27.3	0	0	0	5 (45.4)	3 (72.7)	0 (72.7)	3 (100)				
Colistin	100^*d*^	0	0	0	8 (72.7)	3 (100)							
Ertapenem	0	100	0	0	0	0	3 (27.2)	5 (72.7)	2 (90.9)	1 (100)			
Minocycline^*b*^	54.5	45.5	0	0	0	0	3 (27.3)	2 (45.5)	1 (54.5)	0 (54.5)	0 (54.5)	5 (>16) (100)	
Tigecycline^*c*^	100^*d*^	0	0	2 (18.2)	3 (45.4)	3 (72.7)	1 (81.8)	2 (100)					
Tobramycin	9.1	90.9	0	0	0	1 (9.1)	0	0	0	1 (18.2)	9 (>8) (100)		

^*a*^ *Enterobacterales* included the following genera: *Escherichia* (9.1%), *Citrobacter* (0%), *Enterobacter* (54.5%), *Klebsiella* (36.4%), *Proteus* (0%), *Providencia* (0%), *Morganella* (0%), and *Serratia* (0%).

^*b*^ CLSI breakpoints have been used.

^*c*^ FDA breakpoints have been used.

^*d*^ Intrinsic resistance to colistin and tigecycline has been noted in *Proteus *spp.*, Providencia *spp*., M. morganii*, and *S. marcescens *and may contribute to elevated MIC values.

^*e*^ EUCAST, European Committee on Antimicrobial Susceptibility Testing; MIC, minimum inhibitory concentration; S, susceptibility; R, resistance.

ATM-AVI demonstrated complete activity (100%) against isolates co-harboring both MBL and KPC (MIC_50/90_, 0.5/1 mg/L) and it inhibited 98.2% of isolates carrying MBL in combination with OXA-48 and OXA-48-like genes (MIC_50/90_, 0.5/1 mg/L). ATM-AVI inhibited 97.8% of isolates co-harboring MBL and ESBL (MIC_50/90_, 0.25/1 mg/L), while 78.7% of isolates co-carrying MBL and AmpC were susceptible to ATM-AVI (MIC_50/90_, 1/16 mg/L) at a concentration of ≤4 mg/L. ATM-AVI effectively inhibited all *K. pneumoniae* isolates co-harboring MBL and AmpC (*N* = 26), while 41.2% of *E. coli* isolates co-harboring MBL and AmpC (*N* = 7/17) were susceptible to ATM-AVI at an MIC of ≤4 mg/L. Tigecycline showed potent activity (>90%) for all categories, while colistin maintained activity against MBLs co-carrying OXA-48 and OXA-48-like (86.2%), AmpC (89.4%), and ESBL (82.1%). Cefiderocol demonstrated moderate activity against MBLs co-carrying OXA-48 and OXA-48-like (61.5%), KPC (45.5%), and ESBL (54.3%), but had low activity against MBLs co-carrying AmpC isolates, with a resistance rate of 70.2% (Tables 10 to 13).

**TABLE 10:** Antimicrobial activity of aztreonam-avibactam and comparators against MBL-positive *Enterobacterales* co-carrying OXA-48 and OXA-48-like genes isolated from BSI, 2021–2022^*f*^

Antimicrobial agent	Susceptibility (EUCAST)		No. of isolates inhibited at respective MIC (mg/L) (% cumulative inhibition)										
	%S	%R	≤0.06	0.12	0.25	0.5	1	2	4	8	16	32	>32
MBL+OXA-48 and OXA-48-like^*a*^ (*N* = 109)^*b*^													
Aztreonam/avibactam	98.2	1.8	4 (3.7)	8 (11.0)	31 (39.4)	49 (83.4)	13 (96.3)	0 (96.3)	2 (98.2)	1 (99.1)	0 (99.1)	0 (99.1)	1 (100)
Aztreonam	8.3	90.8	1 (0.9)	1 (1.8)	1 (2.8)	3 (5.5)	3 (8.3)	0 (8.3)	1 (9.2)	0 (9.2)	0 (9.2)	1 (10.1)	98 (100)
Cefepime	0.9	99.1	0	1 (0.9)	0 (0.9)	0 (0.9)	0 (0.9)	0 (0.9)	0 (0.9)	1 (1.8)	1 (2.8)	0 (2.8)	106 (100)
Cefiderocol	61.5	38.5	0	0	1 (0.9)	0 (0.9)	9 (9.2)	57 (61.5)	36 (94.5)	2 (96.3)	0 (96.3)	0 (96.3)	4 (100)
Colistin	86.2^*e*^	13.8	0	0	71 (65.1)	22 (85.3)	0 (85.3)	1 (86.2)	0 (86.2)	4 (89.9)	11 (>8) (100)		
Ertapenem	1.8	98.2	1 (0.9)	0 (0.9)	1 (1.8)	0 (1.8)	0 (1.8)	0 (1.8)	0 (1.8)	2 (3.7)	105 (>8) (100)		
Minocycline^*c*^	24.8	43.1	0	0	0	2 (1.8)	6 (7.3)	8 (14.7)	11 (24.8)	35 (56.9)	19 (74.3)	28 (>16) (100)	
Tigecycline^*d*^	100^*e*^	0	0	3 (2.8)	15 (16.5)	23 (37.6)	45 (78.9)	23 (100)					
Tobramycin	7.3	92.7	0	0	4 (3.7)	4 (7.3)	0 (7.3)	0 (7.3)	1 (8.3)	9 (16.5)	91 (>8) (100)		

^*a*^ OXA-48-like constitutes OXA-163, OXA-181, OXA-232, and OXA-244.

^*b*^ *Enterobacterales* included the following genera: *Escherichia* (4.6%), *Citrobacter* (0.9%), *Enterobacter* (1.8%), *Klebsiella* (90.8%), *Proteus* (0%), *Providencia* (1.8%), *Morganella* (0%), and *Serratia* (0%).

^*c*^ CLSI breakpoints have been used.

^*d*^ FDA breakpoints have been used.

^*e*^ Intrinsic resistance to colistin and tigecycline has been noted in *Proteus *spp.*, Providencia *spp*., M. morganii*, and *S. marcescens *and may contribute to elevated MIC values.

^*f*^ EUCAST, European Committee on Antimicrobial Susceptibility Testing; MBL, metallo-β-lactamase; MIC, minimum inhibitory concentration; S, susceptibility; R, resistance.

**TABLE 11:** Antimicrobial activity of aztreonam-avibactam and comparators against MBL-positive *Enterobacterales* co-carrying KPC genes isolated from BSI, 2021–2022^*e*^

Antimicrobial agent	Susceptibility (EUCAST)		No. of isolates inhibited at respective MIC (mg/L) (% cumulative inhibition)										
	%S	%R	≤0.06	0.12	0.25	0.5	1	2	4	8	16	32	>32
MBL+KPC (*N* = 22)**^*a*^**													
Aztreonam/avibactam	100	0	2 (9.0)	1 (13.6)	2 (22.7)	14 (86.3)	3 (100)						
Aztreonam	0	100	0	0	0	0	0	0	0	1 (4.5)	1 (9.0)	0 (9.0)	20 (100)
Cefepime	0	100	0	0	0	0	0	0	0	0	0	0	22 (100)
Cefiderocol	45.5	54.5	0	0	0	4 (18.1)	1 (22.7)	5 (45.4)	8 (81.8)	3 (95.4)	0 (95.4)	1 (100)	0
Colistin	68.2^*d*^	31.8	0	0	10 (45.4)	4 (63.6)	1 (68.1)	0 (68.1)	0 (68.1)	0 (68.1)	7 (>8) (100)		
Ertapenem	0	100	0	0	0	0	0	0	0	0	22 (>8) (100)		
Minocycline^*b*^	45.5	45.5	0	0	0	0	2 (9.0)	2 (18.1)	6 (45.4)	2 (54.5)	3 (68.1)	7 (>16) (100)	
Tigecycline^*c*^	100^*d*^	0	1 (4.5)	0 (4.5)	5 (27.2)	3 (40.9)	9 (81.8)	4 (100)					
Tobramycin	4.5	95.5	0	0	0	1 (4.5)	0 (4.5)	0 (4.5)	0 (4.5)	4 (22.7)	17 (>8) (100)		

^*a*^ *Enterobacterales* included the following genera: *Escherichia* (0%), *Citrobacter* (0%), *Enterobacter* (9.1%), *Klebsiella* (86.4%), *Proteus* (0%), *Providencia* (0%), *Morganella* (4.5%), and *Serratia* (0%).

^*b*^ CLSI breakpoints have been used.

^*c*^ FDA breakpoints have been used.

^*d*^ Intrinsic resistance to colistin and tigecycline has been noted in *Proteus *spp.*, Providencia *spp*., M. morganii*, and *S. marcescens *and may contribute to elevated MIC values.

^*e*^ EUCAST, European Committee on Antimicrobial Susceptibility Testing; MBL, metallo-β-lactamase; KPC, *Klebsiella pneumoniae* carbapenemase; MIC, minimum inhibitory concentration; S, susceptibility; R, resistance.

**TABLE 12:** Antimicrobial activity of aztreonam-avibactam and comparators against MBL-positive *Enterobacterales* co-carrying acquired AmpC genes isolated from BSI, 2021–2022^*e*^

Antimicrobial agent	Susceptibility (EUCAST)		No. of isolates inhibited at respective MIC (mg/L) (% cumulative inhibition)										
	%S	%R	≤0.06	0.12	0.25	0.5	1	2	4	8	16	32	>32
MBL+AmpC (*N* = 47)**^*a*^**													
Aztreonam/avibactam	78.7	21.3	5 (10.6)	3 (17.0)	7 (31.9)	7 (46.8)	7 (61.7)	4 (70.2)	4 (78.7)	5 (89.4)	4 (97.9)	0 (97.9)	1 (100)
Aztreonam	2.1	93.6	0	0	0	1 (2.1)	0 (2.1)	0 (2.1)	2 (6.4)	2 (10.6)	0 (10.6)	12 (36.2)	30 (100)
Cefepime	0	100	0	0	0	0	0	0	0	0	3 (6.4)	5 (17.0)	39 (100)
Cefiderocol	29.8	70.2	0	0	0	1 (2.1)	1 (4.3)	12 (29.8)	17 (66.0)	4 (74.5)	3 (80.8)	0 (80.8)	9 (100)
Colistin	89.4^*d*^	10.6	0	2 (4.3)	29 (66.0)	8 (83.0)	3 (89.4)	0 (89.4)	1 (91.4)	0 (91.4)	4 (>8) (100)		
Ertapenem	0	100	0	0	0	0	0	1 (2.1)	0 (2.1)	0 (2.1)	46 (>8) (100)		
Minocycline^*b*^	46.8	38.3	0	0	0	2 (4.3)	7 (19.1)	5 (29.8)	8 (46.8)	7 (61.7)	10 (83.0)	8 (>16) (100)	
Tigecycline^*c*^	91.5^*d*^	2.1	1 (2.1)	10 (23.4)	13 (51.1)	9 (70.2)	9 (89.4)	1 (91.5)	3 (97.9)	1 (100)			
Tobramycin	23.4	76.6	0	0	3 (6.4)	4 (14.9)	4 (23.4)	0 (23.4)	1 (25.5)	3 (31.9)	32 (>8) (100)		

^*a*^ Includes *Escherichia coli* (36.2%),* Klebsiella oxytoca* (4.3%),* Klebsiella pneumoniae* (55.3%),* Klebsiella variicola* (0%), and* Proteus mirabilis* (4.3%).

^*b*^ CLSI breakpoints have been used.

^*c*^ FDA breakpoints have been used.

^*d*^ Intrinsic resistance to colistin and tigecycline has been noted in *Proteus *spp.*, Providencia *spp*., M. morganii*, and *S. marcescens *and may contribute to elevated MIC values.

^*e*^ EUCAST, European Committee on Antimicrobial Susceptibility Testing; MBL, metallo-β-lactamase; MIC, minimum inhibitory concentration; S, susceptibility; R, resistance.

**TABLE 13:** Antimicrobial activity of aztreonam-avibactam and comparators against MBL-positive *Enterobacterales* co-carrying ESBL genes isolated from BSI, 2021–2022^*e*^

Antimicrobial agent	Susceptibility (EUCAST)		No. of isolates inhibited at respective MIC (mg/L) (% cumulative inhibition)										
	%S	%R	≤0.06	0.12	0.25	0.5	1	2	4	8	16	32	>32
MBL+ESBL (*N* = 357)**^*a*^**													
Aztreonam/avibactam	97.8	2.2	46 (12.8)	57 (28.8)	99 (56.5)	102 (85.1)	27 (92.7)	7 (94.6)	11 (97.8)	5 (99.1)	2 (99.7)	0 (99.7)	1 (100)
Aztreonam	7.3	90.8	6 (1.6)	2 (2.2)	9 (4.7)	5 (6.1)	4 (7.2)	4 (8.4)	3 (9.2)	3 (10.0)	3 (10.9)	17 (15.6)	301 (100)
Cefepime	0.3	99.7	0	1 (0.2)	0 (0.2)	0 (0.2)	0 (0.2)	0 (0.2)	0 (0.2)	3 (1.1)	4 (2.2)	13 (5.8)	336 (100)
Cefiderocol	54.3	45.7	0	0	1 (0.2)	13 (3.9)	40 (15.1)	140 (54.3)	129 (90.4)	19 (95.8)	4 (96.9)	0 (96.9)	11 (100)
Colistin	82.1^*d*^	17.9	0	4 (1.1)	203 (57.9)	71 (77.8)	10 (80.6)	5 (82.0)	3 (82.9)	17 (87.6)	44 (>8) (100)		
Ertapenem	0.8	99.2	1 (0.2)	0 (0.2)	1 (0.5)	1 (0.8)	0 (0.8)	2 (1.4)	2 (1.9)	3 (2.8)	347 (>8) (100)		
Minocycline^*b*^	34.2	40.9	0	0	2 (0.5)	6 (2.2)	28 (10.0)	38 (20.7)	48 (34.1)	89 (59.1)	67 (77.8)	79 (>16) (100)	
Tigecycline^*c*^	97.8^*d*^	0.3	1 (0.2)	22 (6.4)	67 (25.2)	91 (50.7)	127 (86.2)	41 (97.7)	7 (99.7)	1 (100)			
Tobramycin	8.4	91.6	0	0	9 (2.5)	12 (5.8)	6 (7.5)	3 (8.4)	6 (10.0)	46 (22.9)	275 (>8) (100)		

^*a*^ Includes *Escherichia coli* (11.8%),* Klebsiella oxytoca* (1.1%),* Klebsiella pneumoniae* (85.4%),* Klebsiella variicola* (1.1%), and* Proteus mirabilis* (0.6%).

^*b*^ CLSI breakpoints have been used.

^*c*^ FDA breakpoints have been used.

^*d*^ Intrinsic resistance to colistin and tigecycline has been noted in *Proteus *spp.*, Providencia *spp*., M. morganii*, and *S. marcescens *and may contribute to elevated MIC values.

^*e*^ EUCAST, European Committee on Antimicrobial Susceptibility Testing; ESBL, extended-spectrum β-lactamase; MBL, metallo-β-lactamase; MIC, minimum inhibitory concentration; S, susceptibility; R, resistance.

## DISCUSSION

The growing prevalence of BSIs caused by MDR *Enterobacterales* poses a significant therapeutic challenge. In this global ATLAS study, ATM-AVI demonstrated potent *in vitro* activity against *Enterobacterales*, including MDR isolates producing diverse clinically relevant β-lactamases, alone or in combination. ATM-AVI showed potent *in vitro* activity against 99.8% of Enterobacterales overall and 98.9% of CRE at the susceptibility breakpoint of ≤4 mg/L, consistent with prior global reports (10, 17, 18, 29, 30). Among MBL-positive CRE bloodstream isolates, ATM-AVI retained strong activity, inhibiting 97.0% overall, with 96% susceptibility across key genotypes, including NDM (96.8%), IMP (100%), and VIM (100%). This activity reflects the complementary mechanism of ATM-AVI, combining aztreonam’s inherent stability to MBLs and avibactam’s ability to neutralize ESBL, KPC, OXA-48, and OXA-48-like, and AmpC β-lactamases, thereby restoring ATM’s activity against MBL-producing (31). These findings highlight a clear advantage over comparator agents, including cefiderocol, which exhibited activity in only 52.7% of MBL-producing isolates. Furthermore, our study confirms that NDM-enzymes are the most prevalent MBLs globally, with NDM-5 gaining prominence, particularly in Asia, while VIM and IMP remain comparatively rare.

Despite overall high activity, some variation was noted. In APAC, susceptibility among NDM-positive isolates decreased to 91.9% (MIC90: 4 mg/L), driven by lower activity against NDM-5 (93.7%) compared to NDM-1 (99.0%). Species-specific differences were also evident: while ATM-AVI retained full activity against NDM-positive *K. pneumoniae*, reduced susceptibility was observed in *E. coli* (75.0% susceptible; MIC90: 16 mg/L), particularly among 10 isolates from India co-carrying NDM-5 and CMY AmpC variants. Overall, MBL-positive isolates co-carrying AmpC enzymes demonstrated reduced susceptibility, with 78.7% susceptible to ATM-AVI at a concentration of ≤4 mg/L, with an elevated MIC90 value of 16 mg/L. Previous studies have linked reduced ATM-AVI susceptibility in *E. coli* to PBP3 insertions and CMY enzymes (32–34). Periasamy et al. (33) observed raised ATM-AVI MICs (2–32 mg/L) in 23% of *E. coli* isolates, attributing this to PBP3 insertions in NDM + *E. coli* (33). Tellapragada et al. (35) reported PBP3 inserts (YRIK/YRIN) in all 20 resistant *E. coli* isolates studied, with CMY-42 present in five. They identified several mutations in ftsI (encoding PBP3) and regulatory genes of outer membrane proteins and efflux pumps, concluding that resistance to ATM-AVI results from a complex interplay of target alterations, deactivating enzymes, and decreased permeability (35). Although this study did not assess PBP3 insertion mutations, the elevated MICs observed in the APAC region may reflect similar resistance mechanisms. Further study of the prevalence of non-enzymatic resistance mechanisms to ATM-AVI is warranted, given the challenges in their diagnostic identification.

Consistent with global epidemiology, NDM was the predominant genotype among the 650 CP-CRE isolates (50.8%, 330/650), followed by OXA-48 and OXA-48-like variants (34.2%, 222/650) and KPC (32.5%, 211/650). These findings align with recent analysis observing NDM as the most frequent carbapenemase at 52.2%, followed by OXA-48 and OXA-48-like at 30.1% and KPC at 14.7% among CP-*E. coli* (36). Marked regional variability was observed. NDM predominated in APAC (76.3%, 132/173) and AfME (73.8%, 76/103), whereas KPC was more common in Europe (50.8%, 101/199) and LATAM (61.1%, 107/175), and was largely absent in AfME and APAC. These patterns align with prior reports, including high NDM prevalence in Asia (82.6%) compared with Europe (12.9%), and co-dominance of NDM (43.1%) and OXA-48 and OXA-48-like (42.9%) enzymes in Africa (14, 37). Country-level studies further support this heterogeneity. In a Chinese study, carbapenemase genes were detected in 81.8% of CRE-BSI isolates, with NDM present in 66.7% (36/54) and KPC in 29.6% (16/54) (38). In Argentina, the prevalence of MBL (44.2%) and KPC (36.1%) was more comparable (39). In contrast, data from Italy and Greece reported a predominance of NDM (80.4%), followed by VIM (19.6%), with OXA-type enzymes being far less common (40). Within APAC, a significant proportion of OXA-48 and OXA-48-like enzymes was also observed (63.6%, 110/173). This is broadly consistent with prior reports of OXA-48 and OXA-48-like enzymes as most common in India and South Africa, but rare in China (15). Notably, high rates of co-carriage have been described in Thailand (51.7% with NDM-1 plus OXA-48) (41). Overall, these findings reinforce the well-established geographic heterogeneity of CP-CRE epidemiology and emphasize the importance of regional and local surveillance to inform treatment decisions.

The current study confirms NDM as the most prevalent genotype among 440 MBL-positive *Enterobacterales* isolates, representing 92.5% (407/440) of the total, while VIM and IMP genotypes are notably less common at 5.5% (24/440) and 2.5% (11/440), respectively. Regionally, NDM-1 was predominantly found in Europe (71.4%, 50/70), while NDM-5 was more common in the APAC region (58.0%, 98/169), consistent with those reported by Kanj et al. (12). Similar geographic patterns have been reported for IMP and VIM by Kanj et al. (12). India accounted for 25.7% of all MBL isolates, with 76.0% (86/113) identified as NDM-5 and 19.5% (22/113) as NDM-1, aligning with recent data indicating a shift from NDM-1 to NDM-5 (9, 42, 43). In the Chinese mainland, 93.6% (15/16) of MBL isolates were NDMs, evenly split between NDM-1 (*N* = 14) and NDM-5 (*N* = 7). These trends reinforce the dominant role of NDM genotypes in both India and China and reflect broader regional patterns of carbapenemase prevalence in bloodstream infections.

In this study, cefiderocol showed moderate activity against key MBL variants, including NDM (51.1%; NDM-1 at 49.5% and NDM-5 at 54.6%), IMP (72.7%), and VIM (66.7%). Activity was further reduced in isolates co-harboring beta-lactamases, including MBL plus KPC (45.5% susceptible), and was especially limited in isolates with combined MBL and AmpC enzymes, where 70.2% were resistant. Among species, cefiderocol inhibited 55.0% of NDM-positive *K. pneumoniae* but showed limited activity against NDM-positive *E. coli* (35.4%). In comparison, Sader et al. (44) reported higher overall cefiderocol activity among MBLs (63.5% overall) in Europe (44). Consistent with these findings, a systematic review reported 42%–59% nonsusceptibility to cefiderocol in certain cohorts among NDM, KPC, and AmpC variants conferring resistance to ceftazidime-avibactam (45). Additionally, surveillance studies have shown higher cefiderocol MIC values for NDM-positive isolates compared to VIM-positive isolates (46). Collectively, these data highlight the variable activity of cefiderocol across genetic variants, with NDM-positive isolates.

It is crucial to consider the study’s limitations when interpreting the findings. The predetermined number of isolates collected per participating center may limit the generalizability of the findings to broader populations. In addition, sample sizes were lower for some resistant phenotypes and regions, which should be taken into account when interpreting the findings. ESBL screening was based only on ceftazidime and aztreonam MICs, as cefotaxime was not included in the routine ATLAS surveillance protocol. The absence of clavulanate-based confirmatory testing meant the isolates meeting screening criteria were classified as ESBL screen-positive rather than confirmed ESBL-positive. This approach may have reduced detection of some CTX-M-type ESBLs, particularly in regions where these enzymes predominate. Another limitation relates to the variability in data sources for MBL-positive isolates. Molecular characterization data for CRE isolates from the Chinese mainland were not available within the core surveillance program; however, these data were obtained as an ancillary project and incorporated to enhance representation in the APAC region. Differences in data processing across these sources may introduce inconsistencies. Finally, this study included isolates collected through 2022; more recent data were not available due to susceptibility testing and molecular characterization occurring in the year following collection per the ATLAS data refresh cycle.

### Conclusion

This global ATLAS study underscores the substantial and geographically variable burden of MBL-producing Enterobacterales, driven predominantly by NDM variants. ATM-AVI demonstrated consistently robust *in vitro* activity against *Enterobacterales* producing diverse β-lactamases, alone or in combination, in bloodstream infections. ATM-AVI exhibited remarkable activity, inhibiting over 99% of *Enterobacterales* isolates across all regions at an MIC of ≤4 mg/L and outperforming comparator agents. It consistently maintained robust activity across various resistant phenotypes, including MDR and CRE, as well as against ESBL screen-positive and MBL-positive *Enterobacterales*. The ability of ATM-AVI to treat the co-production of multiple enzymes in a single isolate underscores its comprehensive β-lactamase coverage, which is unique among currently available agents. By addressing a critical gap in the current antibiotic arsenal, ATM-AVI offers a promising option for the management of severe infections, including BSIs. Continuous monitoring of resistance patterns will be essential to guide and optimize its use across diverse geographical settings.

## Supplementary Material

Reviewer comments

## Data Availability

The data sets generated during and/or analyzed during the current study are available from the corresponding author on reasonable request.

## SUPPLEMENTAL MATERIAL

Supplemental tables spectrum.00241-26-s0001.docx Tables S1 to S18.

OPEN PEER REVIEW reviewer-comments.pdf An accounting of the reviewer comments and feedback.

ASM does not own the copyrights to Supplemental Material that may be linked to, or accessed through, an article. The authors have granted ASM a non-exclusive, world-wide license to publish the Supplemental Material files. Please contact the corresponding author directly for reuse.
