# Supplementary material for: In vitro activity of aztreonam-avibactam and comparators against Enterobacterales isolated from bloodstream infections: ATLAS/ surveillance, 2021–2022
Source: Microbiol Spectr. 2026 Jun 30;14(8):e00241-26. doi: 10.1128/spectrum.00241-26 (PMC13436376; doi:10.1128/spectrum.00241-26)
Supplement: Supplementary file 1 [file reviewer-comments.pdf]

Microbiology Spectrum

In vitro activity of aztreonam-avibactam and comparators against Enterobacterales isolated from bloodstream infections: ATLAS surveillance, 2021-2022

Gian Maria Rossolini, Fupin Hu, Nandini Sethuraman, Shweta Kamat, Naglaa Mohamed, Gregory Stone, and Katherine Perez

Corresponding Author(s): Katherine Perez, Pfizer Inc

Review Timeline:

Submission Date:	January 20, 2026
Editorial Decision:	April 9, 2026
Revision Received:	June 2, 2026
Accepted:	June 4, 2026

Editor: Krisztina Papp-Wallace

Reviewer(s): The reviewers have opted to remain anonymous.

Transaction Report:

(Note: With the exception of the correction of typographical or spelling errors that could be a source of ambiguity, letters and reports are not edited. The original formatting of letters and referee reports may not be reflected in this compilation.)

DOI: https://doi.org/10.1128/spectrum.00241-26

Re: Spectrum00241-26 (In vitro activity of aztreonam-avibactam and comparators against Enterobacterales isolated from bloodstream infections: ATLAS surveillance, 2021-2022)

Dear Dr. Katherine K. Perez:

Thank you for the privilege of reviewing your work. Below you will find my comments, instructions from the Spectrum editorial office, and the reviewer comments.

Please return the manuscript within 60 days; if you cannot complete the modification within this time period, please contact me. If you do not wish to modify the manuscript and prefer to submit it to another journal, notify me immediately so that the manuscript may be formally withdrawn from consideration by Spectrum.

Revision Guidelines
To submit your modified manuscript, log into the submission site at https://spectrum.msubmit.net/cgi-bin/main.plex. Go to Author Tasks and click the appropriate manuscript title to begin. The information you entered when you first submitted the paper will be displayed; update this as necessary. Note the following requirements:

• Upload point-by-point responses to the issues raised by the reviewers in a file named "Response to Reviewers," NOT in your cover letter.
• Upload a compare copy of the manuscript (without figures) as a "Marked-Up Manuscript" file.
• Upload a clean .DOC/.DOCX version of the revised manuscript and remove the previous version.
• Each figure must be uploaded as a separate, editable, high-resolution file (TIFF or EPS preferred), and any multipanel figures must be assembled into one file.
• Any supplemental material intended for posting by ASM should be uploaded with their legends separate from the main manuscript. You can combine all supplemental material into one file (preferred) or split it into a maximum of 10 files with all associated legends included.

For complete guidelines on revision requirements, see our Submission and Review Process webpage. Submission of a paper that does not conform to guidelines may delay acceptance of your manuscript.

Data availability: ASM policy requires that data be available to the public upon online posting of the article, so please verify all links to sequence records, if present, and make sure that each number retrieves the full record of the data. If a new accession number is not linked or a link is broken, provide Spectrum production staff with the correct URL for the record. If the accession numbers for new data are not publicly accessible before the expected online posting of the article, publication may be delayed; please contact production staff (Spectrum@asmusa.org) immediately with the expected release date.

Publication Fees: For information on publication fees and which article types are subject to charges, visit our website. If your manuscript is accepted for publication and any fees apply, you will be contacted separately about payment during the production process; please follow the instructions in that e-mail. Arrangements for payment must be made before your article is published.

ASM Membership: Corresponding authors may join or renew ASM membership to obtain discounts on publication fees. Need to upgrade your membership level? Please contact Customer Service at Service@asmusa.org.

The ASM Journals program strives for constant improvement in our submission and publication process. Please tell us how we can improve your experience by taking this quick Author Survey.

Thank you for submitting your paper to Spectrum.

Sincerely,
Krisztina Papp-Wallace
Editor
Microbiology Spectrum

Reviewer #1 (Comments for the Author):

Lines 35, 37 and 41 - describing inhibition of isolates at an 'MIC of {less than or equal to} 4mg/L' is confusing, using the term 'concentration' or 'breakpoint' is more fitting here. An MIC value describes the readout of the test against an individual isolate, and would not be encompassing of the isolates which were not inhibited at this drug concentration (if broth microdiliution was the method used to measure inhibition). Readers unfamiliar with the test method may confuse this as isolates with ATM-AVI

susceptibility {less than or equal to} 4mg/L. The authors are using this in the way that you would describe MIC50/90, however 'breakpoint' is more commonly applied when discussing overall isolate susceptibility in this manner.

Line 22 - Why was cefotaxime not also applied as an ESBL screen? Ceftazidime has poorer capture of CTX-M type ESBL variants than ceftazidime, hence why CAZ and CTX in conjunction with ATM are both used in most ESBL screen tests. Some CTX type variants may have been missed using this method, especially in regions where they are more common than TEM/SHV ESBLs.

Line 111 - the caz/avibactam phenotypic selection in itself was technically a screen - it is not clear here how the authors defined isolates as ESBL positive, given that phenotypic confirmatory testing with inhibitors were not used. Does 'qualified' mean sent for downstream ESBL confirmation after having fulfilled the criteria of CAZ MIC {greater than or equal to} 2mg/L, or ATM MIC {greater than or equal to} 2mg/L? Or were isolates fulfilling either of these phenotypic criteria designated as ESBL? If detection of this phenotype was used to select isolates for further ESBL testing, elaborate on the follow-on confirmatory testing method used and how isolates were finally confirmed as 'ESBL positive'. If a CAZ MIC {greater than or equal to} 2mg/L, or ATM MIC {greater than or equal to} 2mg/L phenotype was used as a proxy for ESBL, the authors need to describe this as such, as this method alone is not sufficient to determine true ESBL status - pAmpC producers, or TEM over-expressors could also be selected using these inclusion criteria.

Lines 130 - 134 - This would be a very nice follow-on study, as understanding the prevalence of non-enzymatic resistance mechanisms to ATM-AVI would be of global clinical interest given the difficulty in identifying them diagnostically (especially in regions where routine molecular testing is not commonplace).

Reviewer #2 (Comments for the Author):

This study evaluated in vitro activity of aztreonam-avibactam (ATM-AVI) and comparators against 10,376 Enterobacterales bloodstream isolates collected across Africa and the Middle East, Asia-Pacific, Europe, and Latin America during2021-2022 . ATM-AVI demonstrated potent and consistent activity against Enterobacterales, including MDR, CRE, and MBL-positive phenotypes, and these data are of great importance for clinical doctors to select effectve antibiotics in clinical practice. I think the manuscript will be more readable if the authors can clarify a few questions in the study, which are listed in the manuscript as comment.

In vitro activity of aztreonam-avibactam and comparators against *Enterobacterales* isolated from bloodstream infections: ATLAS surveillance, 2021-2022

Running title: ATM-AVI activity against *Enterobacterales* from BSI

Gian Maria Rossolini[1], Fupin Hu[2,3,4], Nandini Sethuraman[5], Shweta Kamat[6], Naglaa Mohamed[7], Gregory Stone[7], Katherine K. Perez[7*]

[1]Department of Experimental and Clinical Medicine, University of Florence, and Microbiology and Virology Unit, Careggi University Hospital, 50134 Florence, Italy

[2]Institute of Antibiotics, Huashan Hospital, Fudan University, Shanghai, China

[3]Key Laboratory of Clinical Pharmacology of Antibiotics, Ministry of Health, Shanghai, China

[4]Joint Laboratory of Hospital & Enterprise for Pathogen Diagnosis of Drug-resistant Bacterial Infections and Innovative Drug R&D, Shanghai, China

[5]Department of Microbiology, Central Reference Laboratory, Apollo Hospitals, Chennai, India

[6]PPIPL, Mumbai, India

[7]Pfizer Inc., New York, NY

Corresponding Author

Katherine K. Perez

Senior Director, Infectious Diseases Surveillance Lead

Global Medical Affairs, Research & Development

Pfizer Inc., New York, NY

Email: katherine.perez@pfizer.com

ORCID: 0000-0002-7535-997X

Abstract

Carbapenem-resistant *Enterobacterales* (CRE) present a major global health concern due to their rapid spread, high mortality rates, and limited treatment options. metallo-β-lactamase (MBL)-producing CRE, particularly those harboring NDM variants and co-expressing beta-lactamases, are a significant therapeutic challenge as they exhibit resistance to most β-lactam/β-lactamase inhibitor combinations. This study evaluated *in vitro* activity of aztreonam-avibactam (ATM-AVI) and comparators against *Enterobacterales* bloodstream isolates (N = 10,376) collected between 2021 and 2022 across Africa and the Middle East, Asia-Pacific, Europe, and Latin America, as part of the ATLAS Global Surveillance Program. Minimum inhibitory concentrations (MIC) were determined by broth microdilution, with susceptibility interpreted primarily using 2025 EUCAST criteria as appropriate. ATM-AVI demonstrated robust antimicrobial activity, inhibiting 99.8% of *Enterobacterales* at MIC ≤4 mg/L and maintaining activity across various phenotypes: 99.6% for MDR, 98.9% for CRE and 99.9% for ESBL screen positive isolates. In MBL-positive CRE isolates, ATM-AVI inhibited 97% of isolates at MIC ≤4 mg/L, whereas comparators like cefiderocol inhibited 52.7% of isolates at the clinical breakpoint. ATM-AVI's activity remained robust against isolates co-carrying ESBLs (97.8%) and other carbapenemases, like KPC (100%) and OXA-48/OXA-48-like enzymes (98.2%). For MBL-positive isolates co-carrying AmpC-type β-lactamases, ATM-AVI showed 78.7% activity at MIC ≤4 mg/L. ATM-AVI demonstrated potent and consistent activity against *Enterobacterales*, including MDR, CRE, and MBL-positive phenotypes, across diverse regions. ATM-AVI's ability to overcome resistance mediated by multiple β-lactamases highlights its clinical potential for managing severe infections like BSIs and the ongoing need for global surveillance data to drive clinical decision making and regional stewardship programs.

Keywords: aztreonam-avibactam, *Enterobacterales*, MBL, NDM, CRE

Introduction

The rise of carbapenem-resistant *Enterobacterales* (CRE) is a major global health concern that has prompted both the World Health Organization (WHO) and Centers for Disease Control and Prevention (CDC) to identify CRE as critical and urgent priority pathogens for novel drug development [1-3]. Recently, the incidence of CRE bloodstream infections (BSI) across Europe has increased from 2.5 per 100,000 population in 2019 to 3.97 per 100,000 in 2023 [4]. The recent CDC report indicates that the annual unadjusted incidence of CRE in 2023 was 18% higher than in 2019 across 29 states, which collectively represent 35% of the United States population [5]. These infections are linked to high mortality rates (30% - 75%) and increased healthcare costs, primarily due to delays in effective treatment and limited therapeutic options [4, 6].

Resistance in CRE is most often mediated by carbapenemase enzymes of Ambler class A, B, and D, which are encoded by genes carried on mobile genetic elements [7, 8]. Key examples of these enzymes are *Klebsiella pneumoniae* carbapenemase (KPC) from class A, metallo-beta-lactamases (MBL) such as New Delhi (NDM), Verona integron-encoded (VIM), imipenemase (IMP) from class B, and oxacillinase-48 (OXA-48) from class D [8]. Among these, MBL-producing CRE pose a significant challenge due to their resistance to newly introduced β-lactam/β-lactamase (BL/BLI) combinations, including ceftazidime-avibactam, meropenem-vaborbactam, and imipenem-relebactam, in clinical settings [9]. MBL-positive CRE are associated with poor clinical outcomes, emphasizing the urgent need for effective treatment strategies targeting MBLs [10]. These pathogens often co-produce other beta-lactamases, such as extended-spectrum beta-lactamases (ESBL) and AmpC type enzymes, further complicating therapeutic approaches [11]. NDM-type CRE are the most prevalent globally, with a particularly high prevalence of 82.6% in Asia, largely attributed to significant contributions from India and China [12]. There have also been numerous reports indicating a rise in the detection of MBL genes and isolates harboring dual carbapenemase genes, including OXA-48 and NDM, across European countries [4]. The recent CDC report highlights a significant increase in the age-adjusted incidence of NDM-CRE in the US, with a 461% rise observed across 48% of states between 2019 and 2023 [5]. Factors such as travel, international trade, and healthcare exposure have facilitated the global spread of NDM-producing CRE, contributing to the changing epidemiology of carbapenemase-producing CRE (CP-CRE) [13].

Aztreonam-avibactam (ATM-AVI) is a novel BL/BLI combination, demonstrating efficacy against MBL-positive CRE by leveraging aztreonam's stability against MBLs and avibactam's activity as inhibitor of serine beta-lactamases [1, 14]. ATM-AVI has demonstrated potent *in vitro* activity against MBL-positive *Enterobacterales* [8, 15-17]. In a Phase 3 REVISIT trial, ATM-AVI achieved clinical cure rates comparable to meropenem ± colistin for complicated intra-abdominal infections and hospital-acquired pneumonia [18].

Additionally, in the ASSEMBLE Phase 3 trial, ATM-AVI ± metronidazole was effective in treating patients with infections caused by MBL-positive Gram-negative bacteria (GNB) [19]. These findings supported regulatory approval of ATM-AVI by the European Medicines Agency (EMA) in 2024 [20] and the United States Food and Drug Administration (US FDA) in 2025 [21].

This study evaluates the *in vitro* activity of ATM-AVI and comparators against *Enterobacterales*, including MBL-positive CRE, isolated from BSIs between 2021 and 2022 across Africa and the Middle East, Asia-Pacific, Europe, and Latin America, as part of the Antimicrobial Testing Leadership and Surveillance (ATLAS) Global Surveillance Program [22].

Methods

Bacterial isolates

Enterobacterales isolates (N = 10,376) were collected from hospitalized patients with BSIs between 2021 and 2022 as part of the ATLAS Program from the following regions: Africa/Middle East (AfME), Asia-Pacific (APAC), Europe, and Latin America [22]. The isolates represented diverse genera of *Enterobacterales* including *Escherichia spp*, *Citrobacter spp*, *Enterobacter spp*, *Klebsiella spp*, *Proteus spp*, *Serratia spp*, *Morganella spp*, and *Providencia spp*.

Each isolate was sent to a central reference laboratory (IHMA, Schaumburg, IL, USA) for confirmation and antimicrobial susceptibility testing; species identification was confirmed by MALDI-TOF mass spectrometry (Bruker Daltonics, Billerica, MA, USA).

Antimicrobial susceptibility testing

Minimum inhibitory concentrations (MICs) were determined by broth microdilution following CLSI guidelines [23]. ATM-AVI and a panel of comparator antimicrobial agents including aztreonam, cefepime, colistin, meropenem, tigecycline, amikacin, ceftazidime-avibactam, ceftolozane-tazobactam, and piperacillin-tazobactam were tested against *Enterobacterales* isolates (N = 10,376). For the ATLAS program years 2021 and 2022, cefiderocol, ertapenem, minocycline, and tobramycin were not part of the routine testing panel. Hence, the antimicrobial activity of ATM-AVI and comparator agents (amikacin, aztreonam, cefiderocol, colistin, ertapenem, minocycline, tigecycline, and tobramycin) was evaluated separately against *Enterobacterales* that were MBL-positive. Susceptibility was interpreted primarily using 2025 EUCAST breakpoints (ATM-AVI, MIC ≤ 4 mg/L) [24]; FDA and CLSI criteria were applied for tigecycline and minocycline, respectively.

Isolates were categorized as MDR according to criteria published in 2012 by the joint European and United States Centers for Disease Control, which define an MDR isolate as one that is nonsusceptible to ≥ 1 agent

in ≥ 3 antimicrobial classes [15]. The isolates that qualified for ESBL screening were: *E. coli, K. pneumoniae, K. oxytoca, K. variicola, P. mirabilis* isolates with ceftazidime MIC ≥ 2 mg/L and/or aztreonam MIC ≥ 2 mg/L [22]. Confirmatory test with clavulanate was not performed to detect ESBL phenotype. *Enterobacterales* isolates with meropenem MIC > 8 mg/L were categorized as CRE according to the 2025 EUCAST breakpoints [24]. Isolates with meropenem MIC ≥ 2 mg/L, classified as CRE according to CLSI, were routinely subjected to molecular characterization following the ATLAS protocol, except for isolates from China mainland.

Molecular analysis

Multiplex PCR and DNA sequencing were used to detect and characterize beta-lactamase genes. Isolates containing one or more of the major MBL genes like NDM, IMP, and VIM were considered as MBL-positive. The presence of serine carbapenemase genes, namely KPC, OXA-48, and OXA-48-like variants (e. g. OXA-163, OXA-181, OXA-232, and OXA-244 [25]), ESBL genes (SHV, TEM, CTX-M- group - 1, 3, 9, 14, 15, 65, 139, 189, 231, LAP and GES) and of acquired AmpC β-lactamases (ACC, ACT, CMY, DHA, CFE) in MBL-positive isolates was also documented. Gene variants, including specific NDM variants, were identified by sequencing and BLAST analysis against reference databases (National Center for Biotechnology Information database (www.ncbi.nlm.nih.gov). Molecular characterization data for CRE isolates were not available for China mainland in the surveillance program. For this study, a random cohort of CRE isolates collected during the same period (2021-2022) in China mainland underwent molecular characterization, and the identified MBL-positive isolates were integrated into the larger MBL-positive cohort to enhance the surveillance scope in the APAC region. This study did not assess non-enzymatic mechanisms of resistance to ATM-AVI, including insertions in Penicillin-Binding Protein 3 (PBP3), porin and efflux pump modifications.

Results

Distribution of Enterobacterales

A total of 10,376 *Enterobacterales* isolates from patients with BSIs were collected from 59 countries representing AfME (n = 1034), APAC (n = 2431), Europe (n = 5253), and Latin America (n = 1658) through the ATLAS program in 2021-2022. The majority of *Enterobacterales* isolates were obtained from non-ICU wards, comprising 71.5% of the total, with regional variations ranging from 59.8% to 76.3% (Supplementary Table 1). *Escherichia coli* was isolated most often (37%), followed by *Klebsiella pneumoniae* (31.9%), *Serratia marcescens* (3.7%), *Klebsiella oxytoca* (3.6%), *Proteus mirabilis* (2.7%), and *Enterobacter cloacae* (2.3%) in accordance with the ATLAS protocol (Supplementary Table 2).

MDR represented 44.4% of the total isolates. The highest MDR rate was observed in the AfME region at 64.7% (669/1034), followed by Latin America at 53.5% (887/1658), APAC at 48.8% (1186/2431), and Europe at 35.4% (1862/5253) (Figure 1). The overall proportion of ESBL screen positive isolates was 18.1% (1447/7997), being variable across regions with AfME at 31.2% (253/812), APAC at 16.3% (314/1931), Europe at 12.9% (518/4020), and Latin America at 29.3% (362/1234) (Figure 2).

CRE (meropenem MIC > 8 mg/L) represented 6.8% (710/10,376) of the total isolates, with AfME (10.3%, 106/1034), Latin America (10.9%, 181/1658), and APAC (9.2%, 223/2431) reporting significantly higher CRE rates compared to Europe (3.8%, 200/5253) (Figure 1). Out of the 710 isolates of CRE, data on CP-CRE were available for 665 isolates in the ATLAS surveillance database, as genotypic characterization data for the 45 CRE isolates from China mainland were not included in the database. Among these, 650 isolates were identified as CP-CRE, with MBL being the most prevalent type of carbapenemase at 52.9% (344/650), and within the MBL category, the NDM subtype was the most prevalent, accounting for 50.8% (330/650) of isolates. The APAC and AfME regions exhibited the highest prevalence of NDM-CRE, at 76.3% (132/173) and 78% (76/103), respectively. In contrast, Europe and Latin America showed lower prevalence at 24.1% (48/199) and 42.3% (74/175), respectively. APAC also had the highest proportion of CRE positive for the OXA-48 and OXA-48-like genotypes, at 63.6% (110/173), followed by AfME at 46.6% (48/103) and Europe at 31.2% (62/199), resulting in an overall prevalence of 34.2% (222/650). KPC-CRE were more prevalent in Latin America (61.1%, 107/175) and Europe (50.8%, 101/199) compared to APAC and AfME, which had a combined prevalence of 32.5% (211/650) (Table 1). When using meropenem MIC of ≥2 mg/L and including isolates obtained from China mainland (N=16), a total of 440 isolates were identified as MBL-positive. NDM-positive was the most frequently detected genotype, representing 92.5% (407/440) of the isolates, with regional prevalence ranging from 81.4% to 100%, largely exceeding VIM- and IMP-positive isolates. Most NDM-positive isolates comprised of NDM-1 (45.7%, 201/440) and NDM-5 (39.5%,174/440). NDM-1 was predominantly found in Europe (71.4%, 50/70) and NDM-5 was more common in APAC (58%, 98/169) (Figure 3). Other NDM subtypes constitute 7.3% of the total MBL isolates, with their overall and regional distribution detailed in supplementary Table 3. VIM accounted for 5.5% (24/440) overall, with higher prevalence in Europe (18.6%, 13/70) and Latin America (9.4%, 10/106) (Figure 3).

MBL + OXA-48 and OXA-48-like isolates were more prevalent than MBL + KPC isolates collectively (24.8%, 109/440 vs. 5.0%, 22/440) and across different regions (20%–43.2% vs. 0.0%–7.1%), except in Latin America (0.9%, 1/106 vs. 14.2%, 15/106). Overall, 81.1% (357/440) of MBL-positive isolates were also ESBL-positive, and 10.7% (47/440) were AmpC-positive (Figure 3). Regarding species distribution, *K. pneumoniae* and *E. coli* constituted 69.5% (306/440) and 11.1% (49/440) of MBL-positive isolates,

respectively. Notably, NDM-5 was more prevalent in *E. coli* at 83.7% (41/49), whereas both NDM-1 and NDM-5 were frequent in *K. pneumoniae* (46.4%, 142/306 and 41.2%, 126/306 respectively) (Supplementary Table 4).

Antimicrobial activity of ATM-AVI and other antimicrobials against Enterobacterales

Overall, 99.8% (N = 10,376) of isolates were inhibited by ATM-AVI at MIC ≤4 mg/L (EUCAST clinical breakpoint for susceptibility), with MIC_{50} and MIC_{90} values of 0.03 mg/L and 0.12 mg/L, respectively. Among comparator antimicrobials, meropenem, tigecycline, amikacin, and ceftazidime/avibactam demonstrated high activity with susceptibility rates >90% (Table 2). ATM-AVI demonstrated potent antimicrobial activity against *E. coli* and *K. pneumoniae*, with susceptibility rates of 99.7% ($MIC_{50/90}$, 0.03/0.06 mg/L) and 99.9% ($MIC_{50/90}$, 0.06/0.25 mg/L), respectively, with only 10 *E. coli* and 3 *K. pneumoniae* isolates exhibiting MICs >4 mg/L. Among comparator antimicrobials, colistin, meropenem, tigecycline, amikacin, ceftazidime/avibactam, and ceftolozane/tazobactam demonstrated high activity with susceptibility >90% against *E. coli*, while only colistin, tigecycline, and ceftazidime/avibactam exhibited high activity (>90% susceptibility) against *K. pneumoniae* (Supplementary Table 5). ATM-AVI showed similar activity across all regions, inhibiting >99% of isolates at MIC ≤4 mg/L, while some geographic variability was observed with activities of meropenem, tigecycline, amikacin, and ceftazidime/avibactam (Supplementary Table 6).

Antimicrobial activity of ATM-AVI and other antimicrobials against MDR isolates

ATM-AVI demonstrated consistently high activity against 4,604 MDR isolates, with susceptibility rate of 99.6% at MIC ≤ 4 mg/L (MIC_{50}/MIC_{90}, 0.06/0.5 mg/L). Among comparators, only tigecycline inhibited >90% of isolates at MIC ≤2 (FDA clinical breakpoint for susceptibility) (Table 3). ATM-AVI inhibited 99.4% of *E. coli* isolates at MIC ≤4 mg/L, with MIC_{50} and MIC_{90} values of 0.25 mg/L and 1 mg/L, respectively. Tigecycline (99.7%), colistin (99.2%), meropenem (96.9%), and amikacin (93.1%) also exhibited high activity against *E. coli* isolates. ATM-AVI displayed robust activity against *K. pneumoniae* isolates, inhibiting 99.9% at MIC <4 mg/L, with MIC_{50} and MIC_{90} values of 0.12/0.5 mg/L. Among comparators, tigecycline (95%) and colistin (91.2%) showed potent activity (Supplementary Table 7). Across all regions, ATM-AVI inhibited >99% of MDR isolates at MIC ≤4 mg/L, with MIC_{50} of 0.06 mg/L and MIC_{90} ranging between 0.25 and 0.5 mg/L (Supplementary Table 8).

Antimicrobial activity of ATM-AVI and other antimicrobials against ESBL screen positive isolates

ATM-AVI exhibited robust activity against 1447 ESBL screen positive isolates, inhibiting 99.9% of isolates at MIC ≤4 mg/L with MIC_{50} and MIC_{90} values of 0.12/0.5 mg/L. Among comparators, tigecycline (96.5%)

and colistin (91.4%) displayed strong activity (Table 4). ATM-AVI inhibited 99.8% ($MIC_{50/90}$, 0.06/0.12 mg/L) of *E. coli* isolates and all ($MIC_{50/90}$, 0.12/0.5 mg/L) *K. pneumoniae* isolates at MIC ≤4 mg/L. Meropenem inhibited 94.3% of *E. coli* isolates but showed a lower activity against *K. pneumoniae*, inhibiting 45.9% of isolates (Supplementary Table 9). Tigecycline and colistin maintained high potent activity against both *E. coli* and *K. pneumoniae* isolates. Geographically, ATM-AVI displayed excellent activity, with susceptibility rates of 100% in AfME, Europe, and Latin America, and 99.7% in the APAC region at MIC ≤4 mg/L (Supplementary Table 10).

Antimicrobial activity of ATM-AVI and other antimicrobials against CRE phenotypes

ATM-AVI demonstrated high activity against 710 CRE isolates, inhibiting 98.9% of isolates at MIC of ≤4 mg/L and achieving MIC_{50} and MIC_{90} of 0.25/1 mg/L. Comparator agents exhibited lower activity, with only tigecycline covering >90% of isolates (Table 5). ATM-AVI inhibited 82.2% of CRE *E. coli* isolates, with MIC_{50} and MIC_{90} values of 2 mg/L and 8 mg/L, respectively. ATM-AVI inhibited all CRE *K. pneumoniae* isolates, with MIC_{50} and MIC_{90} values of 0.25 mg/L and 0.5 mg/L, respectively (Supplementary Table 11).

Antimicrobial activity of ATM-AVI and other antimicrobials against MBL-positive Enterobacterales including co-producers

ATM-AVI maintained potent activity against 440 MBL-positive *Enterobacterales*, with MIC_{50} and MIC_{90} values of 0.25 mg/L and 1 mg/L, respectively, inhibiting 97% of isolates at MIC ≤4 mg/L. Among comparators, tigecycline (97.5%) and colistin (80.5%) were active, while cefiderocol showed moderate activity (52.7%) (Table 6). ATM-AVI inhibited 75.5% of MBL-positive *E. coli* isolates at MIC ≤4 mg/L ($MIC_{50/90}$, 2/16 mg/L), whereas tigecycline and colistin inhibited all isolates. Cefiderocol demonstrated reduced activity to MBL-positive *E. coli* at 36.7%. All MBL-positive *K. pneumoniae* isolates were inhibited by ATM-AVI at MIC ≤4 mg/L ($MIC_{50/90}$, 0.25/0.5 mg/L). Tigecycline, colistin, and cefiderocol inhibited 98%, 79.7%, and 55.9% of isolates, respectively (Supplementary Table 13). ATM-AVI inhibited 100% of MBL-positive *Enterobacterales* from AfME, Europe, and Latin America, while 92.3% were inhibited from the APAC region (MIC ≤4 mg/L) (Supplementary Table 14).

ATM-AVI inhibited 96.8% of isolates carrying NDM genotype ($MIC_{50/90}$, 0.25/1 mg/L) (Table 7) at MIC of ≤4 mg/L. Among comparator agents, tigecycline showed potent activity at 97.5%, and colistin was active at 81.3%, while cefiderocol exhibited moderate activity at 51.1% against NDM-carrying isolates. ATM-AVI inhibited 75% ($MIC_{50/90}$, 2/16 mg/L) of *E. coli* and 100% ($MIC_{50/90}$, 0.25/0.5 mg/L) of *K. pneumoniae* isolates carrying NDM at an MIC of ≤4 mg/L. Tigecycline and colistin displayed complete activity (100%) to *E. coli* isolates carrying NDM, that showed high resistance to cefiderocol at 64.6%. Tigecycline (98.3%)

and colistin (81.4%) maintained high activity, and cefiderocol was active against 55% of *K. pneumoniae* isolates carrying NDM (Supplementary Table 17). ATM-AVI inhibited 100% of NDM-carrying *Enterobacterales* from the AfME, Europe, and Latin America regions at a concentration of ≤4 mg/L, while 91.9% ($MIC_{50/90}$, 0.5/4 mg/L) of isolates were inhibited from the APAC region (Supplementary Table 18). ATM-AVI also displayed robust activity against NDM-1 (99%; $MIC_{50/90}$, 0.25/0.5 mg/L) and NDM-5 (93.7%; $MIC_{50/90}$, 0.5/4 mg/L) positive *Enterobacterales* at an MIC of ≤4 mg/L. ATM-AVI maintained complete activity against NDM-5-positive *K. pneumoniae* (N=126), whereas 73.2% of NDM-5-positive *E. coli* (N=30/41) were inhibited by ATM-AVI at an MIC of ≤4 mg/L. Tigecycline retained high activity for both NDM-1 (96%) and NDM-5 (98.8%), whereas cefiderocol showed moderate activity (NDM-1 at 49.5% and NDM-5 at 54.6%) (Supplementary Table 15 and 16). ATM-AVI achieved complete activity (100%) against isolates carrying VIM ($MIC_{50/90}$, 0.5/2 mg/L) (Table 8) and IMP ($MIC_{50/90}$, 0.25/0.5 mg/L) (Table 9) genotypes at MIC of ≤4 mg/L. Colistin (58.3%) and cefiderocol (66.7%) showed moderate activity against VIM-carrying isolates, while tigecycline retained high activity (95.8%). Tigecycline and colistin displayed complete activity (100%) against IMP-carrying isolates, whereas cefiderocol showed moderate activity at 72.7%.

ATM-AVI demonstrated complete activity (100%) against isolates co-harboring both MBL and KPC ($MIC_{50/90}$, 0.5/1 mg/L) and it inhibited 98.2% of isolates carrying MBL in combination with OXA-48 and OXA-48-like genes ($MIC_{50/90}$, 0.5/1 mg/L). ATM-AVI inhibited 97.8% of isolates co-harboring MBL and ESBL ($MIC_{50/90}$, 0.25/1 mg/L), while 78.7% of isolates co-carrying MBL and AmpC were susceptible to ATM-AVI ($MIC_{50/90}$, 1/16 mg/L) at a concentration of ≤4 mg/L. ATM-AVI effectively inhibited all *K. pneumoniae* isolates co-harboring MBL and AmpC (N=26), while 41.2% of *E. coli* isolates co-harboring MBL and AmpC (N=7/17) were susceptible to ATM-AVI at an MIC of ≤4 mg/L. Tigecycline showed potent activity (>90%) for all categories, while colistin maintained activity against MBLs co-carrying OXA-48 and OXA-48-like (86.2%), AmpC (89.4%), and ESBL (82.1%). Cefiderocol demonstrated moderate activity against MBLs co-carrying OXA-48 and OXA-48-like (61.5%), KPC (45.5%), and ESBL (54.3%), but had low activity against MBLs co-carrying AmpC isolates, with a resistance rate of 70.2% (Table 10-13).

Discussion

The growing prevalence of BSIs caused by MDR *Enterobacterales* poses a significant therapeutic challenge. This study evaluated the *in vitro* activity of ATM-AVI, against *Enterobacterales*, including MDR isolates that produce diverse clinically relevant ß-lactamases, either alone or in combination. ATM-AVI showed potent *in vitro* activity against MBL-positive CRE bloodstream isolates, inhibiting 97% of MBL-positive *Enterobacterales* isolates at MIC ≤4 mg/L. This performance surpassed comparator agents,

including cefiderocol, which exhibited activity in only 52.7% of isolates. ATM-AVI is a combination therapy that leverages aztreonam's inherent stability to MBLs and avibactam's ability to neutralize ESBL, KPC, OXA-48 and OXA-48-like, and AmpC β-lactamases, thereby restoring ATM's activity against MBL-producing isolates [26]. Furthermore, our study confirms that NDM-enzymes are the most prevalent MBLs globally, with NDM-5 gaining prominence, particularly in Asia, while VIM and IMP remain comparatively rare.

Globally, NDM was the most predominant genotype among 650 CP-CRE isolates (50.8%, 330/650), followed by OXA-48 and OXA-48-like genotypes at 34.2% (222/650) and KPC at 32.5% (211/650). These findings align with recent analysis observing NDM as the most frequent carbapenemase at 52.2%, followed by OXA-48 and OXA-48-like at 30.1% and KPC at 14.7% among CP-*E. coli* [27]. The distribution of CP-CRE isolates reveals significant regional variability. NDM was most prevalent in APAC (76.3%, 132/173) and AfME (73.8%, 76/103) regions. KPC predominated in Europe (50.8%, 101/199) and LATAM (61.1%, 107/175) and was nearly absent in AfME and APAC regions. Dadashi et al. reported NDM-positive *E. coli* in Asia at 82.6% versus 12.9% in Europe [12] while Kedišaletše et al. identified NDM (43.1%) and OXA-48 and OXA-48-like (42.9%) most frequently in Africa [28]. In a China study, carbapenemase genes were detected in 81.8% of CRE-BSI isolates, with NDM present in 66.7% (36/54) and KPC in 29.6% (16/54) [29]. An Argentinian study reported comparable prevalence of MBL (44.2%) and KPC (36.1%) among CRE-BSI isolates [30]. A study conducted in Italy and Greece reported a predominance of NDM-producing strains, comprising 80.4% (82/102) of CRE-BSI isolates, while VIM producers accounted for 19.6% (20/102); OXA-type carbapenemases were far less common [31]. The APAC region also shows a significant proportion of OXA-48 and OXA-48-like enzymes (63.6%, 110/173) consistent with Alvisi et al., who found OXA-48 and OXA-48-like to be most common in India, South Africa, and Russia, but rare in China [13]. Thailand demonstrated a high rate of co-occurrence between NDM-1 and OXA-48, with 51.7% of isolates expressing both, 31.7% expressing OXA-48 alone, and 15.0% expressing NDM-1 alone among CRE-BSI isolates [32]. Overall, these findings align with current CP-CRE epidemiology and emphasize the importance of regional and local surveillance to guide CRE-treatment decisions.

The current study confirms NDM as the most prevalent genotype among 440 MBL-positive *Enterobacterales* isolates, representing 92.5% (407/440) of the total, while VIM and IMP genotypes are notably less common at 5.5% (24/440) and 2.5% (11/440), respectively. Regionally, NDM-1 was predominantly found in Europe (71.4%, 50/70), while NDM-5 was more common in the APAC region (58%, 98/169) consistent with those reported by Kanj et al. [10] . Similar geographic patterns have been reported for IMP and VIM by Kanj et al [10]. India accounted for 25.7% of all MBL isolates with 76% (86/113) identified as NDM-5 and 19.5% (22/113) as NDM-1 aligning with recent data indicating a shift

from NDM-1 to NDM-5 [7, 33, 34]. In China mainland, 93.6% (15/16) of MBL isolates were NDMs, evenly split between NDM-1 and NDM-5. These trends reinforce the dominant role of NDM genotypes in both India and China and reflect broader regional patterns of carbapenemase prevalence in bloodstream infections.

This study evaluated 10,376 *Enterobacterales* isolates from patients with BSIs collected between 2021 and 2022 through ATLAS Global Surveillance Program. The study analyzed isolates from regions where AMR rates are generally high [35], including Africa/Middle East, Asia-Pacific, Europe, and Latin America. ATM-AVI exhibited robust antimicrobial activity, inhibiting 99.8% (MIC ≤4 mg/L) of *Enterobacterales* and consistently maintained robust activity across various phenotypes, including CRE isolates at 98.9%. These findings are consistent with global reports confirming ATM-AVI's potent antimicrobial profile [8, 15, 36-38]. Most recently, Sader et al. (2025) found ATM-AVI active (MIC ≤4 mg/L) against 99.8% and >99.9% of *Enterobacterales* from Eastern Europe (E-EU) and the Mediterranean region and Western Europe (W-EU), respectively, with strong activity against CRE isolates (99.6% and 99.7%, respectively) [39].

The present study found that ATM-AVI inhibited 97% of all MBL-positive *Enterobacterales*, maintaining > 96% susceptibility across key genotypes: NDM (96.8%), IMP (100%), and VIM (100%) at an MIC ≤4 mg/L, with MIC90 values ranging from 0.5 mg/L to 2 mg/L. Activity remained high against isolates co-carrying ESBLs and other carbapenemases such as KPC and OXA-48 and OXA-48-like enzymes, consistent with prior global reports.[17, 37, 40, 41]. While ATM-AVI inhibited 96.8% of NDM-positive isolates overall, regional variation was noted. In APAC, susceptibility decreased to 91.9% with an MIC90 of 4 mg/L driven by lower *in vitro* activity against NDM-5 (93.7%) compared to NDM-1 (99%). ATM-AVI retained full activity against NDM-positive *K. pneumoniae*, but activity was reduced in *E. coli* (75% susceptible; MIC90: 16 mg/L), particularly among ten isolates from India carrying NDM-5 and CMY AmpC variants. Overall, MBL-positive isolates co-carrying AmpC-type β-lactamases showed 78.7% susceptibility to ATM-AVI at MIC ≤4 mg/L, with an elevated MIC90 value of 16 mg/L. Previous studies have linked reduced ATM-AVI susceptibility in *E. coli* to PBP3 insertions and CMY enzymes [42-44]. Periasamy et al., (2020) observed raised ATM-AVI MICs (2–32 mg/L) in 23% of *E. coli* isolates, attributing this to PBP3 insertions in NDM+ *E. coli* [43].. Tellapragada et al., (2024) reported PBP3 inserts (YRIK/YRIN) in all 20 resistant *E. coli* isolates studied, with CMY-42 present in five. They identified several mutations in ftsI (encoding PBP3) and regulatory genes of outer membrane proteins and efflux pumps, concluding that resistance to ATM-AVI results from a complex interplay of target alterations, deactivating enzymes, and decreased permeability [45]. Although this study did not assess PBP3 insertion mutations, the elevated MICs observed in APAC region may reflect similar resistance mechanisms. These

observations highlight the importance of ongoing susceptibility monitoring and molecular characterization to better understand potential resistance trends.

In this study, cefiderocol showed moderate activity against key genetic variants, including NDM (51.1%; NDM-1 at 49.5% and NDM-5 at 54.6%), IMP (72.7%), and VIM (66.7%). Its activity was notably reduced in isolates co-carrying MBL and KPC (45.5%) and was particularly low against those co-harboring MBL and AmpC enzymes, with 70.2% resistance. Among species, cefiderocol inhibited 55% of NDM-positive *K. pneumoniae* but showed limited activity against NDM-positive *E. coli* (35.4%). In comparison, the Sader et al., (2025) study found cefiderocol to be active against 74.8% and 87.6% of CREs from E-EU and W-EU, and overall, 63.5% of MBL isolates were inhibited by cefiderocol, with 59.4% in E-EU and 77.1% in W-EU [39]. This suggests a higher activity rate against MBL isolates in the Sader et al study compared to the current study. Additionally, a systematic review reveals 42–59% nonsusceptibility to cefiderocol in certain cohorts among NDM, KPC and AmpC variants conferring resistance to ceftazidime-avibactam [46]. Surveillance studies indicate that cefiderocol exhibits higher MIC values when tested against NDM-positive isolates compared to VIM-positive isolates [47]. Collectively, the findings across these studies highlight cefiderocol's varying activity against different genetic variants, with notable resistance patterns in NDM-positive isolates.

It is crucial to consider the study's limitations when interpreting the findings. The predetermined number of isolates collected at each site or center during the surveillance may constrain the generalizability of the study findings for epidemiological assessments of the broader population. Additionally, the number of samples were lower for some resistant phenotypes and regions, which needs to be carefully weighted while interpreting the study findings. One of the potential limitations of this study includes the variation in data sources for MBL-positive isolates. Molecular characterization data for CRE isolates were not available from China mainland in the surveillance program; however, data on MBL-positive isolates from China mainland were obtained as an ancillary project and incorporated into the analysis to strengthen the surveillance scope in the APAC region. In addition, the differences in data processing across the platform may introduce some inconsistencies. This study included isolates collected up to the year 2022; more recent samples were not incorporated due to the update schedule of ATLAS data. On the ATLAS platform, data is typically collected throughout the year, after which molecular analysis of the samples is conducted causing operational delays.

Conclusion

The findings presented herein demonstrate ATM-AVI’s significant clinical potential for treating BSIs caused by *Enterobacterales* that produce diverse β-lactamases whether produced alone or in combination.

ATM-AVI exhibited remarkable activity, inhibiting over 99% of *Enterobacterales* isolates across all regions at an MIC of ≤4 mg/L and outperforming comparator agents. It consistently maintained robust activity across various resistant phenotypes, including MDR and CRE, as well as against ESBL screen positive and MBL-positive *Enterobacterales*. The ability of ATM-AVI to treat the co-production of multiple enzymes in a single isolate underscores its comprehensive β-lactamase coverage, which is unique among currently available agents. By addressing a critical gap in the current antibiotic arsenal, ATM-AVI offers a promising option for the management of severe infections including BSIs. Continuous monitoring of resistance patterns will be essential to guide and optimize its use across diverse geographical settings.

Acknowledgements

All investigative sites were recruited, and study supplies were provided by IHMA, Inc. Analysis of the final MIC and molecular data was performed by IHMA. The authors would like to acknowledge Dimpal Thakkar, Nehali Save and Shrestha Priyadarsini from International Medical Communications and Content (IMCC), Pfizer Ltd. for providing medical writing and editorial assistance. Pfizer's generative artificial intelligence (AI) assisted technology, MAIA (Medical Artificial Intelligence Assistant) was used for language editing and refinement of this manuscript. All scientific content, analysis, and conclusions were independently developed by the authors.

Funding

This work was supported by Pfizer Inc. as part of the ATLAS global surveillance program. Pfizer holds the marketing authorization for aztreonam-avibactam.

Declaration of interests

SK, NM, GS and KKP are employees of Pfizer and may hold stocks or stock options. GMR has received research grants/materials, and/or been a consultant, and/or received honoraria from Ada, Alifax, Angelini, Arrow, Biomedical Service, bioMérieux, Cepheid, Hain Life Sciences, Menarini, MSD, Meridian, Nordic Pharma, Pfizer, Q-linea, Quantamatrix, Quidel, Qvella, Relab, SD Biosensor, Seegene, Shionogi, Symcel, and Viatris.

Ethical approval: Not required.

CRediT authorship contribution statement: The authors listed, wrote, edited, and/or reviewed this manuscript.

Availability of data: The datasets generated during and/or analyzed during the current study are available from the corresponding author on reasonable request.

References

1. Theuretzbacher U. Global antimicrobial resistance in Gram-negative pathogens and clinical need. Curr Opin Microbiol. 2017;39:106-12.10.1016/j.mib.2017.10.028
2. Voor In 't Holt AF, Mourik K, Beishuizen B, van der Schoor AS, Verbon A, Vos MC, et al. Acquisition of multidrug-resistant *Enterobacterales* during international travel: a systematic review of clinical and microbiological characteristics and meta-analyses of risk factors. Antimicrob Resist Infect Control. 2020;9(1):71.10.1186/s13756-020-00733-6
3. Caliskan-Aydogan O, Alocilja EC. A Review of Carbapenem Resistance in *Enterobacterales* and Its Detection Techniques. Microorganisms. 2023;11(6).10.3390/microorganisms11061491
4. <risk-assessment-carbapenem-resistant-*Enterobacterales*-third-update-february-2025_0.pdf>.10.2900/8752612
5. Rankin DA, Stahl A, Sabour S, Khan MA, Armstrong T, Huang JY, et al. Changes in Carbapenemase-Producing Carbapenem-Resistant *Enterobacterales*, 2019 to 2023. Ann Intern Med. 2025.10.7326/annals-25-02404
6. Wilson GM, Fitzpatrick MA, Suda KJ, Smith BM, Gonzalez B, Jones M, et al. Comparative effectiveness of antibiotic therapy for carbapenem-resistant *Enterobacterales* (CRE) bloodstream infections in hospitalized US veterans. JAC Antimicrob Resist. 2022;4(5):dlac106.10.1093/jacamr/dlac106
7. Mitra S, Naha S, Chakraborty J, De S, Kaur H, Majumdar T, et al. Diversity of mobile genetic elements in carbapenem-resistant *Enterobacterales* isolated from the intensive care units of a tertiary care hospital in Northeast India. Front Microbiol. 2025;16:1543427.10.3389/fmicb.2025.1543427
8. Pierard D, Hermsen ED, Kantecki M, Arhin FF. Antimicrobial Activities of Aztreonam-Avibactam and Comparator Agents against *Enterobacterales* Analyzed by ICU and Non-ICU Wards, Infection Sources, and Geographic Regions: ATLAS Program 2016-2020. Antibiotics (Basel). 2023;12(11).10.3390/antibiotics12111591
9. Zakhour J, El Ayoubi LW, Kanj SS. Metallo-beta-lactamases: mechanisms, treatment challenges, and future prospects. Expert Rev Anti Infect Ther. 2024;22(4):189-201.10.1080/14787210.2024.2311213
10. Kanj SS, Kantecki M, Arhin FF, Gheorghe M. Epidemiology and outcomes associated with MBL-producing *Enterobacterales*: A systematic literature review. Int J Antimicrob Agents. 2025;65(4):107449.10.1016/j.ijantimicag.2025.107449
11. Suay-Garcia B, Perez-Gracia MT. Present and Future of Carbapenem-resistant Enterobacteriaceae (CRE) Infections. Antibiotics (Basel). 2019;8(3).10.3390/antibiotics8030122
12. Dadashi M, Yaslianifard S, Hajikhani B, Kabir K, Owlia P, Goudarzi M, et al. Frequency distribution, genotypes and prevalent sequence types of New Delhi metallo-beta-lactamase-producing Escherichia coli among clinical isolates around the world: A review. J Glob Antimicrob Resist. 2019;19:284-93.10.1016/j.jgar.2019.06.008
13. Alvisi G, Curtoni A, Fonnesu R, Piazza A, Signoretto C, Piccinini G, et al. Epidemiology and Genetic Traits of Carbapenemase-Producing *Enterobacterales*: A Global Threat to Human Health. Antibiotics (Basel). 2025;14(2).10.3390/antibiotics14020141
14. Mauri C, Maraolo AE, Di Bella S, Luzzaro F, Principe L. The Revival of Aztreonam in Combination with Avibactam against Metallo-beta-Lactamase-Producing Gram-Negatives: A Systematic Review of In Vitro Studies and Clinical Cases. Antibiotics (Basel). 2021;10(8).10.3390/antibiotics10081012
15. Wise MG, Karlowsky JA, Mohamed N, Kamat S, Sahm DF. In vitro activity of aztreonam-avibactam against *Enterobacterales* isolates collected in Latin America, Africa/Middle East, Asia,

and Eurasia for the ATLAS Global Surveillance Program in 2019-2021. Eur J Clin Microbiol Infect Dis. 2023;42(9):1135-43.10.1007/s10096-023-04645-2
16. Rossolini GM, Arhin FF, Kantecki M. In vitro activity of aztreonam-avibactam and comparators against Metallo-β-Lactamase-producing *Enterobacterales* from ATLAS Global Surveillance Program, 2016-2020. J Glob Antimicrob Resist. 2024;36:123-31.10.1016/j.jgar.2023.12.027
17. Sader HS, Castanheira M, Kimbrough JH, Kantro V, Mendes RE. Aztreonam/avibactam activity against a large collection of carbapenem-resistant *Enterobacterales* (CRE) collected in hospitals from Europe, Asia and Latin America (2019-21). JAC Antimicrob Resist. 2023;5(2):dlad032.10.1093/jacamr/dlad032
18. Carmeli Y, Cisneros JM, Paul M, Daikos GL, Wang M, Torre-Cisneros J, et al. Aztreonam-avibactam versus meropenem for the treatment of serious infections caused by Gram-negative bacteria (REVISIT): a descriptive, multinational, open-label, phase 3, randomised trial. Lancet Infect Dis. 2025;25(2):218-30.10.1016/S1473-3099(24)00499-7
19. Daikos GL, Cisneros JM, Carmeli Y, Wang M, Leong CL, Pontikis K, et al. Aztreonam-avibactam for the treatment of serious infections caused by metallo-β-lactamase-producing Gram-negative pathogens: a Phase 3 randomized trial (ASSEMBLE). JAC Antimicrob Resist. 2025;7(4):dlaf131.10.1093/jacamr/dlaf131
20. EMBLAVEO®summary of product characteristics; Pfizer, 2024. [Available from: https://ec.europa.eu/health/documents/community-register/2024/20240422162367/anx_162367_en.pdf.
21. EMBLAVEO® US Prescribing Information; AbbVie, 2025., https://www.accessdata.fda.gov/drugsatfda_docs/label/2025/217906Orig1s000lbl.pdf
22. ATLAS Surveillance. Antimicrobial Testing Leadership and Surveillance. [Available from: https://atlas-surveillance.com/.
23. CLSI. 2025. Performance standards for antimicrobial susceptibility testing. M100. Thirty-Fifth Edition. CLSI, Wayne, PA, USA.
24. The European Committee on Antimicrobial Susceptibility Testing. Breakpoint tables for interpretation of MICs and zone diameters. Version 15.0, 2025. https://www.eucast.org.
25. [Available from: http://bldb.eu/alignment.php?align=D:OXA-48-like.
26. Al Musawa M, Bleick CR, Herbin SR, Caniff KE, Van Helden SR, Rybak MJ. Aztreonam-avibactam: The dynamic duo against multidrug-resistant gram-negative pathogens. Pharmacotherapy. 2024;44(12):927-38.10.1002/phar.4629
27. Li Y, Sun X, Dong N, Wang Z, Li R. Global distribution and genomic characteristics of carbapenemase-producing Escherichia coli among humans, 2005-2023. Drug Resist Updat. 2024;72:101031.10.1016/j.drup.2023.101031
28. Kedisaletse M, Phumuzile D, Angela D, Andrew W, Mae NF. Epidemiology, risk factors, and clinical outcomes of carbapenem-resistant *Enterobacterales* in Africa: A systematic review. J Glob Antimicrob Resist. 2023;35:297-306.10.1016/j.jgar.2023.10.008
29. Zhang L, Zhen S, Shen Y, Zhang T, Wang J, Li J, et al. Bloodstream infections due to Carbapenem-Resistant Enterobacteriaceae in hematological patients: assessment of risk factors for mortality and treatment options. Ann Clin Microbiol Antimicrob. 2023;22(1):41.10.1186/s12941-023-00586-y
30. Echegorry M, Marchetti P, Sanchez C, Olivieri L, Faccone D, Martino F, et al. National Multicenter Study on the Prevalence of Carbapenemase-Producing Enterobacteriaceae in the Post-COVID-19 Era in Argentina: The RECAPT-AR Study. Antibiotics (Basel). 2024;13(12).10.3390/antibiotics13121139

31. Falcone M, Daikos GL, Tiseo G, Bassoulis D, Giordano C, Galfo V, et al. Efficacy of Ceftazidime-avibactam Plus Aztreonam in Patients With Bloodstream Infections Caused by Metallo-β-lactamase-Producing *Enterobacterales*. Clin Infect Dis. 2021;72(11):1871-8.10.1093/cid/ciaa586
32. Karnmueng P, Montakantikul P, Paiboonvong T, Plongla R, Chatsuwan T, Chumnumwat S. Mortality factors and antibiotic options in carbapenem-resistant *Enterobacterales* bloodstream infections: Insights from a high-prevalence setting with co-occurring NDM-1 and OXA-48. Clin Transl Sci. 2024;17(6):e13855.10.1111/cts.13855
33. Snyder BM, Montague BT, Anandan S, Madabhushi AG, Pragasam AK, Verghese VP, et al. Risk factors and epidemiologic predictors of blood stream infections with New Delhi Metallo-b-lactamase (NDM-1) producing Enterobacteriaceae. Epidemiol Infect. 2019;147:e137.10.1017/s0950268819000256
34. Nabarro LEB, Shankar C, Pragasam AK, Mathew G, Jeyaseelan V, Veeraraghavan B, et al. Clinical and Bacterial Risk Factors for Mortality in Children With Carbapenem-resistant Enterobacteriaceae Bloodstream Infections in India. Pediatr Infect Dis J. 2017;36(6):e161-e6.10.1097/inf.0000000000001499
35. ResistanceMap C. [Available from: https://resistancemap.cddep.org/About.php.
36. Rossolini GM, Stone G, Kantecki M, Arhin FF. In vitro activity of aztreonam/avibactam against isolates of *Enterobacterales* collected globally from ATLAS in 2019. J Glob Antimicrob Resist. 2022;30:214-21.10.1016/j.jgar.2022.06.018
37. Rossolini GM, Arhin FF, Kantecki M. In vitro activity of aztreonam-avibactam and comparators against Metallo-beta-Lactamase-producing *Enterobacterales* from ATLAS Global Surveillance Program, 2016-2020. J Glob Antimicrob Resist. 2024;36:123-31.10.1016/j.jgar.2023.12.027
38. Sader HS, Mendes RE, Kimbrough JH, Hubler CM, Castanheira M. Activity of Aztreonam/Avibactam and Recently Approved beta-Lactamase Inhibitor Combinations against *Enterobacterales* and Pseudomonas aeruginosa from Intensive Care Unit and Non-Intensive Care Unit Patients. Antibiotics (Basel). 2024;13(6).10.3390/antibiotics13060564
39. Sader HS, Kimbrough JH, Winkler ML, Castanheira M, Mendes RE. Aztreonam/avibactam activity against *Enterobacterales* from European medical centres: summary of 5 years of surveillance prior to approval for clinical use (2019-2023). J Antimicrob Chemother. 2025;80(8):2070-9.10.1093/jac/dkaf161
40. Karlowsky JA, Kazmierczak KM, de Jonge BLM, Hackel MA, Sahm DF, Bradford PA. In Vitro Activity of Aztreonam-Avibactam against Enterobacteriaceae and Pseudomonas aeruginosa Isolated by Clinical Laboratories in 40 Countries from 2012 to 2015. Antimicrob Agents Chemother. 2017;61(9).10.1128/AAC.00472-17
41. Kazmierczak KM, Biedenbach DJ, Hackel M, Rabine S, de Jonge BL, Bouchillon SK, et al. Global Dissemination of blaKPC into Bacterial Species beyond Klebsiella pneumoniae and In Vitro Susceptibility to Ceftazidime-Avibactam and Aztreonam-Avibactam. Antimicrob Agents Chemother. 2016;60(8):4490-500.10.1128/aac.00107-16
42. Alm RA, Johnstone MR, Lahiri SD. Characterization of Escherichia coli NDM isolates with decreased susceptibility to aztreonam/avibactam: role of a novel insertion in PBP3. J Antimicrob Chemother. 2015;70(5):1420-8.10.1093/jac/dku568
43. Periasamy H, Joshi P, Palwe S, Shrivastava R, Bhagwat S, Patel M. High prevalence of Escherichia coli clinical isolates in India harbouring four amino acid inserts in PBP3 adversely impacting activity of aztreonam/avibactam. J Antimicrob Chemother. 2020;75(6):1650-1.10.1093/jac/dkaa021
44. Sadek M, Juhas M, Poirel L, Nordmann P. Genetic Features Leading to Reduced Susceptibility to Aztreonam-Avibactam among Metallo-β-Lactamase-Producing Escherichia coli Isolates. Antimicrob Agents Chemother. 2020;64(12).10.1128/aac.01659-20

45. Tellapragada C, Razavi M, Peris PS, Jonsson P, Vondracek M, Giske CG. Resistance to aztreonam-avibactam among clinical isolates of Escherichia coli is primarily mediated by altered penicillin-binding protein 3 and impermeability. Int J Antimicrob Agents. 2024;64(3):107256.10.1016/j.ijantimicag.2024.107256
46. Karakonstantis S, Rousaki M, Kritsotakis EI. Cefiderocol: Systematic Review of Mechanisms of Resistance, Heteroresistance and In Vivo Emergence of Resistance. Antibiotics (Basel). 2022;11(6).10.3390/antibiotics11060723
47. Kaye KS, Naas T, Pogue JM, Rossolini GM. Cefiderocol, a Siderophore Cephalosporin, as a Treatment Option for Infections Caused by Carbapenem-Resistant *Enterobacterales*. Infect Dis Ther. 2023;12(3):777-806.10.1007/s40121-023-00773-6

Response to Reviewer Comments

Journal: Microbiology Spectrum
Manuscript Title: *In vitro* activity of aztreonam-avibactam and comparators against *Enterobacterales* isolated from bloodstream infections: ATLAS surveillance, 2021-2022
Manuscript ID: Spectrum00241-26
Corresponding Author: Katherine K. Perez

General Response

We thank the Editor and the Reviewers for their careful review of our manuscript and for their constructive comments. We have addressed all comments and revised the manuscript accordingly.

In addition to the changes made in response to the reviewer comments, we have made editorial revisions throughout the manuscript to improve clarity, flow, readability, and consistency. These changes do not affect the scientific content or conclusions of the manuscript.

Below, we provide a point-by-point response to each comment. Changes made to the manuscript are indicated with line numbers in the revised tracked-changes version.

**Reviewer 1**			
**No**	**Comment**	**Response**	**Location in manuscript**
**1**	Lines 35, 37 and 41 - describing inhibition of isolates at an 'MIC of {less than or equal to} 4mg/L' is confusing, using the term 'concentration' or 'breakpoint' is more fitting here. An MIC value describes the readout of the test against an individual isolate and would not be encompassing of the isolates which were not inhibited at this drug concentration (if broth microdiliution was the method used to measure inhibition). Readers unfamiliar with the test method may confuse this as isolates with ATM-AVI susceptibility {less than or equal to} 4mg/L. The authors are using this in the way that you would describe MIC50/90, however 'breakpoint' is more commonly applied when	We thank the reviewer for this clarifying comment. We agree that the original wording could be misleading and have revised the text throughout to avoid describing isolate inhibition using "MIC of ≤ 4 mg/L." The revised text now refers to inhibition at the EUCAST breakpoint (≤4 mg/L), which more accurately reflects the experimental context and reduces the risk of misinterpretation by	Abstract, lines - 35, 37, 38 and 41, and throughout the manuscript

	discussing overall isolate susceptibility in this manner	readers unfamiliar with the methodology.	
2	Line 22 - Why was cefotaxime not also applied as an ESBL screen? Ceftazidime has poorer capture of CTX-M type ESBL variants than ceftazidime, hence why CAZ and CTX in conjunction with ATM are both used in most ESBL screen tests. Some CTX type variants may have been missed using this method, especially in regions where they are more common than TEM/SHV ESBLs.	We thank the reviewer for this important observation. Cefotaxime was not included in the ESBL screening panel because it is not part of the routine ATLAS surveillance testing protocol. As a result, ESBL screening was based only on ceftazidime and aztreonam MICs, with isolates showing ceftazidime MIC ≥2 mg/L and/or aztreonam MIC ≥2 mg/L classified as ESBL screen-positive. We acknowledge that this approach may fail to detect some CTX-M-type ESBLs, particularly in settings where these enzymes are more prevalent than TEM/SHV variants. This limitation has now been added to the manuscript, and the terminology has been revised throughout to refer to these isolates as "ESBL screen-positive" rather than "ESBL-positive."	Page 5, lines 113-117; Page 6, lines 150-152; Page 12, lines 354-357

3	Line 111- Line 111 - the caz/avibactam phenotypic selection in itself was technically a screen - it is not clear here how the authors defined isolates as ESBL positive, given that phenotypic confirmatory testing with inhibitors were not used. Does 'qualified' mean sent for downstream ESBL confirmation after having fulfilled the criteria of CAZ MIC {greater than or equal to} 2mg/L, or ATM MIC {greater than or equal to} 2mg/L? Or were isolates fulfilling either of these phenotypic criteria designated as ESBL? If detection of this phenotype was used to select isolates for further ESBL testing, elaborate on the follow-on confirmatory testing method used and how isolates were finally confirmed as 'ESBL positive'. If a CAZ MIC {greater than or equal to} 2mg/L, or ATM MIC {greater than or equal to} 2mg/L phenotype was used as a proxy for ESBL, the authors need to describe this as such, as this method alone is not sufficient to determine true ESBL status - pAmpC producers, or TEM over-expressors could also be selected using these inclusion criteria.	We thank the reviewer for this important observation. We agree that the ceftazidime/avibactam-based phenotypic criteria (ceftazidime MIC ≥2 mg/L and/or aztreonam MIC ≥2 mg/L) function as a screen and, in the absence of confirmatory testing with β-lactamase inhibitors (e.g., clavulanate), cannot definitively distinguish true ESBL producers from plasmid-mediated AmpC producers or TEM/SHV over-expressors.<br><br>To address this, we have made the following revisions: Methods section: We have clarified that isolates meeting the phenotypic criteria were defined as "ESBL screen-positive" rather than confirmed ESBL producers. We have also added an explicit statement that confirmatory testing with clavulanate was not performed and that the screening criteria may also capture plasmid-mediated AmpC	Page 5, lines 113-117; Page 8, lines 213-214

		producers or TEM/SHV over-expressors.<br><br>Results section: We have revised the language to refer to isolates as "meeting ESBL screening criteria" and added a parenthetical noting that this group may include ESBL producers as well as plasmid-mediated AmpC and other β-lactamase producers.	
4	[Lines 130 - 134 - This would be a very nice follow-on study, as understanding the prevalence of non-enzymatic resistance mechanisms to ATM-AVI would be of global clinical interest given the difficulty in identifying them diagnostically (especially in regions where routine molecular testing is not commonplace)]	We thank the reviewer for this insightful comment and agree that characterising non-enzymatic resistance mechanisms to ATM-AVI would be of significant global clinical interest.	Page 11, lines 306-308
**Reviewer 2**			
1	Line 27, the first letter should be capitalized	Thank you for your review. The comment has been addressed	Abstract, Line 27
2	Line 50, none of the three references are related to the lists of WHO or CDC	We thank the reviewer for highlighting this issue. We have addressed this comment by adding the references to the official World Health Organization (WHO) and Centers for Disease	Citations updated on Page 3, line 50, References revised on page 14, Line 408-413

		Control and Prevention (CDC) websites that describe priority pathogen lists. This revision ensures that the references accurately support the statement made in the text.	
3	Line 108, should give the exact values for R, I, and S phenotypes	We thank the reviewer for this comment. We have addressed this by specifying the exact interpretative criteria for ATM-AVI, Tigecycline, and Minocycline in the text, and now explicitly state the susceptibility threshold as S≤4; R>4 mg/L for ATM-AVI, S≤2; I 4; R≥8 mg/L for Tigecycline, and S≤4; I 8; R≥16 mg/L for Minocycline. This revision improves clarity and ensures precise interpretation of resistance phenotypes.	Page 4, Line 108-109
4	Lines 115-117, also need a reference	Thank you for highlighting this need. A reference has been cited.	text updated on page 5, line 120, Added as reference no. 27, revised on page 15, Line 480-482
5	Line 117, "China mainland" should be Chinese mainland. To change it throughout the manuscript.	We thank the reviewer for this comment. We have revised the wording throughout the manuscript	Updated throughout the manuscript

		to "Chinese mainland"	
6	Lines 132-134, repeat, should delete	This comment has been addressed	Page 5, Lines deleted
7	Line 143, in Supplementary Table 2, the CRE detection data for *Citrobacter koseri* are not listed. If the detection rate is zero for this species, it is recommended to clarify this in a footnote or remark	We thank the reviewer for this helpful suggestion. We have addressed this comment by clarifying in Supplementary Table 2 that the CRE detection rate for *Citrobacter koseri* was zero	Supplementary Table 2
8	Line 151, "significantly", should indicate the *P* value	We thank the reviewer for this comment. As no formal statistical analyses were conducted for this comparison, we have removed the term "significantly" from the text to avoid overstating the findings.	Page 6, Line 155
9	Line 157, "78%", should precise to one decimal place. To keep consistent throughout the text.	We thank the reviewer for this comment. We have addressed this by revising the value to one decimal place to maintain consistency throughout the manuscript.	Page 2, Line 41, Page 9, Line 268, updated throughout the manuscript
10	Lines 166-167, in this description, the proportions of NDM subtypes should be calculated using the number of NDM-positive isolates (407) as the denominator, rather than the total MBL-positive isolates (440). By contrast,	We thank the reviewer for this important clarification. We have addressed this comment by revising the text in the results section so that the	Page 6, Line 168, 171-174; Figure 3

	Figure 3 shows the distribution of NDM subtypes among all MBL-positive isolates	proportions of NDM subtypes are now calculated using NDM-positive isolates (n = 407) as the denominator. This ensures consistency and accurate representation of subtype distribution in the text, while Figure 3 continues to present the distribution of NDM subtypes among all MBL-positive isolates, as intended.	
11	Lines 172-175, the percentages reported here (24.8%, 5.0%, 81.1%, and 10.7%) are inconsistent with the data presented in Figure 3. Please verify	We thank the reviewer for bringing this inconsistency to our attention. We have reviewed the data and corrected the percentages reported so that they are now fully consistent with the data presented in Figure 3	Page 7, Line 176-181<br>Page 6, Line 171-173
12	Line 182, the MIC_{90} value listed in Table 2 is 0.25 mg/L, which is inconsistent with the value of 0.12 mg/L shown here	We thank the reviewer for bringing this inconsistency to our attention. We have reviewed the data and corrected the values so that they are now fully consistent with the data presented in Table 2.	Page 7, Line 187-188
13	Line 198, these values are inconsistent with those in Supplementary Table 7, where the MIC_{50} and MIC_{90} are 0.06 mg/L and 0.12 mg/L, respectively	We thank the reviewer for bringing this inconsistency to our attention. We have	Page 7-8, Line 204-209

		reviewed the data and corrected the values so that they are now fully consistent with the data presented in Supplementary Table 7. *In addition to addressing specific reviewer comments, all MIC_{50}/MIC_{90} values, susceptibility percentages, and cumulative inhibition data in the main text, tables, and supplementary materials have been systematically re-verified against the source ATLAS dataset to ensure accuracy and consistency.*	
14	Line199, supplementary Table 7 shows colistin at 99.3%	We thank the reviewer for bringing this inconsistency to our attention. We have reviewed the data and corrected the values so that they are now fully consistent with the data presented in Supplementary Table 7.	Page 8, Line 208
15	Line 201, "0.12/0.5 mg/L", this writing is not formal	We thank the reviewer for this comment. We have revised the notation to use more formal and standard scientific wording, replacing	Page 8, Line 210

		"0.12/0.5 mg/L" with "0.12 and 0.5 mg/L'.	
16	Line 229, "36.7%", What does this value mean?	We thank the reviewer for requesting clarification. We have revised the text to explicitly define this value and now state that **only 36.7% of MBL-positive *Escherichia coli* isolates were susceptible to cefiderocol according to EUCAST breakpoints**. This revision ensures the meaning of the percentage is clear and unambiguous.	Page 9, Line 238-239
17	Line 266, in Table 13, the cumulative inhibition rate at MIC = 4 for ATM-AVI should be 97.8% instead of 97.7%.	We thank the reviewer for this helpful suggestion. We have addressed this comment by updating the data in Table 13	Table 13
18	Line 288, China-->Chinese?	We thank the reviewer for this suggestion. We have revised the text in the manuscript by replacing **"China"** with **"Chinese"**, as recommended	Page 11, Line 318
19	Line 308, what's the exact number of NDM-1 and NDM-5	We thank the reviewer for this comment. We have revised the text to explicitly state the exact numbers, specifying	Page 12, Line 336

		**NDM-1 (n = 14)** and **NDM-5 (n = 7)**.	

Re: Spectrum00241-26R1 (In vitro activity of aztreonam-avibactam and comparators against Enterobacterales isolated from bloodstream infections: ATLAS surveillance, 2021-2022)

Dear Dr. Katherine K. Perez:

Your manuscript has been accepted, and I am forwarding it to the ASM production staff for publication. Your paper will first be checked to make sure all elements meet the technical requirements. ASM staff will contact you if anything needs to be revised before copyediting and production can begin. Otherwise, you will be notified when your proofs are ready to be viewed.

Data Availability: ASM policy requires that data be available to the public upon online posting of the article, so please verify all links to sequence records, if present, and make sure that each number retrieves the full record of the data. If a new accession number is not linked or a link is broken, provide production staff with the correct URL for the record. If the accession numbers for new data are not publicly accessible before the expected online posting of the article, publication may be delayed; please contact ASM production staff immediately with the expected release date.

Publication Fees: For information on publication fees and which article types have charges, please visit our website. We have partnered with Copyright Clearance Center (CCC) to collect author charges. If fees apply to your paper, you will receive a message from no-reply@copyright.com with further instructions. For questions related to paying charges through RightsLink, please contact CCC at ASM_Support@copyright.com or toll free at +1-877-622-5543. CCC makes every attempt to respond to all emails within 24 hours.

ASM Membership: Corresponding authors may join or renew ASM membership to obtain discounts on publication fees. Need to upgrade your membership level? Please contact Customer Service at Service@asmusa.org.

Embargo Policy: A press release may be issued as soon as the manuscript is posted on the Spectrum Latest Articles webpage. The corresponding author will receive an email with the subject line "ASM Journals Author Services Notification" when the article is available online.

The ASM Journals program strives for constant improvement in our submission and publication process. Please tell us how we can improve your experience by taking this quick Author Survey.

Thank you for submitting your paper to Spectrum.

Sincerely,
Krisztina Papp-Wallace
Editor
Microbiology Spectrum
