# Supplementary material for: In vitro activity of aztreonam-avibactam and comparators against Enterobacterales isolated from bloodstream infections: ATLAS/ surveillance, 2021–2022
Source: Microbiol Spectr. 2026 Jun 30;14(8):e00241-26. doi: 10.1128/spectrum.00241-26 (PMC13436376; doi:10.1128/spectrum.00241-26)
Supplement: Supplementary file 2 [file spectrum.00241-26-s0001.docx]

**Supplementary Table 1:** Distribution of *Enterobacterales* isolates collected from bloodstream infections overall and across different regions stratified by collection wards, 2021–2022

|  | **Overall*** | **AfME** | **APAC** | **Europe** | **Latin America** |
| --- | --- | --- | --- | --- | --- |
| **All *Enterobacterales* (N)^†^** | 10,376 | 1034 | 2431 | 5253 | 1658 |
| ICU [n (% of N)] | 2511 (24.2) | 330 (31.9) | 555 (22.8) | 1019 (19.4) | 607 (36.6) |
| Non-ICU^‡^ [n (% of N)] | 7423 (71.5) | 634 (61.3) | 1792 (73.7) | 4006 (76.3) | 991 (59.8) |
| **All MDR (EUCAST) [n (% of total *Enterobacterales*)]** | 4604 (44.4) | 669 (64.7) | 1186 (48.8) | 1862 (35.4) | 887 (53.5) |
| ICU [n (% of total MDR)] | 1396 (30.3) | 239 (35.7) | 348 (29.3) | 452 (24.3) | 357 (40.2) |
| Non-ICU^‡^ [n (% of total MDR)] | 2995 (65.1) | 382 (57.1) | 798 (67.3) | 1326 (71.2) | 489 (55.1) |
| **All CRE (EUCAST) [n (% of total *Enterobacterales*)]** | 710 (6.8) | 106 (10.3) | 223 (9.2) | 200 (3.8) | 181 (10.9) |
| ICU [n (% of total CRE)] | 344 (48.5) | 53 (50.0) | 122 (54.7) | 84 (42.0) | 85 (47.0) |
| Non-ICU^‡^ [n (% of total CRE)] | 342 (48.2) | 47 (44.3) | 94 (42.2) | 113 (56.5) | 88 (48.6) |
| **All ESBL screen positive (N=7997)^§^** | 1447 | 253 | 314 | 518 | 362 |
| ICU [n (% of total ESBL screen positive)] | 534 (36.9) | 100 (39.5) | 131 (41.7) | 145 (28.0) | 158 (43.6) |
| Non-ICU^‡^ [n (% of total ESBL screen positive)] | 844 (58.3) | 135 (53.4) | 178 (56.7) | 349 (67.4) | 182 (50.3) |

*Overall includes isolates from AfME, APAC, Europe and Latin America

**^†^***Enterobacterales* included the following genera: *Escherichia*, *Citrobacter*, *Enterobacter*, *Klebsiella*, *Proteus*, *Providencia*, *Morganella* and *Serratia*

^‡^Does not include isolates from wards/infection sources for which information was not specified or available

**^§^***Enterobacterales* that qualified for β-lactamase screening: *Escherichia coli, Klebsiella oxytoca, Klebsiella pneumoniae, Klebsiella variicola, Proteus mirabilis*

AfME, Africa and Middle East; APAC, Asia Pacific; CRE, Carbapenemase-resistance *Enterobacterales*; ESBL, Extended-spectrum β-lactamase; EUCAST, European Committee on Antimicrobial Susceptibility Testing; ICU, Intensive care unit; MDR, Multi-drug resistant

**Supplementary Table 2:** Distribution of different species of *Enterobacterales* isolates collected from bloodstream infections overall and across different regions from 2021–2022

|  | **Overall*** | **AfME** | **APAC** | **Europe** | **Latin America** |
| --- | --- | --- | --- | --- | --- |
| **All *Enterobacterales* (N)†** | **10,376** | **1034** | **2431** | **5253** | **1658** |
| ***Citrobacter freundii* [n (%)]** | **97 (0.9)** | **4 (0.4)** | **23 (0.9)** | **57 (1.1)** | **13 (0.8)** |
| **MDR (EUCAST) [n (%)]** | 37 (38.1) | 2 (50.0) | 9 (39.1) | 20 (35.1) | 6 (46.2) |
| **CRE (EUCAST) [n (%)]** | 3 (3.1) | 0 (0.0) | 0 (0.0) | 1 (1.8) | 2 (15.4) |
| ***Citrobacter koseri* [n (%)]** | **138 (1.3)** | **10 (1.0)** | **23 (0.9)** | **96 (1.8)** | **9 (0.5)** |
| **MDR (EUCAST) [n (%)]** | 10 (7.2) | 0 (0.0) | 3 (13.0) | 6 (6.3) | 1 (11.1) |
| **CRE (EUCAST) [n (%)]** | 0 | 0 | 0 | 0 | 0 |
| **Other *Citrobacter spp* [n (%)]^‡^** | **48 (0.5)** | **7 (0.7)** | **17 (0.7)** | **20 (0.4)** | **4 (0.2)** |
| **MDR (EUCAST) [n (%)]** | 18 (37.5) | 5 (71.4) | 7 (41.2) | 5 (25.0) | 1 (25.0) |
| **CRE (EUCAST) [n (%)]** | 1 (2.1) | 1 (14.3) | 0 (0.0) | 0 (0.0) | 0 (0.0) |
| ***Enterobacter cloacae* [n (% )]** | **238 (2.3)** | **30 (2.9)** | **47 (1.9)** | **119 (2.3)** | **42 (2.5)** |
| **MDR (EUCAST) [n (%)]** | 86 (36.1) | 19 (63.3) | 17 (36.2) | 39 (32.8) | 11 (26.2) |
| **CRE (EUCAST) [n (%)]** | 3 (1.3) | 1 (3.3) | 0 (0.0) | 0 (0.0) | 2 (4.8) |
| **Other *Enterobacter spp* [n (%)]^§^** | **739 (7.1)** | **85 (8.2)** | **145 (6.0)** | **376 (7.2)** | **133 (8.0)** |
| **MDR (EUCAST) [n (%)]** | 345 (46.7) | 49 (57.6) | 77 (53.1) | 164 (43.6) | 55 (41.4) |
| **CRE (EUCAST) [n (%)]** | 18 (2.4) | 3 (3.5) | 8 (5.5) | 1 (0.3) | 6 (4.5) |
| ***Escherichia coli* [n (%)** | **3842 (37.0)** | **291 (28.1)** | **1013 (41.7)** | **2030 (38.6)** | **508 (30.6)** |
| **MDR (EUCAST) [n (%)]** | 1731 (45.1) | 168 (57.7) | 615 (60.7) | 663 (32.7) | 285 (56.1) |
| **CRE (EUCAST) [n (%)]** | 45 (1.2) | 0 (0.0) | 33 (3.3) | 2 (0.1) | 10 (2.0) |
| **ESBL** **screen positive [n (%)]** | 472 (12.3) | 51 (17.5%) | 139 (13.7) | 174 (8.6) | 108 (21.3) |
| ***Escherichia spp* [n (%)]** | **16 (0.2)** | **1 (0.1)** | **6 (0.2)** | **8 (0.2)** | **1 (0.1)** |
| ***Klebsiella oxytoca* [n (%)]** | **374 (3.6)** | **17 (1.6)** | **41 (1.7)** | **244 (4.6)** | **72 (4.3)** |
| **MDR (EUCAST) [n (%)]** | 73 (19.5) | 4 (23.5) | 7 (17.1) | 37 (15.2) | 25 (34.7) |
| **CRE (EUCAST) [n (%)]** | 5 (1.3) | 0 (0.0) | 1 (2.4) | 0 (0.0) | 4 (5.6) |
| **ESBL** **screen positive [n (%)]** | 12 (3.2) | 1 (5.9) | 1 (2.4) | 2 (0.8) | 8 (11.1) |
| ***Klebsiella pneumoniae [*n (%)]** | **3309 (31.9)** | **473 (45.7)** | **773 (31.8)** | **1475 (28.1)** | **588 (35.5)** |
| **MDR (EUCAST) [n (%)]** | 1815 (54.9) | 371 (78.4) | 354 (45.8) | 698 (47.3) | 392 (66.7) |
| **CRE (EUCAST) [n (%)]** | 594 (18.0) | 93 (19.7) | 176 (22.8) | 189 (12.8) | 136 (23.1) |
| **ESBL screen positive [n (%)]** | 948 (28.6) | 199 (42.1) | 171 (22.1) | 333 (22.6) | 245 (41.7) |
| **Other *Klebsiella spp* [n (%)]^\|\|^** | **453 (4.4)** | **26 (2.5)** | **93 (3.8)** | **268 (5.1)** | **66 (4.0)** |
| **MDR (EUCAST) [n (%)]** | 122 (26.9) | 10 (38.5) | 18 (19.4) | 73 (27.2) | 21 (31.8) |
| **CRE (EUCAST) [n (%)]** | 13 (2.9) | 4 (15.4) | 2 (2.2) | 3 (1.1) | 4 (6.1) |
| **All *Morganella spp* [n (%)]^¶^** | **189 (1.8)** | **18 (1.7)** | **41 (1.7)** | **97 (1.8)** | **33 (2.0)** |
| **MDR (EUCAST) [n (%)]** | 56 (29.6) | 6 (33.3) | 9 (22.0) | 29 (29.9) | 12 (36.4) |
| **CRE (EUCAST) [n (%)]** | 1 (0.5) | 0 (0.0) | 0 (0.0) | 0 (0.0) | 1 (3.0) |
| ***Proteus mirabilis* [n {%)]** | **279 (2.7)** | **20 (1.9)** | **65 (2.7)** | **154 (2.9)** | **40 (2.4)** |
| **MDR (EUCAST) [n (%)]** | 94 (33.7) | 8 (40.0) | 22 (33.8) | 50 (32.5) | 14 (35.0) |
| **CRE (EUCAST) [n (%)]** | 3 (1.1) | 1 (5.0) | 0 (0.0) | 1 (0.6) | 1 (2.5) |
| **ESBL** **screen positive [n (%)]** | 4 (1.4) | 1 (5.0) | 2 (3.1) | 0 (0.0) | 1 (2.5) |
| **Other *Proteus spp* [n (%)]**** | **60 (0.6)** | **2 (0.2)** | **11 (0.5)** | **42 (0.8)** | **5 (0.3)** |
| **MDR (EUCAST) [n (%)]** | 15 (25.0) | 1 (50.0) | 6 (54.5) | 6 (14.3) | 2 (40.0) |
| **All *Providencia spp* [n (%)]^††^** | **110 (1.1)** | **10 (1.0)** | **31 (1.3)** | **54 (1.0)** | **15 (0.9)** |
| **MDR (EUCAST) [n (%)]** | 52 (47.3) | 5 (50.0) | 13 (41.9) | 20 (37.0) | 14 (93.3) |
| **CRE (EUCAST) [n (%)]** | 7 (6.4) | 1 (10.0) | 1 (3.2) | 1 (1.9) | 4 (26.7) |
| ***Serratia marcescens* [n (%)]** | **387 (3.7)** | **29 (2.8)** | **83 (3.4)** | **167 (3.2)** | **108 (6.5)** |
| **MDR (EUCAST) [n (%)]** | 58 (15.0) | 10 (34.5) | 13 (15.7) | 8 (4.8) | 27 (25.0) |
| **CRE (EUCAST) [n (%)]** | 13 (3.4) | 1 (3.4) | 2 (2.4) | 2 (1.2) | 8 (7.4) |
| **Other *Serratia spp* [n (%)]^‡‡^** | **97 (0.9)** | **11 (1.1)** | **19 (0.8)** | **46 (0.9)** | **21 (1.3)** |
| **MDR (EUCAST) [n (%)]** | 89 (91.8) | 11 (100.0) | 15 (78.9) | 43 (93.5) | 20 (95.2) |
| **CRE (EUCAST) [n (%)]** | 4 (4.1) | 1 (9.1) | 0 (0.0) | 0 (0.0) | 3 (14.3) |

*Overall includes isolates from AfME, APAC, Europe and Latin America

**^†^***Enterobacterales* included the following genera: *Escherichia*, *Citrobacter*, *Enterobacter*, *Klebsiella*, *Proteus*, *Providencia*, *Morganella* and *Serratia*

**^‡^**Other *Citrobacter spp* includes- *Citrobacter amalonaticus, Citrobacter braakii, Citrobacter farmeri,, Citrobacter sedlakii*

**^§^**Other *Enterobacter spp* includes- *Enterobacter asburiae, Enterobacter bugandensis, Enterobacter hormaechi, Enterobacter kobei, Enterobacter ludwigii, Enterobacter roggenkampii*

**^||^**Other *Klebsiella spp* includes*- Klebsiella aerogenes, Klebsiella variicola*

^¶^*Morganella spp* includes- *Morganella morganii*

**Other *Proteus spp* includes - *Proteus vulgaris*

**^††^***Providencia spp includes- Providencia alcalifaciens, Providencia rettgeri, Providencia stuartii*

**^‡‡^***Other Serratia spp includes- Serratia liquefaciens, Serratia marcescens*

AfME, Africa and Middle East; APAC, Asia Pacific; CRE, Carbapenemase-resistance *Enterobacterales*; ESBL, Extended-spectrum β-lactamase; EUCAST, European Committee on Antimicrobial Susceptibility Testing; ICU, Intensive care unit; MDR, Multi-drug resistant

**Supplementary Table 3:** Distribution of NDM-positive *Enterobacterales* isolated from BSI collected overall and across regions between 2021-2022

|  | **Global** | **AfME** | **APAC** | **Europe** | **Latin America** |
| --- | --- | --- | --- | --- | --- |
| **NDM** | 407 | 95 | 160 | 57 | 95 |
| **NDM-1 [n (%)]** | 201 (49.4) | 45 (47.4) | 52 (32.5) | 50 (87.7) | 54 (56.8) |
| **NDM-5 [n (%)]** | 174 (42.8) | 39 (41.1) | 98 (61.3) | 7 (12.3) | 30 (31.6) |
| **NDM-7 [n (%)]** | 13 (3.2) | 8 (8.4) | 3 (1.9) | 0 | 2 (2.1) |
| **NDM-6 [n (%)]** | 10 (2.5) | 0 | 1 (0.6) | 0 | 9 (9.5) |
| **NDM-4 [n (%)]** | 3 (0.7) | 1 (1.1) | 2 (1.2) | 0 | 0 |
| **NDM-9 [n (%)]** | 2 (0.5) | 0 | 2 (1.2) | 0 | 0 |
| **NDM-TYPE [n (%)]** | 2 (0.5) | 0 | 2 (1.2) | 0 | 0 |
| **NDM-8 [n (%)]** | 1 (0.2) | 1 (1.1) | 0 | 0 | 0 |
| **NDM-14 [n (%)]** | 1 (0.2) | 1 (1.1) | 0 | 0 | 0 |

AfME, Africa and Middle East; APAC, Asia Pacific; NDM, New Delhi Metallo-β-lactamase

**Supplementary Table 4:** Genotypic distribution of MBL-positive *Enterobacterales* isolated from BSI between 2021-2022 among *E. coli* and *K. pneumoniae*

|  | ***E. coli*** | ***K. pneumoniae*** |
| --- | --- | --- |
| **MBL-producing *Enterobacterales* (N**) | 49 | 306 |
| NDM [n (%)] | 48 (98.0) | 291 (95.1) |
| NDM-1 [n (%)] | 4 (8.2) | 142 (46.4) |
| NDM-5 [n (%)] | 41 (83.7) | 126 (41.2) |
| Other NDM [n (%)] | 3 (6.1) | 23 (7.5) |
| VIM [n (%)] | 0 | 14 (4.6) |
| IMP [n (% )] | 1 (2.0) | 1 (0.3) |
| MBL+OXA-48 and OXA-48-like* [n (%)] | 5 (10.2) | 98 (32.0) |
| MBL+KPC [n (%)] | 0 | 17 (5.6) |
| MBL+ESBL-positive [n (%)] | 42 (85.7) | 305 (99.7) |
| MBL+AmpC-positive [n (%)] | 17 (34.7) | 26 (8.5) |

ESBL, Extended-spectrum β-lactamase; IMP, Imipenemase Metallo-β-lactamase; KPC, *Klebsiella pneumoniae* carbapenemase; MBL, Metallo- β-lactamase; NDM, New Delhi Metallo-β-lactamase; OXA-48-like, Oxacillinase-48-like β-lactamase; VIM, Verona Integron-encoded Metallo-β-lactamase

*OXA-48-like constitutes of OXA-163, OXA-181, OXA-232, and OXA-244

**Supplementary Table 5:** Antimicrobial activity of aztreonam-avibactam and comparator agents against *E. coli* and *K. pneumoniae* isolated from BSI between 2021-2022

| **Antimicrobial agent** | **Susceptibility (EUCAST)** | | **MIC (mg/L)** | | **No. of isolates inhibited at respective MIC (mg/L) (% cumulative inhibition)** | | | | | | | | | | | | | | | | | | | | | | | |
| --- | --- | --- | --- | --- | --- | --- | --- | --- | --- | --- | --- | --- | --- | --- | --- | --- | --- | --- | --- | --- | --- | --- | --- | --- | --- | --- | --- | --- |
|  | **%S** | **%R** | **MIC_50_** | **MIC_90_** | **≤0.06** | **0.12** | **0.25** | | **0.5** | | **1** | | **2** | | **4** | | | | **8** | | | **16** | | **32** | | | **>32** | |
| ***E. coli* (N=3842)** | | | | | | | | | | | | | | | | | | | | | | | | | | | | |
| **Aztreonam/avibactam** | **99.7** | **0.3** | **0.03** | **0.06** | **3459 (90.0)** | **257 (96.7)** | | **33 (97.6)** | | **21 (98.1)** | | **14 (98.5)** | | **21 (99)** | | **27 (99.7)** | | **7 (99.9)** | | | **0** | | **0** | | | **3 (100)** | | |
| Aztreonam | 69.8 | 26.8 | 0.12 | 32 | 1284 (33.4) | 1106 (62.2) | | 211 (67.7) | | 40 (68.7) | | 39 (69.8) | | 46 (70.9) | | 85 (73.2) | | 178 (77.8) | | | 211 (83.3) | | 275 (90.4) | | | 367 (100) | | |
| Cefepime | 71.6 | 22.3 | 0.12 | 64 |  | 2531 (65.9) | | 92 (68.3) | | 63 (69.9) | | 65 (71.6) | | 75 (73.6) | | 160 (77.7) | | 184 (82.5) | | | 117 (85.5) | | 167 (89.9) | | | 388 (100) | | |
| Colistin | 99.5 | 0.5 | 0.12 | 0.25 |  | 2110 (54.9) | | 1584 (96.1) | | 102 (98.8) | | 22 (99.4) | | 6 (99.5) | | 10 (99.8) | | 5 (99.9) | | | 3 (100) | |  | | |  | | |
| Meropenem | 98.6 | 1.2 | 0.06 | 0.06 | 3715 (96.7) | 36 (97.6) | | 17 (98.1) | | 6 (98.2) | | 6 (98.4) | | 8 (98.6) | | 2 (98.6) | | 7 (98.8) | | | 8 (99.0) | | **37** (100) | | |  | | |
| Tigecycline* | 99.8 | 0.03 | 0.25 | 0.5 | 125 (3.2) | 1773 (49.4) | | 1345 (84.4) | | 458 (96.3) | | 112 (99.2) | | 22 (99.8) | | 6 (99.0) | | 0 | | | 1 (100) | |  | | |  | | |
| Amikacin | 96.6 | 3.4 | 2 | 8 |  |  | | 5 (0.1) | | 24 (0.7) | | 384 (10.7) | | 2060 (64.4) | | 926 (88.5) | | 311 (96.6) | | | 77 (98.6) | | 17 (99.0) | | | 38 (100) | | |
| Ceftazidime/avibactam | 98.7 | 1.3 | 0.12 | 0.25 | 1650 (54.2) | 1571 (83.8) | | 43 (95.1) | | 77 (97.1) | | 32 (97.9) | | 23 (98.5) | | 5 (98.7) | | 0 (98.7) | | | 0 (98.7) | | 0 (98.7) | | | 51 (100) | | |
| Ceftolozane/tazobactam | 95.9 | 4.1 | 0.25 | 1 | 9 (0.2) | 430 (11.4) | | 2160 (67.6) | | 821 (89.0) | | 204 (94.3) | | 61 (95.9) | | 31 (96.7) | | 29 (97.5) | | | 17 (97.9) | | 80 (100) | | |  | | |
| Piperacillin/tazobactam | 89.6 | 10.4 | 2 | 16 |  |  | |  | | 111 (2.9) | | 848 (24.0) | | 1781 (71.3) | | 497 (84.2) | | 207 (89.6) | | | 114 (92.6) | | 70 (94.4) | | | 214 (100) | | |
| ***K. pneumoniae* (N=3309)** | | | | | | | | | | | | | | | | | | | | | | | | | | | | |
| **Aztreonam/avibactam** | **99.9** | **0.1** | **0.06** | **0.25** | **2029 (61.6)** | **657 (81.44)** | | **366 (92.5)** | | **174 (97.8)** | | **49 (99.2)** | | **14 (99.7)** | | | **8 (99.9)** | | | **2 (99.9)** | | **0** | | | **0** | | | **1 (100)** |
| Aztreonam | 50.9 | 47.5 | 1 | 128 | 1124 (34.0) | 378 (45.4) | | 99 (48.4) | | 47 (49.8) | | 37 (50.9) | | 23 (51.6) | | | 29 (52.5) | | | 31 (53.4) | | 87 (56.1) | | | 245 (63.5) | | | 1209 (100) |
| Cefepime | 51.2 | 46.1 | 1 | 64 |  | 1510 (45.4) | | 81 (47.8) | | 66 (49.8) | | 46 (51.2) | | 37 (52.3) | | | 53 (53.9) | | | 172 (59.1) | | 218 (65.7) | | | 242 (73.0) | | | 893 (100) |
| Colistin | 95.1 | 4.9 | 0.25 | 0.5 |  | 901 (27.2) | | 2014 (88.2) | | 155 (92.8) | | 53 (94.4) | | 25 (95.1) | | | 29 (96.0) | | | 40 (97.2) | | 92 (100) | | |  | | |  |
| Meropenem | 79.2 | 18 | 0.06 | 32 | 2407 (72.7) | 60 (74.5) | | 28 (75.4) | | 32 (76.4) | | 44 (77.7) | | 50 (79.2) | | | 42 (80.5) | | | 52 (82.0) | | 94 (84.9) | | | 500 (100) | | |  |
| Tigecycline* | 97.0 | 0.5 | 0.5 | 2 | 3 (0.1) | 34 (1.1) | | 738 (23.4) | | 1420 (66.3) | | 743 (88.8) | | 271 (97.0) | | | 84 (99.5) | | | 13 (99.9) | | 3 (100) | | |  | | |  |
| Amikacin | 85.2 | 14.7 | 1 | 64 |  |  | | 7 (0.2) | | 200 (6.3) | | 1462 (50.4) | | 595 (68.4) | | | 406 (80.7) | | | 151 (85.2) | | 94 (88.1) | | | 56 (89.8) | | | 338 (100) |
| Ceftazidime/avibactam | 90.1 | 9.9 | 0.25 | 8 | 256 (8.7) | 1177 (43.3) | | 708 (64.7) | | 393 (76.6) | | 244 (83.9) | | 139 (88.1) | | | 52 (89.7) | | | 12 (90.1) | | 2 (90.1) | | | 3 (90.2) | | | 323 (100) |
| Ceftolozane/tazobactam | 70.4 | 29.6 | 0.5 | 32 | 4 (0.1) | 58 (1.9) | | 882 (28.5) | | 850 (54.2) | | 355 (64.9) | | 182 (70.4) | | | 78 (72.8) | | | 57 (74.5) | | 58 (76.3) | | | 785 (100) | | |  |
| Piperacillin/tazobactam | 57.2 | 42.8 | 8 | 128 |  |  | |  | | 26 (0.8) | | 83 (3.3) | | 730 (25.4) | | | 805 (49.7) | | | 249 (57.2) | | 220 (63.9) | | | 120 (67.5) | | | 1076 (100) |

^*^ FDA breakpoints have been used

EUCAST, European Committee on Antimicrobial Susceptibility Testing; MIC, Minimum Inhibitory Concentration; MIC_50_, minimum concentration required to inhibit 50% of the organisms; MIC_90_, minimum concentration required to inhibit 90% of the organisms; S, Susceptibility; R, Resistance

**Supplementary Table 6:** Antimicrobial activity of aztreonam-avibactam and comparator agents against *Enterobacterales* isolated from BSI collected across regions from 2021-2022

| **Antimicrobial agent** | **Susceptibility (EUCAST)** | | **MIC (mg/L)** | | |
| --- | --- | --- | --- | --- | --- |
|  | **%S** | **%R** | **MIC_50_** | **MIC_90_** | **MIC range** |
| **AfME (N=1034)** |  | | | | |
| **Aztreonam/avibactam** | **99.8** | **0.2** | **0.06** | **0.25** | **0.015-128** |
| Aztreonam | 46.4 | 51.4 | 8 | 128 | 0.03- 128 |
| Cefepime | 46.9 | 47.4 | 4 | 64 | 0.12-64 |
| Colistin | 87.0^†^ | 13.0 | 0.25 | 16 | 0.12- 16 |
| Meropenem | 87.8 | 10.2 | 0.06 | 16 | 0.06- 32 |
| Tigecycline* | 97.2^†^ | 0.6 | 0.5 | 1 | 0.03-16 |
| Amikacin | 88.2 | 11.8 | 2 | 16 | 0.25-128 |
| Ceftazidime/avibactam | 89.9 | 10.1 | 0.25 | 32 | 0.03-128 |
| Ceftolozane/tazobactam | 77.8 | 22.2 | 0.5 | 32 | 0.06-32 |
| Piperacillin/tazobactam | 66.9 | 33.1 | 4 | 128 | 0.5-128 |
| **APAC (N=2431)** |  | | | | |
| **Aztreonam/avibactam** | **99.5** | **0.5** | **0.06** | **0.25** | **0.015-** **128** |
| Aztreonam | 62.6 | 33.6 | 0.12 | 128 | 0.03- 128 |
| Cefepime | 66.3 | 28.2 | 0.12 | 64 | 0.12-64 |
| Colistin | 87.0^†^ | 13.0 | 0.25 | 16 | 0.12-16 |
| Meropenem | 90.0 | 9.2 | 0.06 | 2 | 0.06-32 |
| Tigecycline* | 96.9^†^ | 0.8 | 0.5 | 2 | 0.03-16 |
| Amikacin | 91.4 | 8.6 | 2 | 8 | 0.25-128 |
| Ceftazidime/avibactam | 92.6 | 7.4 | 0.12 | 1 | 0.03-128 |
| Ceftolozane/tazobactam | 83.7 | 16.3 | 0.5 | 32 | 0.06-32 |
| Piperacillin/tazobactam | 79.0 | 21.0 | 2 | 128 | 0.5-128 |
| **Europe (N=5253)** |  | | | | |
| **Aztreonam/avibactam** | **99.9** | **0.1** | **0.06** | **0.12** | **0.015-8** |
| Aztreonam | 75.2 | 22.6 | 0.12 | 64 | 0.03-128 |
| Cefepime | 78.1 | 18.1 | 0.12 | 32 | 0.12-64 |
| Colistin | 87.5^†^ | 12.5 | 0.25 | 16 | 0.12-16 |
| Meropenem | 95.5 | 3.8 | 0.06 | 0.12 | 0.06-32 |
| Tigecycline* | 97.6^†^ | 0.2 | 0.25 | 1 | 0.03-16 |
| Amikacin | 95.9 | 4.1 | 2 | 4 | 0.25-128 |
| Ceftazidime/avibactam | 98.4 | 1.6 | 0.12 | 0.5 | 0.03-128 |
| Ceftolozane/tazobactam | 89.4 | 10.6 | 0.25 | 4 | 0.06-32 |
| Piperacillin/tazobactam | 80.4 | 19.6 | 2 | 128 | 0.5-128 |
| **Latin America**  **(N=1658)** |  | | | | |
| **Aztreonam/avibactam** | **100** | **0** | **0.06** | **0.25** | **0.015-4** |
| Aztreonam | 56.1 | 40.6 | 0.25 | 128 | 0.03-128 |
| Cefepime | 57.1 | 37.0 | 0.12 | 64 | 0.12-64 |
| Colistin | 83.6^†^ | 16.4 | 0.25 | 16 | 0.12-16 |
| Meropenem | 85.1 | 10.9 | 0.06 | 16 | 0.06-32 |
| Tigecycline* | 97.4^†^ | 0.3 | 0.5 | 1 | 0.03-8 |
| Amikacin | 88.0 | 12 | 2 | 16 | 0.25-128 |
| Ceftazidime/avibactam | 93.6 | 6.4 | 0.12 | 2 | 0.03-128 |
| Ceftolozane/tazobactam | 76.9 | 23.1 | 0.5 | 32 | 0.06-32 |
| Piperacillin/tazobactam | 69.5 | 30.5 | 4 | 128 | 0.5-128 |

^*^FDA breakpoints have been used

^†^Intrinsic resistance to colistin and tigecycline has been noted in *Proteus spp, Providencia spp., M. morganii* and *S. marcescens* and may contribute to elevated MIC values

*Enterobacterales* included the following genera: *Escherichia*, *Citrobacter*, *Enterobacter*, *Klebsiella*, *Proteus*, *Providencia*, *Morganella* and *Serratia*

AfME, Africa and Middle East; APAC, Asia Pacific; EUCAST, European Committee on Antimicrobial Susceptibility Testing; MIC, Minimum Inhibitory Concentration

**Supplementary Table 7:** Antimicrobial activity of aztreonam-avibactam and comparator agents against MDR *E. coli* and *K. pneumoniae* isolated from BSI between 2021-2022

| **Antimicrobial agent** | **Susceptibility (EUCAST)** | | **MIC (mg/L)** | | | | **No. of isolates inhibited at respective MIC (mg/L) (% cumulative inhibition)** | | | | | | | | | | | |
| --- | --- | --- | --- | --- | --- | --- | --- | --- | --- | --- | --- | --- | --- | --- | --- | --- | --- | --- |
|  | **%S** | **%R** | **MIC_50_** | **MIC_90_** | **MIC range** | **≤0.06** | | **0.12** | **0.25** | **0.5** | **1** | **2** | **4** | **8** | **16** | **32** | **>32** |  |
| ***E. coli* (N=1731)** | | | | | | | | | | | | | | | | | | |
| **Aztreonam/avibactam** | **99.4** | **0.6** | **0.06** | **0.12** | **0.015-128** | **1449 (83.7)** | | **166 (93.3)** | **26 (94.8)** | **19 (95.9)** | **14 (96.7)** | **20 (97.9)** | **27 (99.4)** | **7 (99.8)** | **0 (99.8)** | **0 (99.8)** | **3 (100)** |  |
| Aztreonam | 33.8 | 59.6 | 8 | 128 | 0.03-128 | 167 (9.6) | | 290 (26.4) | 84 (31.2) | 27 (32.8) | 17 (33.8) | 34 (35.8) | 81 (40.4) | 178 (50.7) | 211 (62.9) | 275 (78.8) | 367 (100) |  |
| Cefepime | 37.6 | 49.4 | 4 | 64 | 0.12-64 | 0 | | 472 (27.3) | 71 (31.4) | 57 (34.7) | 50 (37.6) | 70 (41.6) | 156 (50.6) | 183 (61.2) | 117 (67.9) | 167 (77.6) | 388 (100) |  |
| Colistin | 99.3 | 0.8 | 0.12 | 0.25 | 0.12-16 | 0 | | 905 (52.3) | 740 (95.0) | 58 (98.4) | 11 (99.0) | 4 (99.3) | 8 (99.7) | 2 (99.8) | 3 (100) | 0 | 0 |  |
| Meropenem | 96.9 | 2.6 | 0.06 | 0.06 | 0.06-32 | 1619 (93.5) | | 23 (94.9) | 15 (95.7) | 6 (96.1) | 6 (96.4) | 8 (96.9) | 2 (97.0) | 7 (97.4) | 8 (97.9) | 37 (100) | 0 |  |
| Tigecycline* | 99.7 | 0 | 0.25 | 0.5 | 0.03-4 | 33 (1.9) | | 627 (38.1) | 679 (77.6) | 276 (93.3) | 90 (98.5) | 20 (99.7) | 6 (100) | 0 | 0 | 0 | 0 |  |
| Amikacin | 93.1 | 6.9 | 2 | 8 | 0.5-128 | 0 | | 0 | 0 | 11 (0.6) | 124 (7.8) | 807 (54.4) | 439 (79.9) | 230 (93.1) | 65 (96.8) | 17 (97.8) | 38 (100) |  |
| ***K. pneumoniae* (N=1815)** | | | | | | | | | | | | | | | | | | |
| **Aztreonam/avibactam** | **99.9** | **0.1** | **0.12** | **0.5** | **0.015 - 128** | **721 (39.7)** | | **508 (67.7)** | **344 (86.7)** | **173 (96.2)** | **47 (98.8)** | **14 (99.7)** | **6 (99.9)** | **1 (99.9)** | **0** | **0** | **1 (100)** |  |
| Aztreonam | 11.1 | 86.5 | 128 | 128 | 0.03 - 128 | 35 (1.9) | | 55 (5.0) | 40 (7.2) | 41 (9.4) | 31 (11.1) | 20 (12.2) | 24 (13.6) | 29 (15.2) | 87 (19.9) | 244 (33.4) | 1209 (100) |  |
| Cefepime | 11.6 | 84.0 | 32 | 64 | 0.12 - 64 | 0 | | 95 (5.2) | 31 (6.9) | 50 (9.7) | 35 (11.6) | 30 (13.3) | 49 (16) | 172 (25.4) | 218 (37.5) | 242 (50.8) | 893 (100) |  |
| Colistin | 91.2 | 8.8 | 0.25 | 1 | 0.12 - 16 | 0 | | 437 (24.1) | 1073 (83.2) | 94 (88.4) | 34 (90.3) | 18 (91.2) | 29 (92.8) | 40 (95.0) | 90 (100) | 0 | 0 |  |
| Meropenem | 62.1 | 32.7 | 0.06 | 32 | 0.06 - 32 | 934 (51.5) | | 47 (54.0) | 23 (55.3) | 30 (57.0) | 43 (59.3) | 50 (62.1) | 42 (64.4) | 52 (67.3) | 94 (72.5) | 500 (100) | 0 |  |
| Tigecycline* | 95.0 | 0.9 | 0.5 | 2 | 0.03 - 16 | 2 (0.1) | | 7 (0.5) | 246 (14.1) | 672 (51.1) | 570 (82.5) | 228 (95.0) | 74 (99.1) | 13 (99.8) | 3 (100) | 0 | 0 |  |
| Amikacin | 73.2 | 26.8 | 4 | 128 | 0.25 - 128 | 0 | | 0 | 2 (0.1) | 53 (3.0) | 413 (25.8) | 324 (43.6) | 395 (65.4) | 142 (73.2) | 94 (78.4) | 55 (81.4) | 337 (100) |  |

^*^ FDA breakpoints have been used

EUCAST, European Committee on Antimicrobial Susceptibility Testing; MIC, Minimum Inhibitory Concentration; MIC_50_, minimum concentration required to inhibit 50% of the organisms; MIC_90_, minimum concentration required to inhibit 90% of the organisms; S, Susceptibility; R, Resistance

**Supplementary Table 8:** Antimicrobial activity of aztreonam-avibactam and comparator agents against MDR *Enterobacterales* isolated from BSI collected across regions from 2021-2022

| **Antimicrobial agent** | **Susceptibility (EUCAST)** | | **MIC (mg/L)** | | |
| --- | --- | --- | --- | --- | --- |
|  | **%S** | **%R** | **MIC_50_** | **MIC_90_** | **MIC range** |
| **AfME (N=669)** |  | | | | |
| **Aztreonam/avibactam** | **99.7** | **0.3** | **0.06** | **0.25** | **0.015-128** |
| Amikacin | 81.9 | 18.1 | 4 | 128 | 0.5-128 |
| Aztreonam | 18.2 | 79.4 | 32 | 128 | 0.03-128 |
| Cefepime | 18.5 | 73.2 | 16 | 64 | 0.12-64 |
| Colistin | 88.5^†^ | 11.5 | 0.25 | 8 | 0.12-16 |
| Meropenem | 81.2 | 15.8 | 0.06 | 32 | 0.06-32 |
| Tigecycline* | 96.7^†^ | 0.6 | 0.5 | 2 | 0.03-16 |
| **APAC (N=1186)** |  | | | | |
| **Aztreonam/avibactam** | **99.1** | **0.9** | **0.06** | **0.5** | **0.015-128** |
| Amikacin | 82.9 | 17.1 | 2 | 128 | 0.5-128 |
| Aztreonam | 24.4 | 68.9 | 16 | 128 | 0.03-128 |
| Cefepime | 31.9 | 57.8 | 8 | 64 | 0.12-64 |
| Colistin | 88.4^†^ | 11.6 | 0.25 | 8 | 0.12-16 |
| Meropenem | 79.5 | 18.8 | 0.06 | 32 | 0.06-32 |
| Tigecycline* | 95.7^†^ | 1.4 | 0.5 | 2 | 0.06-16 |
| **Europe (N=1862)** |  | | | | |
| **Aztreonam/avibactam** | **99.8** | **0.2** | **0.06** | **0.5** | **0.015-8** |
| Amikacin | 89.0 | 11.0 | 2 | 16 | 0.25-128 |
| Aztreonam | 31.6 | 63.6 | 32 | 128 | 0.03-128 |
| Cefepime | 38.8 | 51.0 | 8 | 64 | 0.12-64 |
| Colistin | 86.4^†^ | 13.6 | 0.25 | 16 | 0.12-16 |
| Meropenem | 87.2 | 10.7 | 0.06 | 16 | 0.06-32 |
| Tigecycline* | 96.2^†^ | 0.3 | 0.5 | 2 | 0.06-16 |
| **Latin America (N=887)** |  | | | | |
| **Aztreonam/avibactam** | **100** | **0** | **0.06** | **0.5** | **0.015-4** |
| Amikacin | 78.2 | 21.8 | 4 | 64 | 0.5-128 |
| Aztreonam | 18.9 | 75.4 | 32 | 128 | 0.03-128 |
| Cefepime | 20.9 | 69.0 | 32 | 64 | 0.12-64 |
| Colistin | 84.9^†^ | 15.1 | 0.25 | 16 | 0.12-16 |
| Meropenem | 72.1 | 20.4 | 0.06 | 32 | 0.06-32 |
| Tigecycline* | 96.5^†^ | 0.3 | 0.5 | 2 | 0.03-8 |

^*^ FDA breakpoints have been used

^†^Intrinsic resistance to colistin and tigecycline has been noted in *Proteus spp, Providencia spp., M. morganii* and *S. marcescens* and may contribute to elevated

*Enterobacterales* included the following genera: *Escherichia*, *Citrobacter*, *Enterobacter*, *Klebsiella*, *Proteus*, *Providencia*, *Morganella* and *Serratia*

AfME, Africa and Middle East; APAC, Asia Pacific; EUCAST, European Committee on Antimicrobial Susceptibility Testing; MIC, Minimum Inhibitory Concentration; MIC_50_, minimum concentration required to inhibit 50% of the organisms; MIC_90_, minimum concentration required to inhibit 90% of the organisms; S, Susceptibility; R, Resistance

**Supplementary Table 9:** Antimicrobial activity of aztreonam-avibactam and comparator agents against ESBL screen-positive *E. coli* and *K. pneumoniae* isolated from BSI between 2021-2022

| **Antimicrobial agent** | **Susceptibility (EUCAST)** | | **MIC (mg/L)** | | **No. of isolates inhibited at respective MIC (mg/L) (% cumulative inhibition)** | | | | | | | | | | |
| --- | --- | --- | --- | --- | --- | --- | --- | --- | --- | --- | --- | --- | --- | --- | --- |
|  | **%S** | **%R** | **MIC_50_** | **MIC_90_** | **≤0.06** | **0.12** | **0.25** | **0.5** | **1** | **2** | **4** | **8** | **16** | **32** | **>32** |
| ***E. coli* (N=472)** | | | | | | | | | | | | | | | |
| **Aztreonam/ avibactam** | **99.8** | **0.2** | **0.06** | **0.12** | 392 (83.0) | 48 (93.2) | 5 (94.2) | 8 (95.9) | 6 (97.2) | 7 (98.7) | 5 (99.7) | 0 (99.7) | 0 (99.7) | 0 (99.7) | 1 (100) |
| Aztreonam | 0.4 | 91.5 | 32 | 128 | 0 | 0 | 1 (0.2) | 0 (0.2) | 1 (0.4) | 7 (1.9) | 31 (8.4) | 74 (24.1) | 89 (43.0) | 120 (68.4) | 149 (100) |
| Cefepime | 2.8 | 78.4 | 32 | 64 | 0 | 3 (0.6) | 0 (0.6) | 2 (1.0) | 8 (2.7) | 20 (6.9) | 69 (21.6) | 66 (35.5) | 51 (46.4) | 85 (64.4) | 168 (100) |
| Colistin | 99.6 | 0.4 | 0.12 | 0.25 | 0 | 258 (54.6) | 199 (96.8) | 7 (98.3) | 4 (99.1) | 2 (99.5) | 1 (99.7) | 0 (99.7) | 1 (100) | 0 | 0 |
| Meropenem | 94.3 | 5.1 | 0.06 | 0.06 | 431 (91.3) | 6 (92.5) | 4 (93.4) | 0 (93.4) | 2 (93.8) | 2 (94.2) | 1 (94.4) | 2 (94.9) | 5 (95.9) | 19 (100) | 0 |
| Tigecycline* | 99.6 | 0 | 0.25 | 0.5 | 8 (1.6) | 185 (40.8) | 203 (83.9) | 58 (96.1) | 13 (98.9) | 3 (99.6) | 2 (100) | 0 | 0 | 0 | 0 |
| Amikacin | 91.3 | 8.7 | 4 | 8 | 0 | 0 | 0 | 1 (0.2) | 31 (6.7) | 198 (48.7) | 116 (73.3) | 85 (91.3) | 17 (94.9) | 9 (96.8) | 15 (100) |
| ***K. pneumoniae* (N=948)** | | | | | | | | | | | | | | | |
| **Aztreonam/ avibactam** | **100** | 0 | 0.12 | 0.5 | 314 (33.1) | 253 (59.8) | 248 (85.9) | 109 (97.4) | 16 (99.1) | 5 (99.6) | 3 (100) | 0 | 0 | 0 | 0 |
| Aztreonam | 0.3 | 99.1 | 128 | 128 | 0 | 0 | 0 | 0 | 3 (0.3) | 5 (0.8) | 1 (0.9) | 10 (2) | 40 (6.2) | 131 (20.0) | 758 (100) |
| Cefepime | 1.0 | 96.4 | 64 | 64 | 0 | 1 (0.1) | 2 (0.3) | 1 (0.4) | 5 (0.9) | 8 (1.7) | 17 (3.5) | 69 (10.8) | 121 (23.6) | 126 (36.9) | 598 (100) |
| Colistin | 87.5 | 12.6 | 0.25 | 8 | 0 | 213 (22.4) | 537 (79.1) | 44 (83.7) | 21 (85.9) | 14 (87.4) | 20 (89.5) | 32 (92.9) | 67 (100) | 0 | 0 |
| Meropenem | 45.9 | 45.9 | 8 | 32 | 348 (36.7) | 14 (38.1) | 8 (39.0) | 7 (39.7) | 17 (41.5) | 41 (45.8) | 34 (49.4) | 44 (54.1) | 71 (61.6) | 364(100) | 0 |
| Tigecycline* | 95.6 | 0.7 | 1 | 2 | 0 | 6 (0.6) | 117 (12.9) | 335 (48.3) | 321 (82.1) | 124 (95.2) | 38 (99.2) | 6 (99.8) | 0(99.8) | 1(100) | 0 |
| Amikacin | 64.2 | 35.8 | 4 | 128 | 0 | 0 | 0 | 20 (2.1) | 152 (18.1) | 147 (33.6) | 214 (56.2) | 76 (64.2) | 60 (70.5) | 33 (74.0) | 246 (100) |

^*^ FDA breakpoint have been used

EUCAST, European Committee on Antimicrobial Susceptibility Testing; MIC, Minimum Inhibitory Concentration; MIC_50_, minimum concentration required to inhibit 50% of the organisms; MIC_90_, minimum concentration required to inhibit 90% of the organisms; S, Susceptibility; R, Resistance

**Supplementary Table 10:** Antimicrobial activity of aztreonam-avibactam and comparator agents against ESBL screen positive *Enterobacterales* isolated from BSI collected across regions from 2021-2022

| **Antimicrobial agent** | **Susceptibility (EUCAST)** | | **MIC (mg/L)** | | |
| --- | --- | --- | --- | --- | --- |
|  | **%S** | **%R** | **MIC_50_** | **MIC_90_** | **MIC range** |
| **AfME (N=253)** |  | | | | |
| **Aztreonam/avibactam** | **100** | **0** | **0.06** | **0.25** | **0.015-2** |
| Amikacin | 70.4 | 29.6 | 4 | 128 | 0.5-128 |
| Aztreonam | 0.4 | 98.0 | 64 | 128 | 0.25-128 |
| Cefepime | 1.2 | 93.3 | 64 | 64 | 0.12-64 |
| Colistin | 95.3^†^ | 4.7 | 0.25 | 0.25 | 0.12-16 |
| Meropenem | 61.7 | 32.0 | 0.06 | 32 | 0.06-32 |
| Tigecycline* | 96.8^†^ | 0.4 | 0.5 | 2 | 0.12-16 |
| **APAC (N=314)** |  | | | | |
| **Aztreonam/avibactam** | **99.7** | **0.3** | **0.12** | **0.5** | **0.015-128** |
| Amikacin | 65.9 | 34.1 | 4 | 128 | 0.5-128 |
| Aztreonam | 0.3 | 95.5 | 64 | 128 | 1-128 |
| Cefepime | 1.3 | 89.8 | 64 | 64 | 0.12-64 |
| Colistin | 92.7^†^ | 7.3 | 0.25 | 0.5 | 0.12-16 |
| Meropenem | 51.9 | 46.2 | 0.5 | 32 | 0.06-32 |
| Tigecycline* | 96.8^†^ | 0.3 | 0.5 | 2 | 0.06-8 |
| **Europe (N=518)** |  | | | | |
| **Aztreonam/avibactam** | **100** | **0** | **0.06** | **0.25** | **0.015-4** |
| Amikacin | 81.7 | 18.3 | 4 | 32 | 0.25-128 |
| Aztreonam | 0.4 | 96.3 | 64 | 128 | 1-128 |
| Cefepime | 1.9 | 88.2 | 32 | 64 | 0.12-64 |
| Colistin | 90.2^†^ | 9.9 | 0.25 | 2 | 0.12-16 |
| Meropenem | 72.2 | 23.8 | 0.06 | 32 | 0.06-32 |
| Tigecycline* | 96.3^†^ | 0.4 | 0.5 | 2 | 0.06-8 |
| **Latin America (N=362)** |  | | | | |
| **Aztreonam/avibactam** | **100** | **0** | **0.12** | **0.5** | **0.015-4** |
| Amikacin | 68.0 | 32.0 | 4 | 128 | 0.5-128 |
| Aztreonam | 0.3 | 97.0 | 128 | 128 | 1-128 |
| Cefepime | 1.7 | 91.7 | 64 | 64 | 0.5-64 |
| Colistin | 89.2^†^ | 10.8 | 0.25 | 4 | 0.12-16 |
| Meropenem | 56.6 | 32.3 | 0.25 | 32 | 0.06-32 |
| Tigecycline* | 96.4^†^ | 0.8 | 0.5 | 2 | 0.06-8 |

^*^ FDA breakpoint have been used

^†^Intrinsic resistance to colistin and tigecycline has been noted in *Proteus spp, Providencia spp., M. morganii* and *S. marcescens* and may contribute to elevated

Includes *Escherichia coli, Klebsiella oxytoca, Klebsiella pneumoniae, Klebsiella* *variicola, Proteus mirabilis*

AfME, Africa and Middle East; APAC, Asia Pacific; EUCAST, European Committee on Antimicrobial Susceptibility Testing; MIC, Minimum Inhibitory Concentration; MIC_50_, minimum concentration required to inhibit 50% of the organisms; MIC_90_, minimum concentration required to inhibit 90% of the organisms; S, Susceptibility; R, Resistance

**Supplementary Table 11:** Antimicrobial activity of aztreonam-avibactam and comparator agents against CRE *E. coli* and *K. pneumoniae* isolated from BSI between 2021-2022

| **Antimicrobial agent** | **Susceptibility (EUCAST)** | | **MIC (mg/L)** | | **No. of isolates inhibited at respective MIC (mg/L) (% cumulative inhibition)** | | | | | | | | | | | |
| --- | --- | --- | --- | --- | --- | --- | --- | --- | --- | --- | --- | --- | --- | --- | --- | --- |
|  | **%S** | **%R** | **MIC_50_** | **MIC_90_** | | **≤0.06** | **0.12** | **0.25** | **0.5** | **1** | **2** | **4** | **8** | **16** | **32** | **>32** |
| ***E. coli* (N=45)** | | | | | | | | | | | | | | | | |
| **Aztreonam/ avibactam** | **82.2** | 17.8 | **2** | **8** | | 5 (13.3) | 3 (17.7) | 2 (22.2) | 5 (33.3) | 5 (44.4) | 4 (53.3) | 13 (82.2) | 5 (93.3) | 0 (93.3) | 0 (93.3) | 3 (100) |
| Aztreonam | 4.4 | 95.6 | 128 | 128 | | 0 | 0 | 1 (2.2) | 1 (4.4) | 0 (4.4) | 0 | 0 | 0 | 1 (6.6) | 6 (20.0) | 36 (42.2) |
| Cefepime | 0 | 100 | 64 | 64 | | 0 | 0 | 0 | 0 | 0 | 0 | 0 | 0 | 0 | 1 (2.2) | 44 (100) |
| Colistin | 100 | 0 | 0.12 | 0.25 | | 0 | 29 (64.4) | 15 (97.7) | 1 (100) | 0 | 0 | 0 | 0 | 0 | 0 | 0 |
| Meropenem | 0 | 100 | 32 | 32 | | 0 | 0 | 0 | 0 | 0 | 0 | 0 | 0 | 8 (17.7) | 37 (100) | 0 |
| Tigecycline* | 77.8 | 22.2 | 0.25 | 1 | | 0 | 11 (24.4) | 13 (53.3) | 11 (77.7) | 6 (91.1) | 3 (97.7) | 1 (100) | 0 | 0 | 0 | 0 |
| Amikacin | 62.2 | 37.8 | 8 | 128 | | 0 | 0 | 0 | 0 | 1 (2.2) | 11 (26.6) | 5 (37.7) | 11 (62.2) | 0 | 0 | 17 (100) |
| ***K. pneumoniae* (N=594)** | | | | | | | | | | | | | | | | |
| **Aztreonam/ avibactam** | **100** | **0** | **0.25** | **0.5** | | **39 (9.6)** | **122 (27.0)** | **242 (67.8)** | **146 (92.4)** | **35 (98.3)** | **8 (99.7)** | **2 (100%)** | **0** | **0** | **0** | **0** |
| Aztreonam | 4.7 | 94.8 | 128 | 128 | | 4 (0.7) | 3 (1.2) | 6 (2.2) | 11 (4.0) | 4 (4.8) | 1 (4.9) | 2 (5.2) | 2 (5.6) | 2 (5.9) | 10 (7.6) | 549 (100) |
| Cefepime | 0.2 | 99.3 | 64 | 64 | | 0 | 0 | 0 | 0 | 1 (0.2) | 1 (0.3) | 2 (0.7) | 6 (1.7) | 7 (2.9) | 38 (9.3) | 539 (100) |
| Colistin | 81.1 | 18.9 | 0.25 | 16 | | 0 | 138 (23.2) | 281 (70.5) | 29 (75.4) | 20 (78.8) | 14 (81.1) | 19 (84.3) | 32 (89.7) | 61 (100) | 0 | 0 |
| Meropenem | 0 | 100 | 32 | 32 | | 0 | 0 | 0 | 0 | 0 | 0 | 0 | 0 | 94 (15.8) | 500 (100) | 0 |
| Tigecycline* | 93.1 | 1.4 | 1 | 2 | | 0 | 2 (0.3) | 35 (6.2) | 165 (34.0) | 228 (72.4) | 123 (93.1) | 33 (98.6) | 7 (99.8) | 1 (100) | 0 | 0 |
| Amikacin | 39.9 | 60.1 | 32 | 128 | | 0 | 0 | 0 | 4 (0.7) | 57 (10.3) | 45 (17.8) | 79 (31.1) | 52 (39.9) | 53 (48.8) | 38 (55.2) | 266 (100) |

^*^ FDA breakpoints have been used

EUCAST, European Committee on Antimicrobial Susceptibility Testing; MIC, Minimum Inhibitory Concentration; MIC_50_, minimum concentration required to inhibit 50% of the organisms; MIC_90_, minimum concentration required to inhibit 90% of the organisms; S, Susceptibility; R, Resistance

**Supplementary Table 12:** Antimicrobial activity of aztreonam-avibactam and comparator agents against CRE *Enterobacterales* isolated from BSI collected across regions from 2021-2022

| **Antimicrobial agent** | **Susceptibility (EUCAST)** | | **MIC (mg/L)** | | |
| --- | --- | --- | --- | --- | --- |
|  | **%S** | **%R** | **MIC_50_** | **MIC_90_** | **MIC range** |
| **AfME (N=106)** |  | | | | |
| **Aztreonam/avibactam** | **100** | **0** | **0.25** | **0.25** | **0.015-1** |
| Amikacin | 41.5 | 58.5 | 16 | 128 | 1-128 |
| Aztreonam | 10.4 | 88.7 | 128 | 128 | 0.06-128 |
| Cefepime | 0.94 | 98.1 | 64 | 64 | 1-64 |
| Colistin | 84.0^†^ | 16.0 | 0.25 | 16 | 0.12-16 |
| Meropenem | 0 | 100 | 32 | 32 | 16-32 |
| Tigecycline* | 91.5^†^ | 1.2 | 1 | 2 | 0.25-16 |
| **APAC (N=223)** |  | | | | |
| **Aztreonam/avibactam** | **96.9** | **3.1** | **0.25** | **2** | **0.015-128** |
| Amikacin | 39.0 | 61.0 | 128 | 128 | 0.5-128 |
| Aztreonam | 5.4 | 94.6 | 128 | 128 | 0.06-128 |
| Cefepime | 0 | 99.1 | 64 | 64 | 4-64 |
| Colistin | 88.8^†^ | 11.2 | 0.25 | 4 | 0.12-16 |
| Meropenem | 0 | 100 | 32 | 32 | 16-32 |
| Tigecycline* | 90.6^†^ | 2.7 | 1 | 2 | 0.12-8 |
| **Europe (N=200)** |  | | | | |
| **Aztreonam/avibactam** | **99.5** | **0.5** | **0.25** | **1** | **0.015-8** |
| Amikacin | 40.0 | 60.0 | 16 | 128 | 0.5-128 |
| Aztreonam | 3.0 | 95.5 | 128 | 128 | 0.12-128 |
| Cefepime | 0 | 99.5 | 64 | 64 | 2-64 |
| Colistin | 73.0^†^ | 27.0 | 0.25 | 16 | 0.12-16 |
| Meropenem | 0 | 100 | 32 | 32 | 16-32 |
| Tigecycline* | 90.5^†^ | 0 | 1 | 2 | 0.12-4 |
| **Latin America (N=181)** |  | | | | |
| **Aztreonam/avibactam** | **100** | **0** | **0.25** | **0.5** | **0.015-4** |
| Amikacin | 52.5 | 47.5 | 8 | 128 | 1-128 |
| Aztreonam | 6.1 | 92.3 | 128 | 128 | 0.03-128 |
| Cefepime | 0 | 100 | 64 | 64 | 16-64 |
| Colistin | 75.1^†^ | 24.9 | 0.25 | 16 | 0.12-16 |
| Meropenem | 0 | 100 | 32 | 32 | 16-32 |
| Tigecycline* | 93.4^†^ | 0.6 | 1 | 2 | 0.12-8 |

^*^FDA breakpoints have been used

^†^Intrinsic resistance to colistin and tigecycline has been noted in *Proteus spp, Providencia spp., M. morganii* and *S. marcescens* and may contribute to elevated

*Enterobacterales* included the following genera: *Escherichia*, *Citrobacter*, *Enterobacter*, *Klebsiella*, *Proteus*, *Providencia*, *Morganella* and *Serratia*

AfME, Africa and Middle East; APAC, Asia Pacific; EUCAST, European Committee on Antimicrobial Susceptibility Testing; MIC, Minimum Inhibitory Concentration; MIC_50_, minimum concentration required to inhibit 50% of the organisms; MIC_90_, minimum concentration required to inhibit 90% of the organisms; S, Susceptibility; R, Resistance

**Supplementary Table 13:** Antimicrobial activity of aztreonam-avibactam and comparator agents against MBL-positive *E. coli* and *K. pneumoniae* isolated from BSI between 2021-2022

| **Antimicrobial agent** | **Susceptibility (EUCAST)** | | **No. of isolates inhibited at respective MIC (mg/L) (% cumulative inhibition)** | | | | | | | | | | |
| --- | --- | --- | --- | --- | --- | --- | --- | --- | --- | --- | --- | --- | --- |
|  | **%S** | **%R** | **≤0.06** | **0.12** | **0.25** | **0.5** | **1** | **2** | **4** | **8** | **16** | **32** | **>32** |
| ***E. coli* (N= 49)** | | | | | | | | | | | | | |
| **Aztreonam/**  **avibactam** | **75.5** | **24.5** | **9 (18.4)** | **1 (20.4)** | **2 (24.5)** | **0 (24.5)** | **7 (38.8)** | **6 (51.0)** | **12 (75.5)** | **6 (87.8)** | **4 (95.9)** | **0 (95.9)** | **2 (100)** |
| Aztreonam | 8.2 | 91.8 | 0 | 1 (2.0) | 3 (8.2) | 0 (8.2) | 0 (8.2) | 0 (8.2) | 0 (8.2) | 3 (14.3) | 1 (16.3) | 6 (28.6) | 35 (100) |
| Cefepime | 0 | 100 | 0 | 0 | 0 | 0 | 0 | 0 | 0 | 1 (2.0) | 0 (2.0) | 3 (8.2) | 45 (100) |
| Cefiderocol | 36.7 | 63.3 | 0 | 0 | 0 | 1 (2.0) | 1 (4.1) | 16 (36.7) | 13 (63.3) | 6 (75.5) | 1 (77.5) | 0 (77.5) | 11 (100) |
| Colistin | 100 | 0 | 0 | 5 (10.2) | 37 (85.7) | 6 (98) | 1 (100) | 0 | 0 | 0 | 0 | 0 | 0 |
| Ertapenem | 0 | 100 | 0 | 0 | 0 | 0 | 0 | 1 (2.0) | 0 (2.0) | 1 (4.1) | 47 (100) | 0 | 0 |
| Tigecycline* | 100 | 0 | 1 (2.0) | 19 (40.8) | 19 (79.6) | 7 (93.9) | 3 (100) | 0 | 0 | 0 | 0 | 0 | 0 |
| Tobramycin | 26.5 | 73.5 | 0 | 0 | 2 (4.1) | 6 (16.3) | 5 (26.5) | 0 (26.5) | 0 (26.5) | 2 (30.6) | 34 (100) | 0 | 0 |
| Minocycline^†^ | 51.0 | 18.4 | 0 | 0 | 2 (4.1) | 4 (12.2) | 9 (30.6) | 2 (34.7) | 8 (51.0) | 15 (81.5) | 4 (89.8) | 5 (100) | 0 |
| ***K. pneumoniae* (N=306)** | | | | | | | | | | | | | |
| **Aztreonam/**  **avibactam** | 100 | 0 | 33 (10.7) | 56 (29.0) | 95 (60.1) | 101 (93.1) | 19 (93.3) | 2 (100) |  |  |  |  |  |
| Aztreonam | 6.9 | 91.2 | 6 (1.9) | 1 (2.2) | 7 (4.5) | 4 (5.8) | 3 (6.8) | 4 (8.1) | 2 (8.8) | 2 (9.4) | 2 (10.1) | 11 (13.7) | 264 (100) |
| Cefepime | 0.3 | 99.7 | 0 | 1 (0.3) | 0(0.3) | 0(0.3) | 0(0.3) | 0(0.3) | 0(0.3) | 0(0.3) | 3 (1.3) | 302 (100) | 0 |
| Cefiderocol | 55.9 | 44.1 | 0 | 0 | 1 (0.3) | 9 (3.2) | 38 (15.6) | 123 (55.8) | 116 (93.7) | 14 (98.3) | 2 (99.0) | 0(99.0) | 3 (100) |
| Colistin | 79.7 | 20.3 | 0 | 0 | 162 (52.9) | 68 (75.1) | 9 (78.1) | 5 (79.7) | 3 (80.7) | 17 (86.3) | 42 (100) | 0 | 0 |
| Ertapenem | 0.7 | 99.3 | 1 (0.3) | 0 (0.3) | 1 (0.6) | 0 (0.6) | 0 (0.6) | 1 (0.9) | 2 (1.6) | 1 (2) | 300 (100) | 0 | 0 |
| Tigecycline* | 98.0 | 0.4 | 0 | 2 (0.6) | 49 (16.6) | 86 (44.7) | 123 (84.9) | 40 (98.0) | 5 (99.6) | 1 (100) | 0 | 0 | 0 |
| Tobramycin | 6.9 | 93.1 | 0 | 0 | 9 (2.9) | 8 (5.5) | 2 (6.2) | 2 (6.8) | 5 (8.5) | 42 (22.2) | 238 (100) | 0 | 0 |
| Minocycline^†^ | 31.0 | 44.4 | 0 | 0 | 0 | 2 (0.6) | 19 (16.8) | 35 (18.3) | 39 (31.0) | 75 (55.5) | 64 (76.5) | 72 (100) | 0 |

^*^ FDA breakpoints have been used

^†^ CLSI breakpoints have been used

EUCAST, European Committee on Antimicrobial Susceptibility Testing; MIC, Minimum Inhibitory Concentration; MIC_50_, minimum concentration required to inhibit 50% of the organisms; MIC_90_, minimum concentration required to inhibit 90% of the organisms; S, Susceptibility; R, Resistance

**Supplementary Table 14:** Antimicrobial activity of aztreonam-avibactam and comparator agents against MBL-positive *Enterobacterales* isolated from BSI collected across regions from 2021-2022

| **Antimicrobial agent** | **Susceptibility (EUCAST)** | | **No. of isolates inhibited at respective MIC (mg/L) (% cumulative inhibition)** | | | | | | | | | | |
| --- | --- | --- | --- | --- | --- | --- | --- | --- | --- | --- | --- | --- | --- |
|  | **%S** | **%R** | **≤0.06** | **0.12** | **0.25** | **0.5** | **1** | **2** | **4** | **8** | **16** | **32** | **>32** |
| **AfME (N=95)** | | | | | | | | | | | | | |
| **Aztreonam/**  **avibactam** | 100 | 0 | 19 (20.0) | 21 (42.1) | 34 (77.8) | 17 (95.7) | 3 (98.9) | 1 (100) |  |  |  |  |  |
| Aztreonam | 8.4 | 88.4 | 3 (3.1) | 1 (4.2) | 0 (4.2) | 3 (7.3) | 1 (8.4) | 1 (9.4) | 2 (11.5) | 1 (12.6) | 1 (13.6) | 4 (17.8) | 78 (100) |
| Cefepime | 0 | 100 |  |  |  |  |  |  |  | 1 (1.0) | 1 (2.1) | 93 (100) |  |
| Cefiderocol | 58.9 | 41.1 |  | 1 (1.0) | 0 (1.0) | 1 (2.1) | 18 (21.0) | 36 (58.9) | 34 (94.7) | 2 (96.8) | 2 (98.9) | 1 (100) |  |
| Colistin | 86.3^‡^ | 13.7 |  |  | 54 (56.8) | 23 (81.0) | 3 (84.2) | 2 (86.3) | 0 (86.3) | 13 (100) |  |  |  |
| Ertapenem | 1.0 | 99.0 |  |  | 1 (1.0) | 0 (1.0) | 1 (2.1) | 0 (2.1) | 1 (3.1) | 92 (100) |  |  |  |
| Tigecycline* | 46.3^‡^ | 53.7 | 1 (1.0) | 3 (4.2) | 13 (17.8) | 27 (46.3) | 42 (90.5) | 5 (95.7) | 4 (100.0) |  |  |  |  |
| Tobramycin | 3.1 | 96.9 |  | 1 (1.0) | 0 (1.0) | 1 (2.1) | 1 (3.1) | 0 (3.1) | 1 (4.2) | 91 (100.0) |  |  |  |
| Minocycline^†^ | 27.4 | 40.0 |  |  |  | 1 (1.0) | 3 (4.2) | 9 (13.6) | 13 (27.3) | 31 (60.0) | 38 (100.0) |  |  |
| **APAC (N=169)** | | | | | | | | | | | | | |
|  | **%S** | **%R** | **≤0.06** | **0.12** | **0.25** | **0.5** | **1** | **2** | **4** | **8** | **16** | **32** | **>32** |
| **Aztreonam/**  **avibactam** | 92.3 | 7.7 | 23 (13.6) | 20 (25.4) | 32 (44.3) | 49 (73.3) | 18 (84.0) | 4 (86.3) | 10 (92.3) | 7 (96.4) | 4 (98.8) | 0 (98.8) | 2 (100) |
| Aztreonam | 11.8 | 87.0 | 4 (2.3) | 3 (4.1) | 7 (8.2) | 2 (9.4) | 4 (11.8) | 1 (12.4) | 1 (13.0) | 2 (14.2) | 2 (15.3) | 14 (23.6) | 129 (100) |
| Cefepime | 0.6 | 98.2 |  | 1 (0.5) | 0 (0.5) | 0 (0.5) | 0 (0.5) | 0 (0.5) | 2 (1.7) | 0 (0.5) | 2 (2.9) | 164 (100) |  |
| Cefiderocol | 57.4 | 42.6 |  |  | 1(0.5) | 5 (3.5) | 22 (16.5) | 69 (57.4) | 48 (85.8) | 9 (91.1) | 1 (91.7) | 14 (100) |  |
| Colistin | 89.9^‡^ | 10.1 |  | 3 (1.7) | 113 (68.6) | 33 (88.1) | 2 (89.3) | 1 (89.9) | 0 (89.9) | 17 (100) |  |  |  |
| Ertapenem | 1.2 | 98.8 | 2 (1.1) | 0 (1.1) | 0 (1.1) | 0 (1.1) | 1 (1.7) | 5 (4.7) | 1 (5.3) | 160 (100) |  |  |  |
| Tigecycline* | 56.2^‡^ | 43.8 |  | 20 (11.8) | 38 (34.3) | 37 (56.2) | 47 (84.0) | 24 (98.2) | 3 (100) |  |  |  |  |
| Tobramycin | 18.3 | 81.7 |  |  | 9 (5.3) | 14 (13.6) | 5 (16.5) | 3 (18.3) | 4 (20.7) | 134 (100.0) |  |  |  |
| Minocycline^†^ | 40.8 | 33.1 |  |  | 2 (1.1) | 5 (4.1) | 21 (16.5) | 27 (32.5) | 14 (40.8) | 44 (66.8) | 56 (100) |  |  |
| **Europe (N=70)** | | | | | | | | | | | | | |
|  | **%S** | **%R** | **≤0.06** | **0.12** | **0.25** | **0.5** | **1** | **2** | **4** | **8** | **16** | **32** | **>32** |
| **Aztreonam/**  **avibactam** | 100 | 0 | 7 (10.0) | 14 (30.0) | 17 (54.2) | 22 (85.7) | 5 (92.8) | 5 (100) |  |  |  |  |  |
| Aztreonam | 5.7 | 88.6 |  |  | 2 (2.8) | 2 (5.7) | 0 (5.7) | 3 (10.0) | 1 (11.4) | 3 (15.7) | 2 (18.5) | 4 (24.2) | 53 (100) |
| Cefepime | 0 | 98.6 |  |  |  |  |  |  | 1 (1.4) | 1 (2.8) | 5 (10.0) | 63 (100) |  |
| Cefiderocol | 35.7 | 64.3 |  |  |  | 7 (10.0) | 2 (12.8) | 16 (35.7) | 34 (84.2) | 5 (91.4) | 4 (97.1) | 2 (100) |  |
| Colistin | 62.8^‡^ | 37.2 |  | 1 (1.4) | 30 (44.2) | 10 (58.5) | 2 (61.4) | 1 (62.8) | 3 (67.1) | 23 (100) |  |  |  |
| Ertapenem | 2.8 | 97.2 | 1 (1.4) | 0 1 (1.4) | 0 1 (1.4) | 1 (2.8) | 0 (2.8) | 4 (8.5) | 0 (8.5) | 0 (8.5) | 0 (8.5) | 0 (8.5) | 64 (100) |
| Tigecycline* | 48.6^‡^ | 51.4 |  | 1 (1.4) | 7 (11.4) | 26 (48.5) | 24 (82.8) | 10 (97.1) | 1 (98.5) | 1 (100) |  |  |  |
| Tobramycin | 4.3 | 95.7 |  |  | 1 (1.4) | 0 (1.4) | 2 (4.2) | 0 (4.2) | 3 (8.5) | 64 (100) |  |  |  |
| Minocycline^†^ | 35.7 | 45.7 |  |  |  |  | 4 (5.7) | 10 (20.0) | 11 (35.7) | 13 (54.2) | 32 (100) |  |  |
| **Latin America (N=106)** | | | | | | | | | | | | | |
| **Aztreonam/**  **avibactam** | 100 | 0 | 29 (27.3) | 18 (44.3) | 24 (66.9) | 24 (89.6) | 8 (97.1) | 1 (98.1) | 2 (100) |  |  |  |  |
| Aztreonam | 15.1 | 82.1 | 6 (5.6) | 1 (6.6) | 6 (12.2) | 0 (12.2) | 3 (15.0) | 1 (16.0) | 2 (17.9) | 3 (20.7) | 1 (21.7) | 8 (29.2) | 75 (100) |
| Cefepime | 0 | 99.0 |  |  |  |  |  | 1 (0.9) | 0 (0.9) | 1 (1.8) | 4 (5.6) | 100 (100) |  |
| Cefiderocol | 50.9 | 49.1 |  | 2 (1.8) | 1 (2.8) | 4 (6.6) | 15 (20.7) | 32 (50.9) | 38 (86.7) | 10 (96.2) | 1 (97.1) | 3 (100) |  |
| Colistin | 71.7^‡^ | 28.3 |  | 1 (0.9) | 44 (42.4) | 24 (65.0) | 5 (69.8) | 2 (71.7) | 0 (71.7) | 30 (100) |  |  |  |
| Ertapenem | 0 | 100 |  |  |  |  | 2 (1.8) | 0 (1.8) | 1 (2.8) | 103 (100) |  |  |  |
| Tigecycline* | 52.8^‡^ | 47.2 | 1 (0.9) | 3 (3.7) | 33 (34.9) | 19 (52.8) | 32 (83.0) | 16 (98.1) | 2 (100) |  |  |  |  |
| Tobramycin | 6.6 | 93.4 |  |  | 2 (1.8) | 4 (5.6) | 1 (6.6) | 0 (6.6) | 5 (11.3) | 94 (100) |  |  |  |
| Minocycline^†^ | 39.6 | 44.3 |  |  |  |  | 9 (8.4) | 13 (20.7) | 20 (39.6) | 17 (55.6) | 47 (100) |  |  |

^*^FDA breakpoints have been used

^†^ CLSI breakpoint have been used

^‡^Intrinsic resistance to colistin and tigecycline has been noted in *Proteus spp, Providencia spp., M. morganii* and *S. marcescens* and may contribute to elevated

*Enterobacterales* included the following genera: *Escherichia*, *Citrobacter*, *Enterobacter*, *Klebsiella*, *Proteus*, *Providencia*, *Morganella* and *Serratia*

AfME, Africa and Middle East; APAC, Asia Pacific; EUCAST, European Committee on Antimicrobial Susceptibility Testing; MIC, Minimum Inhibitory Concentration; MIC_50_, minimum concentration required to inhibit 50% of the organisms; MIC_90_, minimum concentration required to inhibit 90% of the organisms; S, Susceptibility; R, Resistance

**Supplementary Table 15:** Antimicrobial activity of aztreonam-avibactam and comparators against NDM1 variants of MBL-positive *Enterobacterales* isolated from BSI, 2021-2022

| **Antimicrobial agent** | **Susceptibility (EUCAST)** | | **No. of isolates inhibited at respective MIC (mg/L) (% cumulative inhibition)** | | | | | | | | | | |
| --- | --- | --- | --- | --- | --- | --- | --- | --- | --- | --- | --- | --- | --- |
|  | **%S** | **%R** | **≤0.06** | **0.12** | **0.25** | **0.5** | **1** | **2** | **4** | **8** | **16** | **32** | **>32** |
| **NDM-1 (N=201)** | | | | | | | | | | | | | |
| **Aztreonam/avibactam** | **99** | **1** | **53(26.4)** | **35(43.8)** | **52(69.6)** | **43(91.0)** | **14(98.0)** | **2(99.0)** | **0(99.0)** | **1(99.5)** | **0(99.5)** | **0(99.5)** | **1(100)** |
| Aztreonam | 14.4 | 80.6 | 10(5.0) | 1(5.5) | 9(9.9) | 4(11.9) | 5(14.4) | 5(16.9) | 5(19.4) | 3(20.9) | 2(21.9) | 16(29.8) | 141(100) |
| Cefepime | 0 | 99.5 | 0 | 0 | 0 | 0 | 0 | 1(0.5) | 0(0.5) | 1(1.0) | 5(3.5) | 10(8.5) | 184(100) |
| Cefiderocol | 49.8 | 50.2 | 0 | 2(1.0) | 1(1.5) | 3(3.0) | 33(19.4) | 61(49.8) | 73(86.0) | 14(93.0) | 5(95.5) | 3(97.0) | 6(100) |
| Colistin | 76.6^‡^ | 23.4 | 0 | 0 | 100(49.8) | 45(72.1) | 7(75.6) | 2(76.6) | 3(78.1) | 8(82.1) | 36(>8) (100) |  |  |
| Ertapenem | 1.0 | 99.0 | 1(0.5) | 0(0.5) | 1(1.0) | 0(1.0) | 0(1.0) | 0(1.0) | 0(1.0) | 7(4.5) | 192(>8) (100) |  |  |
| Minocycline* | 36.3 | 49.8 |  |  |  | 1(0.5) | 16(8.5) | 33(24.9) | 23(36.3) | 28(50.2) | 39(69.7) | 61(100) |  |
| Tigecycline^†^ | 96^‡^ | 0 | 1(0.5) | 8(4.5) | 39(23.9) | 57(52.2) | 65(84.6) | 23(96) | 8(100) |  |  |  |  |
| Tobramycin | 9.5 | 90.5 |  |  | 8(4.0) | 8(8.0) | 1(8.5) | 2 (9.5) | 8(13.4) | 26(26.4) | 148(>8) (100) |  |  |

*CLSI breakpoints have been used

^†^ FDA breakpoints have been used

^‡^Intrinsic resistance to colistin and tigecycline has been noted in *Proteus spp, Providencia spp., M. morganii* and *S. marcescens* and may contribute to elevated

*Enterobacterales* included the following genera: *Escherichia*, *Citrobacter*, *Enterobacter*, *Klebsiella*, *Proteus*, *Providencia*, *Morganella* and *Serratia*

EUCAST, European Committee on Antimicrobial Susceptibility Testing; MIC, Minimum Inhibitory Concentration; MIC_50_, minimum concentration required to inhibit 50% of the organisms; MIC_90_, minimum concentration required to inhibit 90% of the organisms; S, Susceptibility; R, Resistance

**Supplementary Table 16:** Antimicrobial activity of aztreonam-avibactam and comparators against NDM5 variants of MBL-positive *Enterobacterales* isolated from BSI, 2021-2022

| **Antimicrobial agent** | **Susceptibility (EUCAST)** | | **No. of isolates inhibited at respective MIC (mg/L) (% cumulative inhibition)** | | | | | | | | | | |
| --- | --- | --- | --- | --- | --- | --- | --- | --- | --- | --- | --- | --- | --- |
|  | **%S** | **%R** | **≤0.06** | **0.12** | **0.25** | **0.5** | **1** | **2** | **4** | **8** | **16** | **32** | **>32** |
| **NDM-5 (N=174)** | | | | | | | | | | | | | |
| **Aztreonam/avibactam** | **93.7** | **6.3** | **16 (9.2)** | **28 (25.3)** | **40 (48.3)** | **46 (74.7)** | **18 (85.1)** | **5 (87.9)** | **10 (93.7)** | **6 (97.1)** | **4 (99.4)** | **0 (99.4)** | **1** **(100)** |
| Aztreonam | 5.2 | 94.8 | 1(0.6) | 2(1.7) | 2(2.9) | 1(3.4) | 3(5.2) | 0(5.2) | 0(5.2) | 2(6.3) | 2(7.5) | 9(12.6) | 152(100) |
| Cefepime | 0.6 | 99.4 | 0 | 1(0.6) | 0(0.6) | 0(0.6) | 0(0.6) | 0(0.6) | 0(0.6) | 1(1.2) | 0(1.2) | 5(4.0) | 167(100) |
| Cefiderocol | 54.6 | 45.4 | 0 | 0 | 1(0.6) | 4(2.9) | 15(11.5) | 75(54.6) | 60(89.0) | 7(93.1) | 2(94.2) | 0(94.2) | 10(100) |
| Colistin | 90.8^‡^ | 9.2 | 0 | 5(2.9) | 114(68.4) | 32(86.8) | 4(89.0) | 3(90.8) | 0(90.8) | 5(93.7) | 11(100) |  |  |
| Ertapenem | 0.6 | 99.4 | 1(0.6) | 0(0.6) | 0(0.6) | 0(0.6) | 0(0.6) | 0(0.6) | 1(1.2) | 0(1.2) | 172(>8) (100) |  |  |
| Minocycline* | 34.5 | 27.6 |  |  | 2(1.2) | 5(4) | 16(13.2) | 18(23.6) | 19(34.5) | 66(72.4) | 27(87.9) | 21(100) |  |
| Tigecycline^†^ | 98.8^‡^ | 0 | 1(0.6) | 16(9.8) | 39(32.2) | 38(54) | 54(85.1) | 24(98.8) | 2(100) |  |  |  |  |
| Tobramycin | 9.8 | 90.2 | 0 | 0 | 1(0.6) | 9(5.7) | 6(9.2) | 1(9.8) | 3(11.5) | 21(23.6) | 133(>8) (100) |  |  |

^*^CLSI breakpoints have been used

^†^FDA breakpoints have been used

^‡^Intrinsic resistance to colistin and tigecycline has been noted in *Proteus spp, Providencia spp., M. morganii* and *S. marcescens* and may contribute to elevated

*Enterobacterales* included the following genera: *Escherichia*, *Citrobacter*, *Enterobacter*, *Klebsiella*, *Proteus*, *Providencia*, *Morganella* and *Serratia*

EUCAST, European Committee on Antimicrobial Susceptibility Testing; MIC, Minimum Inhibitory Concentration; MIC_50_, minimum concentration required to inhibit 50% of the organisms; MIC_90_, minimum concentration required to inhibit 90% of the organisms; S, Susceptibility; R, Resistance

**Supplementary Table 17:** Antimicrobial activity of aztreonam-avibactam and comparator agents against NDM-positive *E. coli* and *K. pneumoniae* isolated from BSI, 2021-2022

| **Antimicrobial agent** | **Susceptibility (EUCAST)** | | **No. of isolates inhibited at respective MIC (mg/L) (% cumulative inhibition)** | | | | | | | | | | |
| --- | --- | --- | --- | --- | --- | --- | --- | --- | --- | --- | --- | --- | --- |
|  | **%S** | **%R** | **≤0.06** | **0.12** | **0.25** | **0.5** | **1** | **2** | **4** | **8** | **16** | **32** | **>32** |
| ***E. coli* (N=48)** | | | | | | | | | | | | | |
| **Aztreonam/**  **avibactam** | 75.0 | 25.0 | 9(18.8) | 1(20.8) | 1(22.9) | 0(22.9) | 7(37.5) | 6(50.0) | 12(75.0) | 6(87.5) | 4(95.8) | 0(95.8) | 2(100) |
| Aztreonam | 6.2 | 93.8 | 0 | 1(2.1) | 2(6.2) | 0(6.2) | 0(6.2) | 0(6.2) | 0(6.2) | 3(12.5) | 1(14.6) | 6(27.1) | 35(100) |
| Cefepime | 0 | 100 |  |  |  |  |  |  |  | 1(2.1) | 0(2.1) | 2(6.2) | 45(100) |
| Cefiderocol | 35.4 | 64.6 |  |  |  |  | 1(2.1) | 16(35.4) | 13(62.5) | 6(75.0) | 1(77.1) | 0(77.1) | 11(100) |
| Colistin | 100 | 0 |  | 5(10.4) | 36(85.4) | 6(97.9) | 1(100) |  |  |  |  |  |  |
| Ertapenem | 0 | 100 |  |  |  |  |  |  |  | 1(2.1) | 47(100) |  |  |
| Tigecycline* | 100 | 6.3 | 1(2.1) | 18(39.6) | 19(79.2) | 7(93.7) | 3(100) |  |  |  |  |  |  |
| Tobramycin | 27.1 | 72.9 |  |  | 2(4.2) | 6(16.7) | 5(27.1) | 0(27.1) | 0(27.1) | 2(31.2) | 33(100) |  |  |
| Minocycline^†^ | 50 | 18.8 |  |  | 2(4.2) | 4(12.5) | 9(31.2) | 2(35.4) | 7(50) | 15(81.2) | 4(89.6) | 5(100) |  |
| ***K. pneumoniae* (N=291)** | | | | | | | | | | | | | |
| **Aztreonam/**  **avibactam** | 100 | 0 | 33(11.3) | 53(29.5) | 94(61.9) | 91(93.1) | 19(99.7) | 1(100) |  |  |  |  |  |
| Aztreonam | 6.9 | 91.4 | 6(2.1) | 1(2.4) | 6(4.5) | 4(5.8) | 3(6.9) | 3(7.9) | 2(8.6) | 2(9.3) | 2(10.0) | 10(13.4) | 252(100) |
| Cefepime | 0.3 | 99.7 |  | 1(0.3) | 1(0.3) | 0(0.3) | 0(0.3) | 0(0.3) | 0(0.3) | 0(0.3) | 2(1.0) | 10(4.5) | 278(100) |
| Cefiderocol | 55.0 | 45.0 |  |  | 1(0.3) | 4(1.7) | 35(13.7) | 120(55.0) | 112(93.5) | 14(98.3) | 2(99.0) | 0(99.0) | 3(100) |
| Colistin | 81.4 | 18.6 |  |  | 157(53.9) | 66(76.6) | 9(79.7) | 5(81.4) | 3(82.5) | 17(88.3) | 34(100) |  |  |
| Ertapenem | 0.7 | 99.3 | 1(0.3) | 0(0.3) | 1(0.7) | 0(0.7) | 0(0.7) | 0(0.7) | 1(1.0) | 0(1.0) | 288(100) |  |  |
| Tigecycline* | 98.3 | 0 |  | 2(0.7) | 48(17.2) | 86(46.7) | 112(85.2) | 38(98.3) | 5(100) |  |  |  |  |
| Tobramycin | 6.9 | 93.1 |  |  | 9(3.1) | 8(5.8) | 1(6.2) | 2(6.9) | 5(8.6) | 41(22.7) | 225(100) |  |  |
| Minocycline^†^ | 29.9 | 44.3 |  |  |  | 2(0.7) | 19(7.2) | 34(18.9) | 32(29.9) | 75(55.7) | 63(77.3) | 66(100) |  |

^*^ FDA breakpoint have been used

^†^CLSI breakpoint have been used

EUCAST, European Committee on Antimicrobial Susceptibility Testing; MIC, Minimum Inhibitory Concentration; MIC_50_, minimum concentration required to inhibit 50% of the organisms; MIC_90_, minimum concentration required to inhibit 90% of the organisms; S, Susceptibility; R, Resistance

**Supplementary Table 18:** Antimicrobial activity of aztreonam-avibactam and comparator agents against NDM-positive *Enterobacterales* isolated from BSI collected across regions from 2021-2022

| **Antimicrobial agent** | **Susceptibility (EUCAST)** | | **No. of isolates inhibited at respective MIC (mg/L) (% cumulative inhibition)** | | | | | | | | | | |
| --- | --- | --- | --- | --- | --- | --- | --- | --- | --- | --- | --- | --- | --- |
|  | **%S** | **%R** | **≤0.06** | **0.12** | **0.25** | **0.5** | **1** | **2** | **4** | **8** | **16** | **32** | **>32** |
| **AfME (N=95)** | | | | | | | | | | | | | |
| **Aztreonam/**  **avibactam** | 100 | 0 | 19 (20.0) | 21 (42.1) | 34 (77.9) | 17 (95.8) | 3 (98.9) | 1 (100) |  |  |  |  |  |
| Aztreonam | 8.4 | 88.4 | 3(3.2) | 1(4.2) | 0(4.2) | 3(7.4) | 1(8.4) | 1(9.5) | 2(11.6) | 1(12.6) | 1(13.7) | 4(17.9) | 78(100) |
| Cefepime | 0 | 100 |  |  |  |  |  |  |  | 1(1.1) | 1(2.1) | 5(7.4) | 88(100) |
| Cefiderocol | 58.9 | 41.1 |  | 1(1.1) | 0(1.1) | 1(2.1) | 18(21.1) | 36(58.9) | 34(94.7) | 2(96.8) | 2(98.9) | 0(98.9) | 1(100) |
| Colistin | 86.3^‡^ | 13.7 |  |  | 54(56.8) | 23(81.1) | 3(84.2) | 2(86.3) | 0(86.3) | 1(87.4) | 12(100) |  |  |
| Ertapenem | 1.1 | 98.9 |  |  | 1(1.1) | 0(1.1) | 1(2.1) | 0(2.1) | 1(3.2) | 2(5.3) | 90(100) |  |  |
| Tigecycline* | 95.8^‡^ | 0 | 1(1.1) | 3(4.2) | 13(17.9) | 27(46.3) | 42(90.5) | 5(95.8) | 4(100) |  |  |  |  |
| Tobramycin | 3.2 | 96.8 |  | 1(1.1) | 0(1.1) | 1(2.1) | 1(3.2) | 0(3.2) | 1(4.2) | 17(22.1) | 74(100) |  |  |
| Minocycline^†^ | 27.4 | 40.0 |  |  |  | 1(1.1) | 3(4.2) | 9(13.7) | 13(27.4) | 31(60) | 17(77.9) | 21(100) |  |
| **APAC (N=160)** | | | | | | | | | | | | | |
| **Aztreonam/**  **avibactam** | 91.9 | 8.1 | 22(13.8) | 19(25.6) | 29(43.8) | 46(72.5) | 17(83.1) | 4(85.6) | 10(91.9) | 7(96.3) | 4(98.8) | 0(98.8) | 2(100) |
| Aztreonam | 10.0 | 88.8 | 3(1.9) | 2(3.1) | 6(6.9) | 1(7.5) | 4(10) | 1(10.6) | 1(11.3) | 2(12.5) | 2(13.8) | 13(21.9) | 125(100) |
| Cefepime | 0.6 | 99.4 |  | 1(0.6) | 0(0.6) | 0(0.6) | 0(0.6) | 0(0.6) | 0(0.6) | 0(0.6) | 1(1.3) | 3(3.1) | 155(100) |
| Cefiderocol | 56.9 | 43.1 |  |  | 1(0.6) | 1(1.3) | 20(13.8) | 69(56.9) | 45(85.0) | 9(90.6) | 1(91.3) | 1(91.9) | 13(100) |
| Colistin | 89.4^‡^ | 10.6 |  | 3(1.9) | 105(67.5) | 32(87.5) | 2(88.8) | 1(89.4) | 0(89.4) | 2(90.6) | 15(100) |  |  |
| Ertapenem | 1.2 | 98.8 | 2(1.3) | 0(1.3) | 0(1.3) | 0(1.3) | 0(1.3) | 0(1.3) | 0(1.3) | 2(2.5) | 156(100) |  |  |
| Tigecycline* | 98.1^‡^ | 0 |  | 19(11.9) | 37(35.0) | 34(56.3) | 45(84.4) | 22(98.1) | 3(100) |  |  |  |  |
| Tobramycin | 18.7 | 81.3 |  |  | 9(5.6) | 13(13.8) | 5(16.9) | 3(18.8) | 4(21.3) | 11(28.1) | 115(100) |  |  |
| Minocycline^†^ | 40.0 | 32.5 |  |  | 2(1.3) | 5(4.4) | 20(16.9) | 24(31.9) | 13(40.0) | 44(67.5) | 23(81.9) | 29(100) |  |
| **Europe (N=57)** | | | | | | | | | | | | | |
|  | **%S** | **%R** | **≤0.06** | **0.12** | **0.25** | **0.5** | **1** | **2** | **4** | **8** | **16** | **32** | **>32** |
| **Aztreonam/**  **avibactam** | 100 | 0 | 6(10.5) | 9(26.3) | 17(56.1) | 19(89.5) | 4(96.5) | 2(100) |  |  |  |  |  |
| Aztreonam | 5.3 | 89.5 |  |  | 1(1.8) | 2(5.3) | 0(5.3) | 3(10.5) | 0(10.5) | 1(12.3) | 1(14.0) | 2(17.5) | 47(100) |
| Cefepime | 0 | 100 |  |  |  |  |  |  |  |  | 2(3.5) | 3(8.8) | 52(100) |
| Cefiderocol | 26.3 | 73.7 |  |  |  | 2(3.5) | 0(3.5) | 13(26.3) | 32(82.5) | 5(91.2) | 3(96.5) | 0(96.5) | 2(100) |
| Colistin | 59.6^‡^ | 40.4 |  | 1(1.8) | 24(43.9) | 6(54.4) | 2(57.9) | 1(59.6) | 3(64.9) | 9(80.7) | 11(100) |  |  |
| Ertapenem | 0 | 100 | 0 | 0 | 0 | 0 | 0 | 0 | 0 | 0 | 57(100) |  |  |
| Tigecycline* | 98.2^‡^ | 0 |  | 1(1.8) | 6(12.3) | 23(52.6) | 20(87.7) | 6(98.2) | 1(100) |  |  |  |  |
| Tobramycin | 1.8 | 98.2 |  |  | 1(1.8) | 0(1.8) | 0(1.8) | 0(1.8) | 2(5.3) | 10(22.8) | 44(100) |  |  |
| Minocycline^†^ | 35.1 | 45.6 |  |  |  |  | 3(5.3) | 8(19.3) | 9(35.1) | 11(54.4) | 13(77.2) | 13(100) |  |
| **Latin America (N=95)** | | | | | | | | | | | | | |
| **Aztreonam/**  **avibactam** | 100 | 0 | 27(28.4) | 17(46.3) | 23(70.5) | 17(88.4) | 8(96.8) | 1(97.9) | 2(100) |  |  |  |  |
| Aztreonam | 14.7 | 83.2 | 6(6.3) | 0(6.3) | 5(11.6) | 0(11.6) | 3(14.7) | 0(14.7) | 2(16.8) | 2(18.9) | 1(20.0) | 8(28.4) | 68(100) |
| Cefepime | 0 | 98.9 |  |  |  |  |  | 1(1.1) | 0(1.1) | 1(2.1) | 2(4.2) | 4(8.4) | 87(100) |
| Cefiderocol | 48.4 | 51.6 |  | 2(2.1) | 1(3.2) | 3(6.3) | 11(17.9) | 29(48.4) | 35(85.3) | 10(95.8) | 1(96.8) | 2(98.9) | 1(100) |
| Colistin | 75.8^‡^ | 24.2 |  | 1(1.1) | 42(45.3) | 22(68.4) | 5(73.7) | 2(75.8) | 0(75.8) | 8(84.2) | 15(100) |  |  |
| Ertapenem | 0 | 100 |  |  |  |  |  |  |  | 3(3.2) | 92(100) |  |  |
| Tigecycline* | 97.9^‡^ | 0 | 1(1.1) | 2(3.2) | 30(34.7) | 19(54.7) | 25(81.1) | 16(97.9) | 2(100) |  |  |  |  |
| Tobramycin | 7.4 | 92.6 |  |  | 2(2.1) | 4(6.3) | 1(7.4) | 0(7.4) | 4(11.6) | 12(24.2) | 72(100) |  |  |
| Minocycline^†^ | 33.7 | 48.4 |  |  |  |  | 7(7.4) | 12(20.0) | 13(33.7) | 17(51.6) | 21(73.7) | 25(100) |  |

^*^ FDA breakpoint have been used

^†^CLSI breakpoint have been used

^‡^Intrinsic resistance to colistin and tigecycline has been noted in *Proteus spp, Providencia spp., M. morganii* and *S. marcescens* and may contribute to elevated

*Enterobacterales* included the following genera: *Escherichia*, *Citrobacter*, *Enterobacter*, *Klebsiella*, *Proteus*, *Providencia*, *Morganella* and *Serratia*

AfME, Africa and Middle East; APAC, Asia Pacific; EUCAST, European Committee on Antimicrobial Susceptibility Testing; MIC, Minimum Inhibitory Concentration; MIC_50_, minimum concentration required to inhibit 50% of the organisms; MIC_90_, minimum concentration required to inhibit 90% of the organisms; S, Susceptibility; R, Resistance
